# Assessment of the kidney and lung as immune barriers and hematopoietic sites in the invasive apple snail *Pomacea canaliculata*

**DOI:** 10.7717/peerj.5789

**Published:** 2018-10-12

**Authors:** Cristian Rodriguez, Guido I. Prieto, Israel A. Vega, Alfredo Castro-Vazquez

**Affiliations:** 1 IHEM, CONICET, Universidad Nacional de Cuyo, Mendoza, Argentina; 2 Departamento de Biología, Facultad de Ciencias Exactas y Naturales, Universidad Nacional de Cuyo, Mendoza, Argentina; 3 Instituto de Fisiología, Facultad de Ciencias Médicas, Universidad Nacional de Cuyo, Mendoza, Argentina

**Keywords:** Gastropod, Hemocyte nodules, Cell proliferation, Circulatory system, Ampullariidae, *Angiostrongylus cantonensis*, Eosinophilic meningitis, Biological invasions

## Abstract

Knowledge on the immune system of *Pomacea canaliculata* is becoming increasingly important, because of this gastropod’s role as intermediate host and vector of *Angiostrongylus cantonensis*, the etiologic agent of eosinophilic meningitis in humans and domestic animals. Immune defenses of this gastropod comprise both humoral and cellular components, but they may also involve organs that act as immune barriers to prevent the spread of alien molecules and organisms. Both the kidney and lung are here shown to serve this function, because of (1) their positions in blood circulation, (2) the intricate architecture of their blood spaces, and (3) the proliferative and nodulation reactions of hemocytes to an immune challenge. However, these organs differ in that only the kidney shows permanent hemocyte aggregations. Microcirculation in the kidney was found to flow through an intricate vascular bed containing the permanent aggregations, which occurred either as hemocyte islets anchored by cytoplasmic projections of the renal epithelium or as perivascular accretions. Within 96 h of the injection of yeast cells, hemocyte nodules were formed both in the kidney and lung. Moreover, cell proliferation in renal hemocyte islets was measured by bromodeoxyuridine (BrdU) incorporation. The proportion of BrdU positive nuclei increased 48 h after injection. Signs of nodule regression (apoptotic bodies, lipofuscin-like deposits) and a decrease in the proportion of BrdU positive nuclei were found at 96 h. In addition, the area of renal hemocyte islets was significantly increased 96 h after injection. Nevertheless, the high complexity of the small vascular chambers that constitute the lung’s respiratory lamina would also facilitate hemocyte-antigen contacts, required to elicit cellular aggregation, and hence, nodulation. To our knowledge, this paper includes the first quantitative indication of hemocyte proliferation after an immune challenge among Caenogastropoda.

## Introduction

Gastropod hemocytes recognize and phagocytize invaders, entrap them through nodulation (Sminia, 1981) and/or they also encapsulate foreign objects too large to be engulfed (Godoy et al., 1997; Lemos & Andrade, 2001; Lv et al., 2009b; Sminia, Borghart-Reinders & Van de Linde, 1974; Van der Knaap, Adema & Sminia, 1993; Yousif, Blähser & Lämmler, 1980). Knowledge on the immune system of *Pomacea canaliculata* (Lamarck 1822, Ampullariidae) is becoming increasingly important, because of the role of this species as an intermediate host and vector of the nematode *Angiostrongylus cantonensis* (Chen 1935, Metastrongylidae), the major etiologic agent of eosinophilic meningitis, a parasitic disease that can be disabling in humans, and may be even fatal (Cowie, 2017; Martins, Tanowitz & Kazacos, 2015). While *P. canaliculata*, a native of the lower Río de la Plata basin, has invaded China (as well as several other countries, Lv et al., 2009a; Soriano, Salgado & Tarruella, 2009), *A. cantonensis* is now present in Brazil (Morassutti et al., 2014; Thiengo et al., 2013; Valente et al., 2018), i.e., on the verge to overlap the native range of *P. canaliculata*, which would facilitate the spread of the parasite.

Hemocyte-antigen encounters may occur in the systemic blood circulation, but we hypothesized that there are organs acting as immune barriers, whose characteristics may facilitate these encounters. Such characteristics would be: (1) an adequate position of the organ in blood circulation, so as to prevent antigen dissemination, and/or (2) an intricate microcirculation, which would increase the probability of hemocyte-antigen contacts, and/or (3) a locally high hemocyte concentration.

A more general comparative interest in the immune system of *P. canaliculata* is that this species is one of the two ampullariids whose immune system has been studied to some extent (Accorsi et al., 2017; Accorsi et al., 2013; Accorsi, Ottaviani & Malagoli, 2014; Cueto et al., 2015; Cueto, Vega & Castro-Vazquez, 2013; Shozawa & Suto, 1990; Sun et al., 2013; Yousif, Blähser & Lämmler, 1980). In fact, the Ampullariidae are part of the clade Caenogastropoda, which encompasses about 60% of extant gastropod species, and gastropod immunobiology may be biased because it is based on just a few families of Panpulmonata (Heterobranchia), i.e., on a small segment of gastropod diversity (Loker, 2010).

This paper deals with blood circulation and cellular reactions of the kidney and lung of *P. canaliculata* as possible immune barriers and hematopoietic sites. In this paper, we have chosen a non-pathogenic immune challenge (i.e., yeast injection) to observe the mere reaction to foreign cells, with no accompanying damage as it occurs when pathogens are employed (e.g.,  Rodriguez & Castro-Vazquez, 2016). Nodular aggregation of hemocytes was shown in both the kidney and lung as a conspicuous reaction to yeast injection. Finally, proliferation in the renal hemocyte islets has been quantified after an immune challenge, for the first time in a proposed hematopoietic site of a caenogastropod.

## Materials and Methods

### Animals and culture conditions

*P. canaliculata* adult males (4-months-old, 35–40 mm in shell length) from the Rosedal strain were used. The origin of the strain and the culturing conditions have been reported several times elsewhere (e.g.,  Cueto et al., 2015). Briefly, animals were kept in aquaria under controlled temperature (24–26 °C) and photoperiod (14 h light:10 h dark cycle), and were fed *ad libitum* with a diet composed of fresh lettuce, carp food pellets (Peishe Car Shulet, Argentina), desiccated and powdered *P. canaliculata*’s eggs, and toilet paper (Higienol®, Argentina). Collection of the original stock of the Rosedal strain did not require specific permits since it is an invasive, not an endangered species (IUCN Red List status “Least concern”; population trend: “increasing”). Procedures for snail culture, sacrifice, and tissue sampling were approved by the Institutional Committee for the Care and Use of Laboratory Animals (Comité Institucional para el Cuidado y Uso de Animales de Laboratorio (CICUAL), Facultad de Ciencias Médicas, Universidad Nacional de Cuyo), Approval Protocol No 55/2015.

### Tissue sampling and procedures for light and electron microscopy

Animals were put in an ice/water bath (∼4 °C, for 20–30 min), both for inducing relaxation and minimizing pain, before carefully cracking the shell.

For light microscopy, samples of the kidney (‘posterior kidney’ in Andrews (1965), Hylton Scott (1957)) and of the lung were obtained and then were fixed in diluted Bouin’s fluid (1:2) for 5–7 days at 4 °C. Tissues were dehydrated in an ethanol series, cleared with xylene and embedded in a resin-paraffin mixture (1:1, Histoplast®, Argentina). The microtome sections were observed either unstained or stained with Gill’s hematoxylin and eosin.

For electron microscopy, small pieces of the kidney (*N* = 3) were fixed in Karnovsky’s fluid (4% paraformaldehyde, 2.5% glutaraldehyde in 0.1 M phosphate buffer; pH = 7.4) for 6 h and post-fixed in 2% osmium tetroxide overnight. Samples were rinsed in phosphate buffer, dehydrated in a graded acetone series and finally embedded in Spurr’s resin. Then, thin sections (∼200 nm) were stained with toluidine blue and mounted on glass slides with DPX medium (Cat. #44581; Sigma-Aldrich, St. Louis, MO, USA) and examined and photographed under a Nikon Eclipse 80i microscope using Nikon DS-Fi1-U3 camera and Nikon NIS-ELEMENT Image Software for image acquisition. Ultrathin sections (∼70 nm) were mounted on copper grids, stained with uranyl acetate and lead citrate and examined with a Zeiss EM 900 transmission electron microscope.

### Inoculation

A yeast cell suspension (*Saccharomyces cerevisiae*; Levex®, Buenos Aires, Argentina) was prepared in a suitable apyrogenic buffer (PcBS, Cueto et al., 2015) which was passed through a Millipore® 0.22 µm filter before use and then was adjusted to ∼1.26 × 10^5^ cells/mL using a Fuchs–Rosenthal hemocytometer. Eighty µL of this suspension (or its vehicle) were injected into treated or control animals respectively. All injections were made in the visceral hemocoel through a small hole (∼1 mm) in the shell, which was drilled manually at midway between sutures of the second whorl (Fig. S1). The tip of the injecting needle was introduced ∼4 mm below the shell surface, in the digestive gland close to the testis. No spillage of the injected material was observed and the whole procedure did not appear to be painful for the animals. The animals were then kept in individual aquaria until they were sacrificed 48 or 96 h later (according to the experiment).

### Quantitative changes in hemocyte proliferation

Proliferating cells in renal hemocyte islets were detected in control and treated animals by the indirect immunofluorescence (IIF) of 5-bromo-2-deoxyuridine (BrdU) in nuclei. Control and treated animals were injected with BrdU (80 µL of the stock solution, Roche; Cat. # 11 296 736 001) into the visceral hemocoel 42 and/or 90 h after injection of either *Pc* BS or yeast cells suspended in the same buffer. Groups (3 animals each) were: (1) control animals sacrificed 48 h after vehicle injection; (2) control animals sacrificed 96 h after vehicle injection; (3) treated animals sacrificed 48 h after yeast injection; and (4) treated animals sacrificed 96 h after yeast injection. Sampling times were chosen according to preliminary observations (Rodriguez et al., 2012).

The kidney was dissected out and fixed in 4% paraformaldehyde for 16 h. Lung samples were not processed, because this organ does not normally show hemocyte aggregates and so, the buffer-injected animals would not provide proper control tissues. Then the slides were passed through the following steps: (1) xylene-removal of the embedding medium, (2) rehydration of sections through an ethanol/water series to distilled water, (3) incubation in buffer (10 Mm sodium citrate, 0.05% Triton X-100; pH = 6.0) for 25 min at 95–100 °C, (4) washing in distilled water for 10 min followed by two steps in phosphate buffered saline (PBS; pH = 7.4), (5) incubation with a 1:10 dilution of an antibody against BrdU (Roche; Cat. # 11 296 736 001) for 30 min at 37 °C in humid chamber, (6) three washing steps (10 min each) in PBS, (7) incubation with a 1:10 dilution of anti-mouse-Ig-fluorescein antibody (Roche; Cat. # 11 296 736 001) in PBS for 30 min at 37 °C in humid chamber, (8) three washing steps in PBS, (9) counterstaining with 4′,6-diamidino-2-phenylindole (DAPI), (10) washing in PBS and (11) mounting with FluorSave® Reagent (Calbiochem).

Six images per animal (*N* = 3 animals per group) were captured at high magnification (60×) using an Olympus FV1000 confocal microscope and processed with Adobe Photoshop CS6 software. Total hemocyte nuclei (DAPI^+^) were counted using CellProfiler software (http://www.cellprofiler.org) and BrdU^+^ cells were counted manually by two independent researchers. Hemocyte nuclei were clearly distinguished from those of epithelial cells because of their size and the conspicuous nucleoli of the latter. Only BrdU^+^ labels that overlapped >50% with DAPI^+^ labeled nuclei were counted as BrdU^+^ nuclei.

Cell proliferation was assessed as the proportion of BrdU^+^ cells within the islets. Three kidneys per experimental group were processed. Data were expressed as mean ± SEM. Statistical significance of differences (*P* ≤ 0.05) was assessed with the one-tailed Mann–Whitney *U–* test, using GraphPad Prism 5.03 (GraphPad Software Inc., San Diego, CA, USA).

### Morphometric experiment

For renal islet morphometry, control and treated animals (*N* = 9 per group) were sacrificed 96 h after injection of either PcBS or yeast cells respectively, and kidney samples were obtained and processed as described above. In preliminary observations, distinct nodules with an outer layer of flattened cells were constantly found at this time (Rodriguez et al., 2012). A total of fifteen images (from sections 15–40 µm apart) were obtained from each kidney sample (Fig. S1) and the islets were outlined with Image Pro Plus v.6.0 (Media Cybernetics, Rockville, MD, USA) software using the polygon tool. The median total hemocyte and renal tissue areas (both expressed in µm^2^) were obtained for each animal to calculate the ‘hemocyte aggregates/total renal tissue’ ratio. Then the mean ± SEM of control and treated animals was computed. Statistical significance of differences (*P* ≤ 0.05) between control and treated animals were assessed by one-tailed Student’s *t*-test, using GraphPad Prism 5.03 software.

## Results

### Blood circulation in the normal kidney

**Figure 1:**
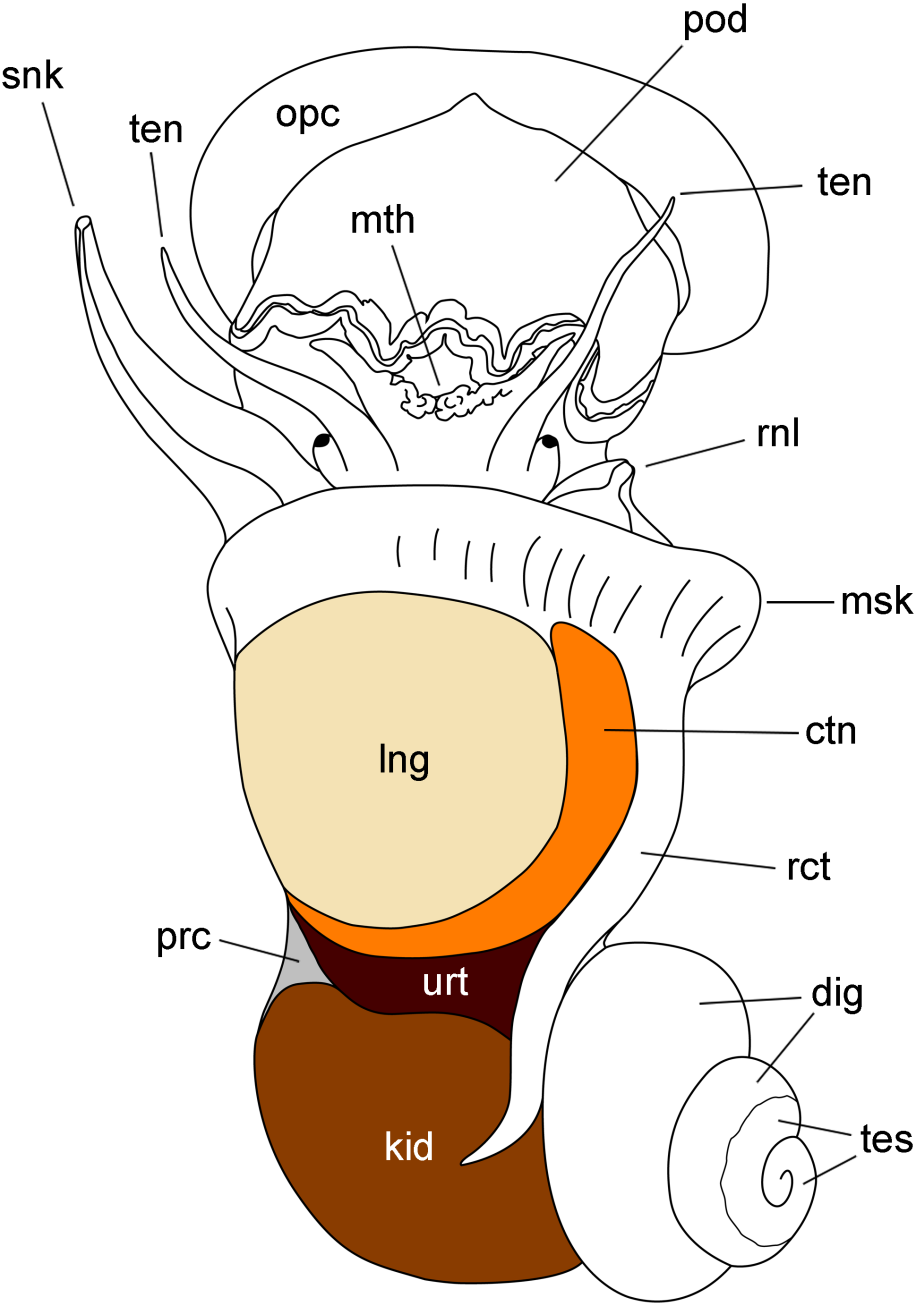
Outlines of the kidney, lung and other organs in a male individual with the shell removed (dorsal view). Abbreviations: ctn, gill; dig, digestive gland; kid, kidney; msk, mantle skirt; mth, mouth; opc, operculum; lng, lung; pod, foot; prc, pericardium; rct, rectum; rnl, right nuchal lobe; snk, siphon; ten, tentacle; tes, testis; urt, ureter.

The kidney of *P. canaliculata* (‘posterior kidney’ in Andrews, 1965; Hylton Scott, 1957) is a beret-shaped epithelial organ on the body surface, lying just behind the ureter (‘anterior kidney’ in Andrews (1965), Hylton Scott (1957)) and the mantle cavity (Fig. 1). It dorsally covers the renal chamber containing the coiled gut (Godoy, Castro-Vazquez & Vega, 2013) and is the modified homolog of the saccular metanephridium of most caenogastropods (Hyman, 1967).

**Figure 2:**
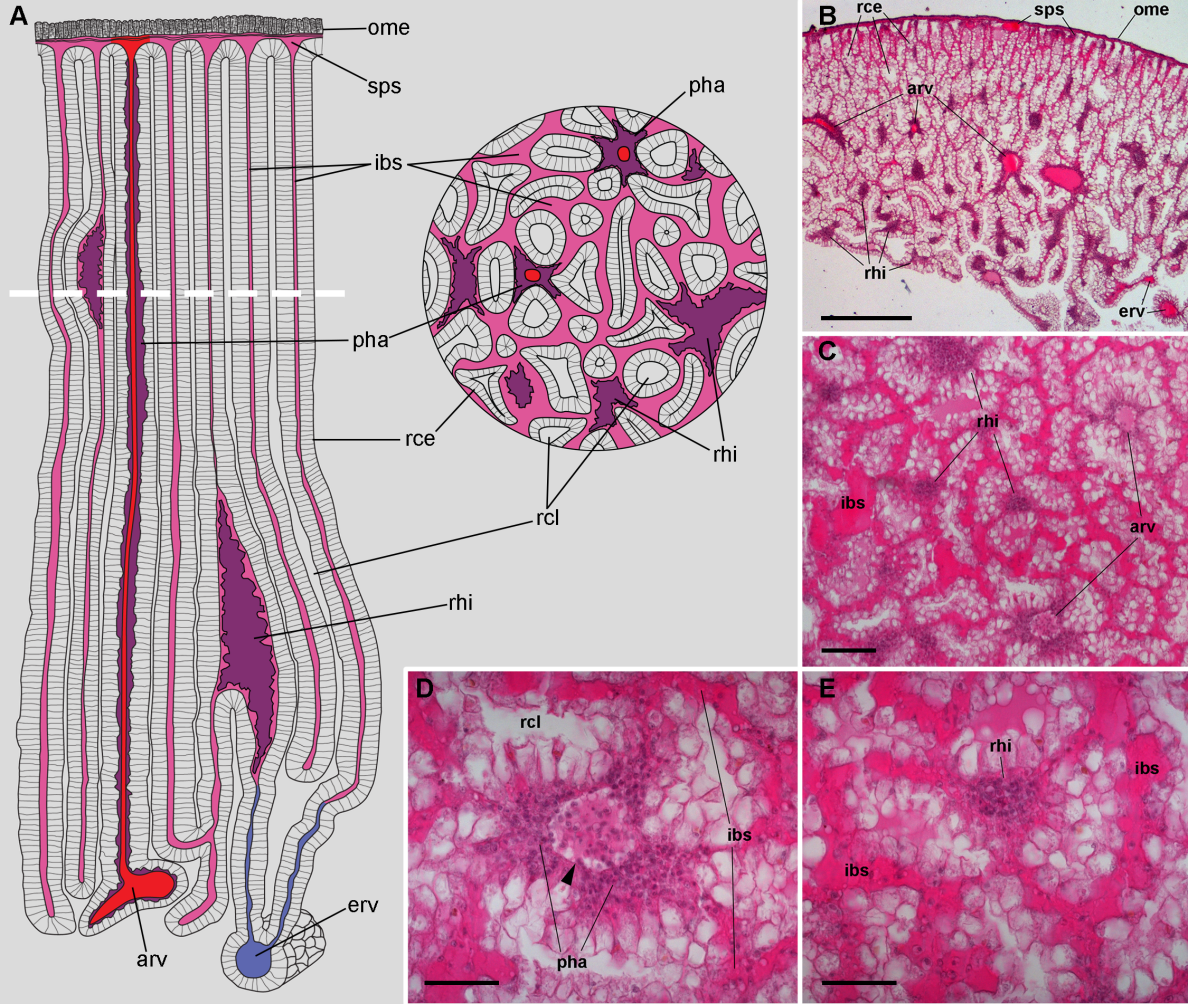
Renal microcirculation. (A) Diagrams of kidney sections, perpendicular to each other. Blood reaches the kidney through the greater afferent renal vessel, which gives off perpendicular vessels that go deep inside the organ (red). Perivascular hemocyte accretions (violet) are often found around the perpendicular afferent vessels. Blood then runs into a system of interconnected sinuses (pale pink) that surround the hemocyte crypts and also join beneath the muscular layer of the outer mantle. The hemocyte islets (violet) are scattered in these sinuses and are more frequent and larger towards the renal chamber, i.e., closer to the final convergence of the sinuses into the efferent vessels (blue). (B) Field view of a kidney section showing the long epithelial crypts, some afferent and efferent vessels, some sinuses, and numerous hemocyte aggregations. (C) Transverse section of epithelial crypts showing the continuity of blood sinuses. (D) Detail of an afferent perpendicular vessel showing a delimiting membrane (arrowhead) and the perivascular hemocyte accretions which extend to the nearby open sinuses. (E) Detail of a small hemocyte islet in the course of a blood sinus. Hematoxylin-eosin. Scale bars represent: (B) 500 µm; (C) 100 µm; (D) 50 µm; (E) 50 µm. Abbreviations: arv, greater afferent renal vessel; erv, greater efferent renal vessel; ibs, intercryptal blood sinus; ome, outer mantle epithelium; pha, perivascular hemocyte accretion; rce, renal cryptal epithelium; rcl, renal cryptal lumen; rhi, renal hemocyte islet; sps, subpallial blood sinus.

According to Andrews (1965), blood from the head and foot sinuses is conducted to the kidney by a single afferent vessel which gives off a branch supplying the ureter before ramifying into several branches on the ventral aspect of the kidney. In this study, we observed that these ventral branches gave off several perpendicular smaller vessels which later run between the renal crypts (Figs. 2A–2E). These smaller vessels, as well as the ventral branches, had a distinct wall and debouched in a system of interconnected sinuses between the long epithelial crypts (Figs. 2A–2E), which were mostly perpendicular to the outer mantle surface (Fig. 2B). The epithelium of crypts was composed of microvillar cells, which also covered the afferent and efferent vessels supplying the organ (Figs. 2A and 2B).

Finally, blood from the renal sinuses converged into several large and thin-walled draining vessels (Fig. 2E) which in turn debouched in the efferent renal vein that terminated in the heart auricle (Andrews, 1965).

### Renal hemocyte islets in the normal kidney

Renal hemocyte islets were observed in all animals and were contained within large sinuses. The hemocyte islets tended to be larger towards the renal chamber (Figs. 2A and 2B). Besides that, hemocyte aggregates also occurred as rather continuous perivascular accretions on the afferent perpendicular vessels (Figs. 2A–2D).

**Figure 3:**
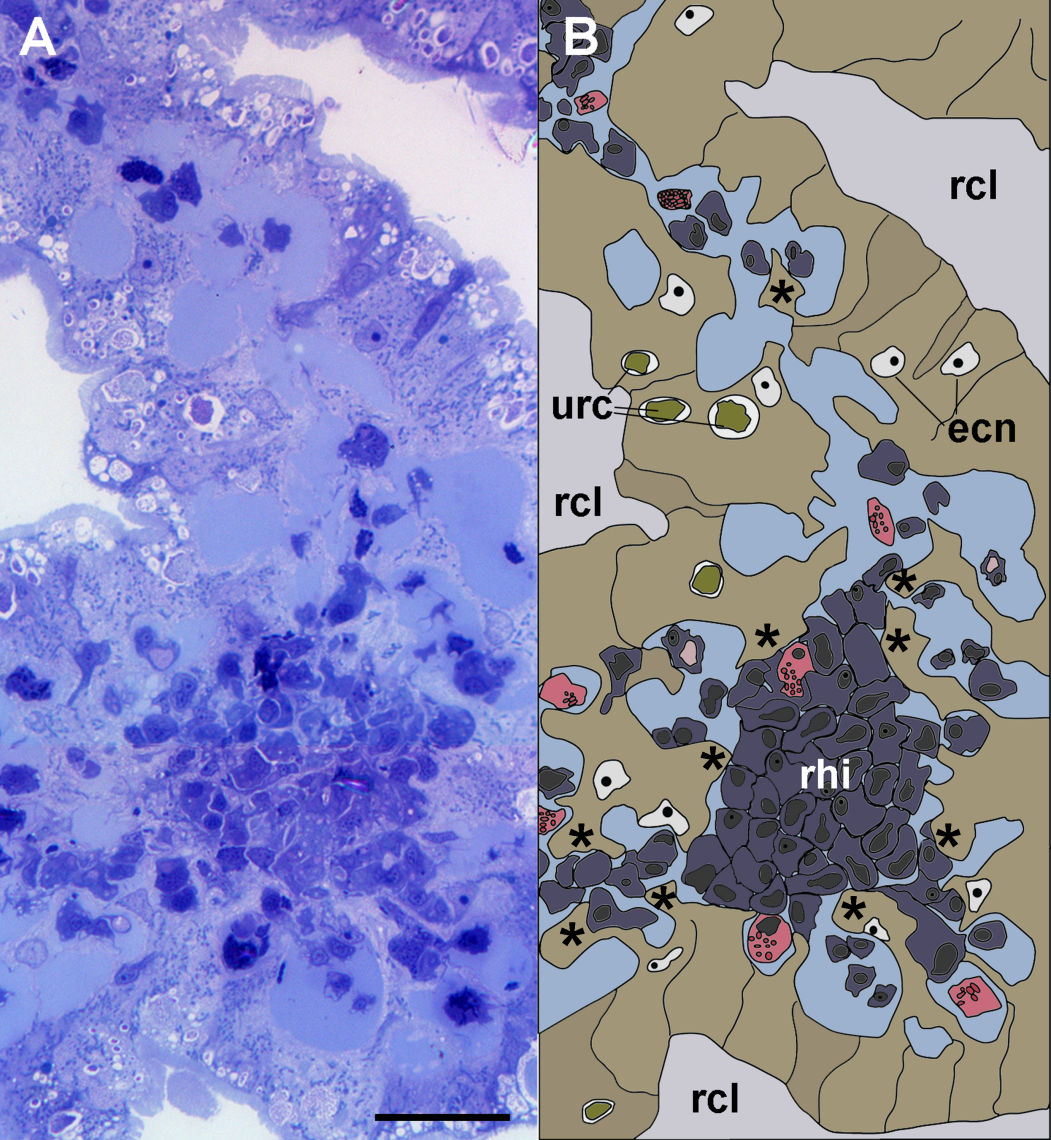
Anchoring of a renal hemocyte islet. (A) Section through a medium-sized hemocyte islet (Spurr resin embedding, toluidine blue stain). (B) Diagram of the same section shown in A, highlighting a large hemocyte islet together with smaller ones which are retained in place by cytoplasmic projections (asterisks) of urinary epithelial cells (light brown) of renal crypts; the space for the flowing blood plasma is shown in pale blue. Hyalinocytes are shown in blue while granulocytes are shown in pink. Scale bar represents 20 µm. Abbreviations: ecn, epithelial cell nuclei; rcl, renal crypt lumen; rhi, renal hemocyte islet; urc, urinary concretions.

Light microscopy of thin Spurr resin sections (Fig. 3) showed that hemocyte islets laid anchored to the renal epithelium by means of cytoplasmic projections of epithelial cells that bore numerous urinary concretions. Under light microscopy, islets appeared composed largely of hyalinocytes, which laid close to each other, with almost no extracellular spaces. Some granulocytes were seen mainly adhering to the islets’ periphery.

**Figure 4:**
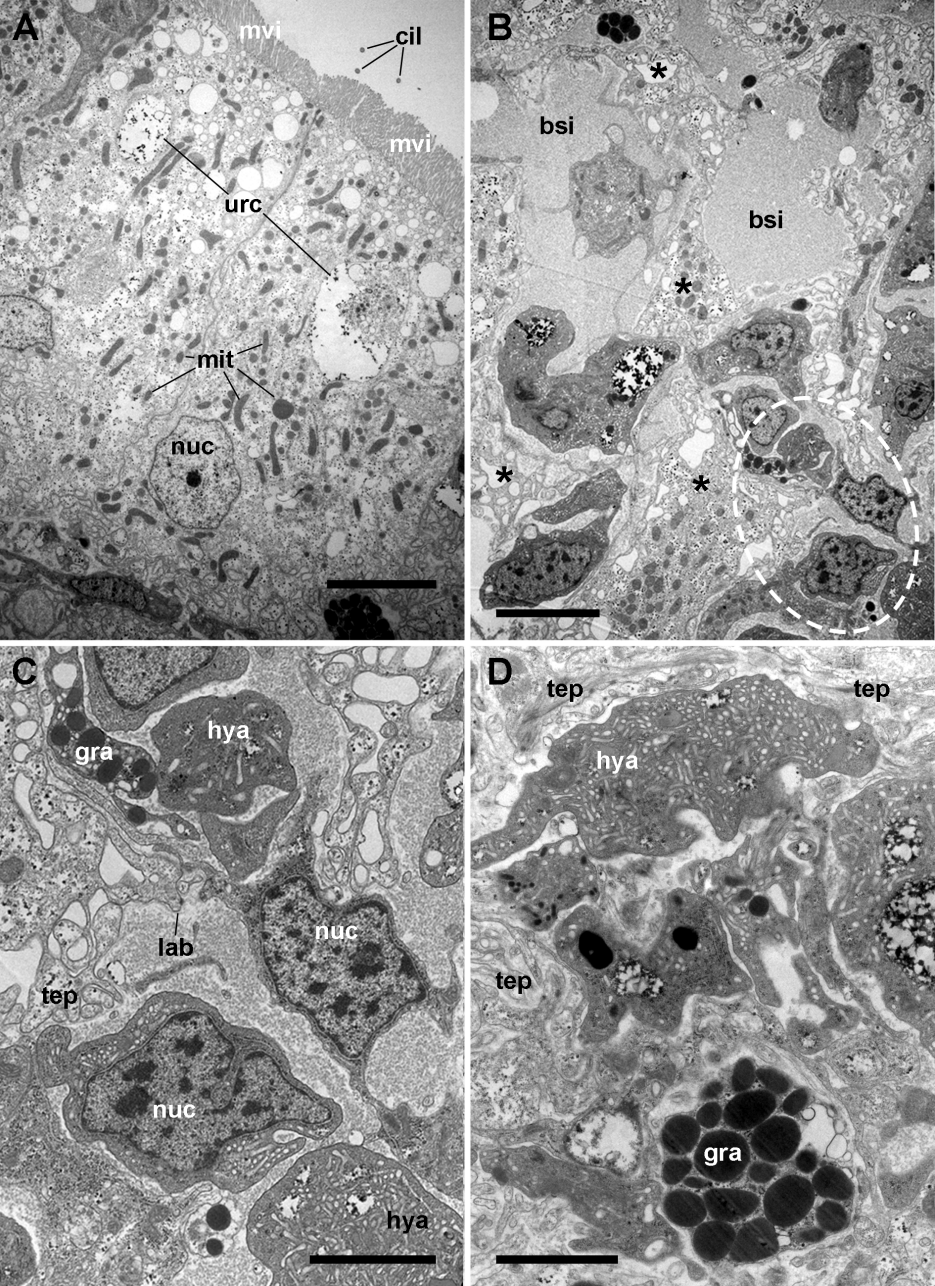
The renal epithelium and its basal projections. (A) Urinary epithelial cells showing numerous mitochondria and vacuoles containing some electron-dense particles, probably corresponding to the urinary concretions seen under light microscopy. Apical specializations include long, often ramified, microvilli, and a few cilia. (B) The basal domain extends as thick and ramified epithelial projections (asterisks) that anchor the hemocyte islets. (C) Higher magnification of the oval region outlined in (B) showing the close relationship between hemocytes and epithelial projections, which often delimit blood spaces with abundant hemocyanin particles. (D) Detail of the extensive mesh of thin epithelial projections. These cytoplasmic extensions, which arise out from the thicker ones, are in intimate contact with hemocytes and provide anchorage to the hemocyte islets. Transmission electron microscopy. Scale bars represent: (A–B) 5 µm; (C–D) 3 µm. Abbreviations: bsi, blood sinus; cil, cilium; gra, granulocyte cytoplasm; hya, hyalinocyte cytoplasm; lab, lamina basalis; mit, mitochondrion; mvi, microvilli; nuc, cell nucleus; tep, thin epithelial projections; urc, urinary concretion.

Transmission electron microscopy of renal epithelial cells (Fig. 4A) showed large and electron-lucent nuclei with conspicuous nucleoli, and a cytoplasm with numerous mitochondria and large vacuoles containing some electron-dense particles, which would correspond to the urinary concretions seen under light microscopy. Also, numerous small electron-dense clumps were free in the cytoplasm and they were present in the basal domain of these cells, where many thick cytoplasmic projections originated (Figs. 4B and 4C; also seen under light microscopy, Fig. 3). These thick cytoplasmic projections bore both mitochondria and electron-dense clumps, and gave origin to thinner cytoplasmic projections. Thin projections also showed small electron-dense clumps but no mitochondria, and they frequently ramified and laid together in bundles. Either the bundles or the individual projections formed an extensive mesh interwoven with hemocyte projections, providing a firm anchorage to the hemocyte islets (Fig. 4D). Besides that, the basal membrane of thinner epithelial projections was frequently discontinuous so that the epithelial projections resulted in direct contact with the islet’s hemocytes.

Apical specializations of epithelial cells included long microvilli and a few very long cilia, whose basal bodies were seldom seen (Fig. 4A). Desmosome-like intercellular junctions occurred only in the upper lateral domain of epithelial cells, while septate junctions occurred along the remaining part of their lateral domain.

**Figure 5:**
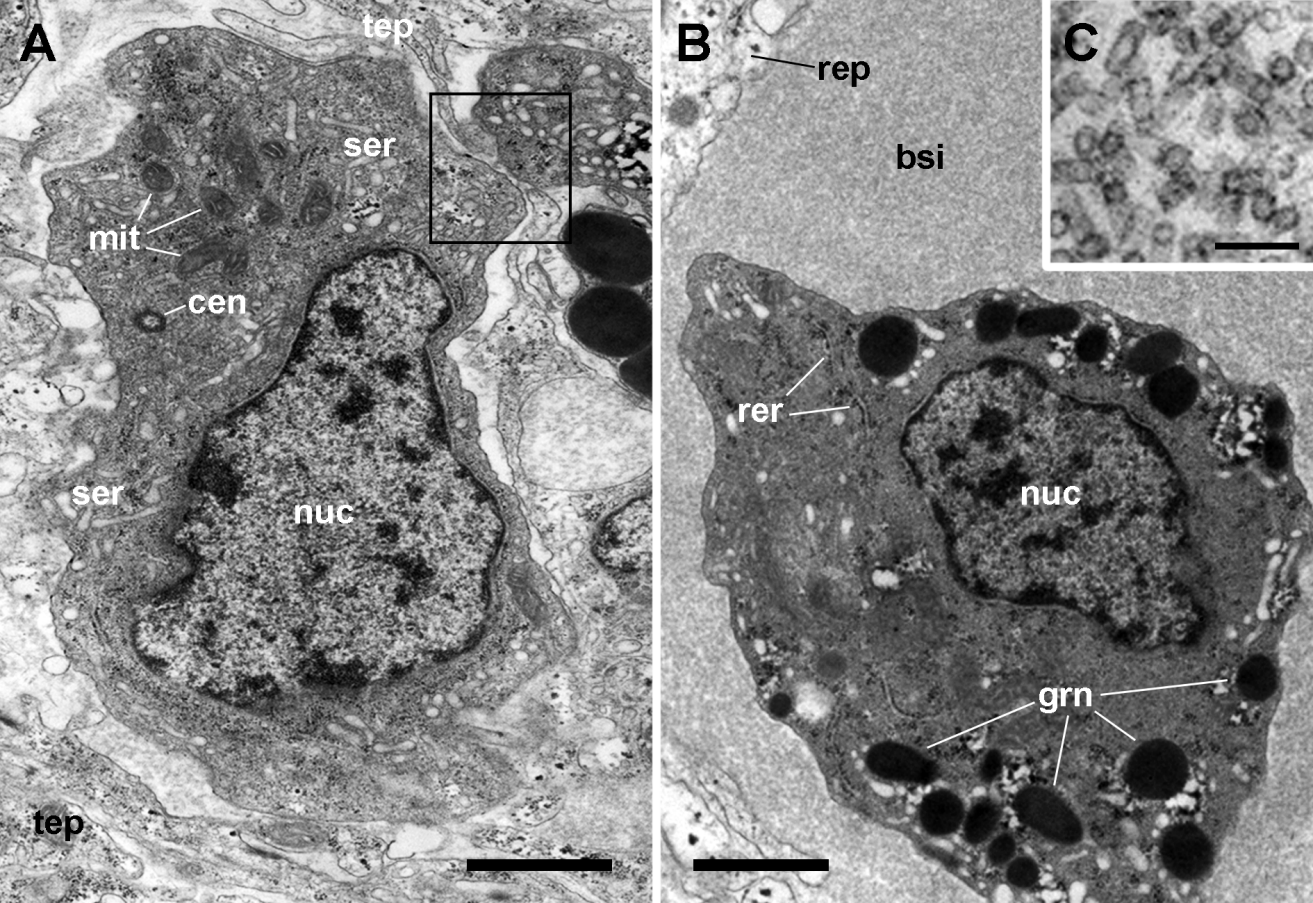
Hemocyte types and hemocyanin particles in the kidney. (A) A hyalinocyte showing its eccentric nucleus, vesicles of the smooth endoplasmic reticulum and numerous mitochondria. Intimate contacts between different hemocytes and thin epithelial projections are also seen (box). (B) A hemocyanin-filled blood sinus containing a granulocyte showing its eccentric nucleus and electron-dense R-granules. (C) Detail of hemocyanin particles. Transmission electron microscopy. Scale bars represent: (A–B) 1 µm; (C) 100 nm. Abbreviations: bsi, blood sinus; cen, centriole; grn, R-granules; mit, mitochondrion; nuc, cell nucleus; rep, renal epithelium; rer, rough endoplasmic reticulum; ser, smooth endoplasmic reticulum; tep, thin epithelial projection.

The hemocyte nuclei were much more heterochromatic than those of epithelial cells, and their cytoplasm was also darker, so that these cells and their cytoplasmic projections could be easily distinguished from those of epithelial cells. As previously reported (Cueto et al., 2015), the islets were mainly composed of hyalinocytes which were packed together and showed thin agranular cytoplasmic projections in the often narrow space that separated them (Fig. 5A). However, cytoplasmic projections containing R-granules (Cueto et al., 2015) that are typical of granulocytes, were also seen between the hyalinocytes (Figs. 4B–4D). This indicated that granulocytes within the islets were much more pleomorphic than those found in the circulation and that their projections extended towards the islets’ core. However, granulocyte perikarya were also found (Fig. 5B).

Also, small blood spaces were delimited by the epithelial projections and the hemocytes in the periphery of the islets. Not surprisingly, either loose hemocytes or hemocytes in small aggregations were sometimes found within these spaces. But an unexpected finding was the presence of abundant hemocyanin images in these spaces. Their cylindrical shape and size corresponded with the images of *P. canaliculata*’s purified hemocyanin (Fig. 5C, inset) shown by Barros, Cruz-Höfling & Matsuura (1993).

### Hemocyte reactions in renal islets after yeast injection

#### Islets enlargement and nodulation

**Figure 6:**
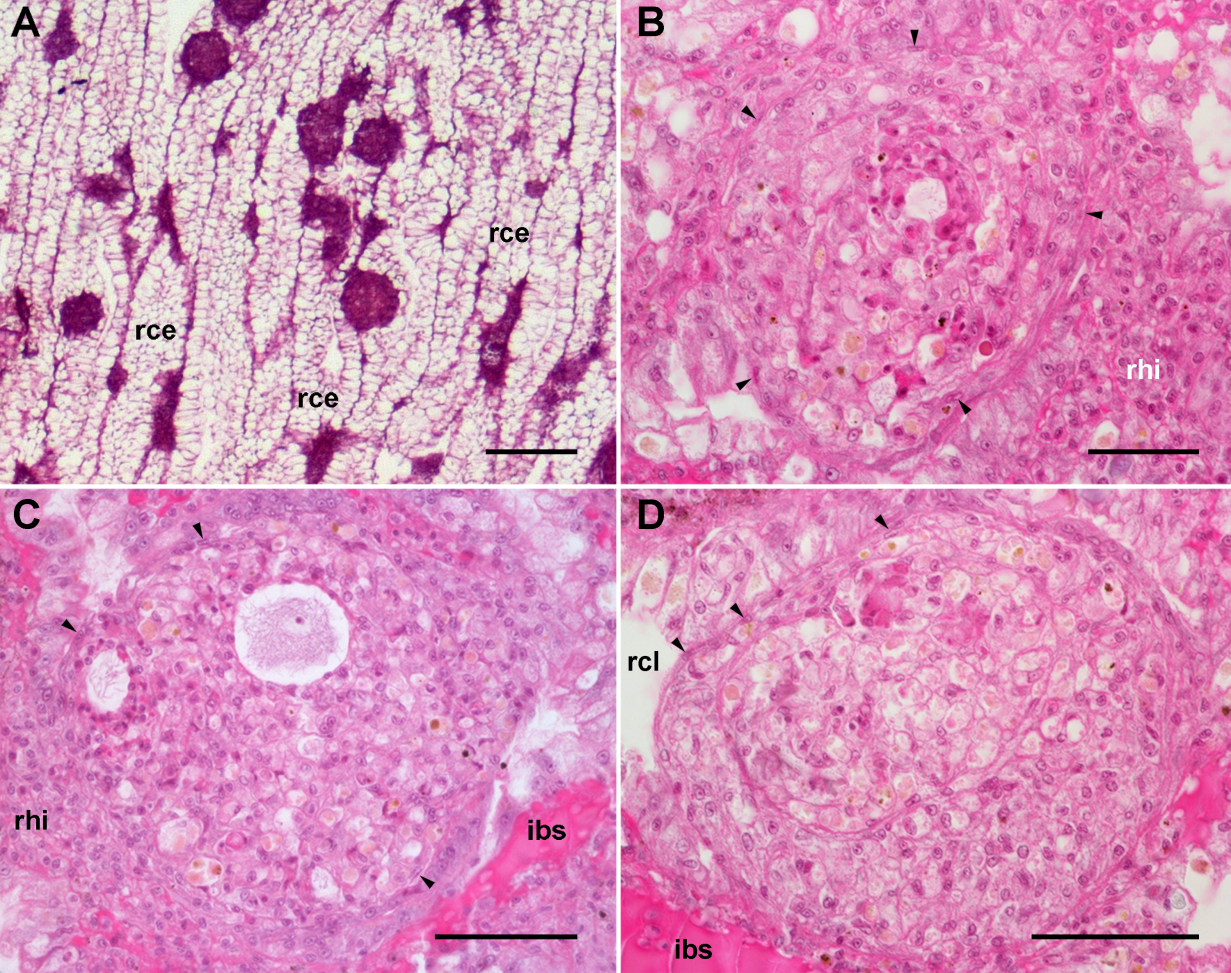
Renal hemocyte reactions after an immune challenge. (A) Field view of the kidney showing enlarged islets and nodules 96 h after yeast inoculation. (B) and (C) Detail of hemocyte nodules in another treated animal showing acellular cavities, apoptotic bodies, lipofuscin-like deposits, some granulocytes and a delimiting band of flattened cells (black arrowheads). The nodule is pushing against the cavity of a crypt in (B), while is in contact with a blood sinus in (C). (D) A nodule made of a distinct core and a cortex; the core appears to be in a more advanced stage of regression; bands of flattened cells are seen separating the core from the cortex, and the cortex from the surrounding tissues and a blood sinus. Hematoxylin-eosin. Scale bars represent: (A) 200 µm; (B–D) 50 µm. Abbreviations: ibs, intercryptal blood sinus; rce, renal cryptal epithelium; rcl, renal cryptal lumen; rhi, renal hemocyte islet.

An apparent increase in the extent of islets was observed 96 h after yeast injection, but this was not as evident in perivascular accretions. Also, spheroidal hemocyte aggregates within the islets (=nodules) were frequently seen (Fig. 6A). As the islets themselves, the nodules were composed by hyalinocytes and a much smaller number of granulocytes. No yeast particles could be identified within nodules, likely because they were already degraded 96 h after injection (it should be kept in mind that *in vivo* phagocytosis of yeast cells occurs within 2 h after yeast injection; Cueto et al., 2015). Layers of flattened cells separated the nodules from the surrounding blood sinuses and tissues. Nodules often contained extracellular lacunae, apoptotic bodies, and brown concretions that resembled lipofuscin, which indicates that a certain degree of regression was already occurring 96 h after yeast injection (Figs. 6B–6D). Similarly, evidence of cell death was observed in nodules in 72 h hemocyte cultures (Cueto, Vega & Castro-Vazquez, 2013).

In some cases, the nodules appeared formed out of two or more foci, which may have been caused by successive waves of cellular aggregation, sometimes resulting in a core showing a more advanced stage of regression, and a cortex made by recently aggregated cells (Fig. 6D). Sometimes, the growth of these nodules obstructed the blood sinuses that contained them, and they also grew and protruded into the crypt’s cavity and thus into the renal chamber (Fig. 6D).

#### Quantitative changes in hemocyte proliferation

Measurements were made in two ways: (1) by a BrdU incorporation assay to measure cell proliferation in the islets (including nodules), both at 48 and 96 h after injections (either vehicle or yeast injection), and (2) by measuring the renal area occupied by hemocyte aggregates (aggregates/total renal tissue ratio), only 96 h after injections (because nodulation did not constantly appear at 48 h).

**Figure 7:**
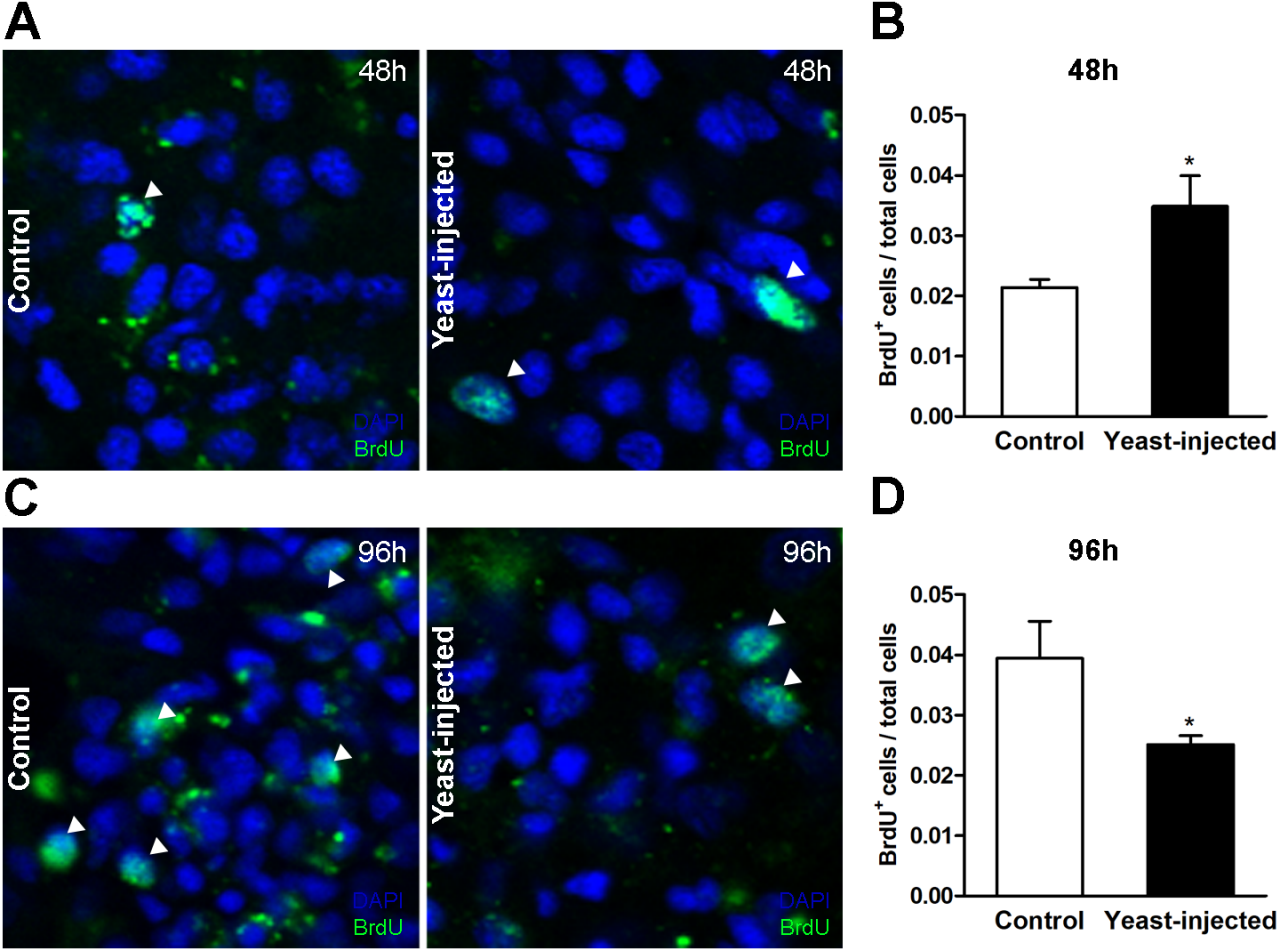
Quantification of hemocyte proliferation in renal islets. (A) Indirect immunofluorescence detection of BrdU^+^ cells on renal hemocyte islets 48 h after the injection of either vehicle or yeast cells. (B) Proliferation (BrdU^+^cells either per total number of cells or per area) was higher in yeast-injected than in control animals, 48 h post-injections. (C) Indirect immunofluorescence detection of BrdU^+^ cells on renal hemocyte islets 96 h after the injections. (D) Proliferation was lower in yeast-injected than in control animals, 96 h post-injections. Six images per animal were analyzed. Results are expressed as mean ± SEM; asterisks indicate significantly different from control; NS indicate non-significantly different (one-tailed Mann–Whitney *U*-test).

Forty-eight hours after injections, the number of BrdU^+^ hemocyte nuclei / total hemocyte nuclei in the islet section showed a statistically significant 2-fold increase in yeast-injected animals, as compared to controls (Figs. 7A and 7B). In contrast, 96 h after injection, the mean value of this proportion was lower in yeast-injected than in control animals (Figs. 7C and 7D).

The ratio ‘hemocyte aggregates area/total renal tissue area’ was higher in treated than in control animals, 0.26 ± 0.03 *vs*. 0.19 ± 0.02, respectively, at the end of the experimental period, i.e., 96 h after yeast or vehicle injection; the difference was statistically significant. In a preliminary experiment (three control animals and three yeast-injected animals) no nodules were observed 48 h after injection. Therefore, comparative observations were not made at this time point.

### Blood circulation in the normal lung and the ‘respiratory lamina’

The lung is a flattened sac extending over most of the mantle cavity. It is delimited by the gill on its right and posterior borders, and the mantle skirt on the anterior and left borders (Fig. 1).

**Figure 8:**
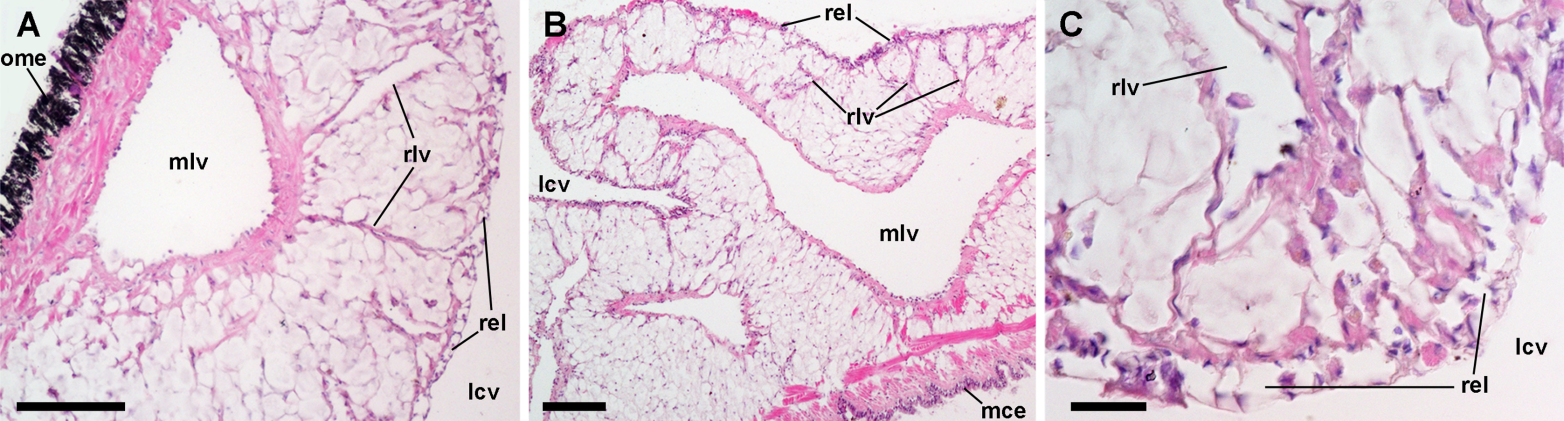
Lung microcirculation. (A) Main vessels and their radial branches in the lung roof. (B) Main vessels and their radial branches in the lung floor. (C) Detail of the respiratory lamina. Hematoxylin-eosin. Scale bars represent: (A) and (B) 100 µm; (C) 20 µm. Abbreviations: lcv, lung cavity; mce, mantle cavity epithelium; mlv, main lung vessel; ome, outer mantle epithelium; rel, respiratory lamina; rlv, radial lung vessel.

Both the roof and the floor of the lung are crossed by main vessels that convey blood from the visceral mass and the gill (Andrews, 1965). We also observed that smaller vessels arise radially from these main vessels (Figs. 8A and 8B) and terminate in numerous small chambers in contact with air, constituting the ‘respiratory lamina’ (Fig. 8C). This lamina seems to be the structure allowing gas exchange, but it may also retain microorganisms and hemocyte aggregates (see next section).

### Hemocyte reactions in the lung after yeast injection

**Figure 9:**
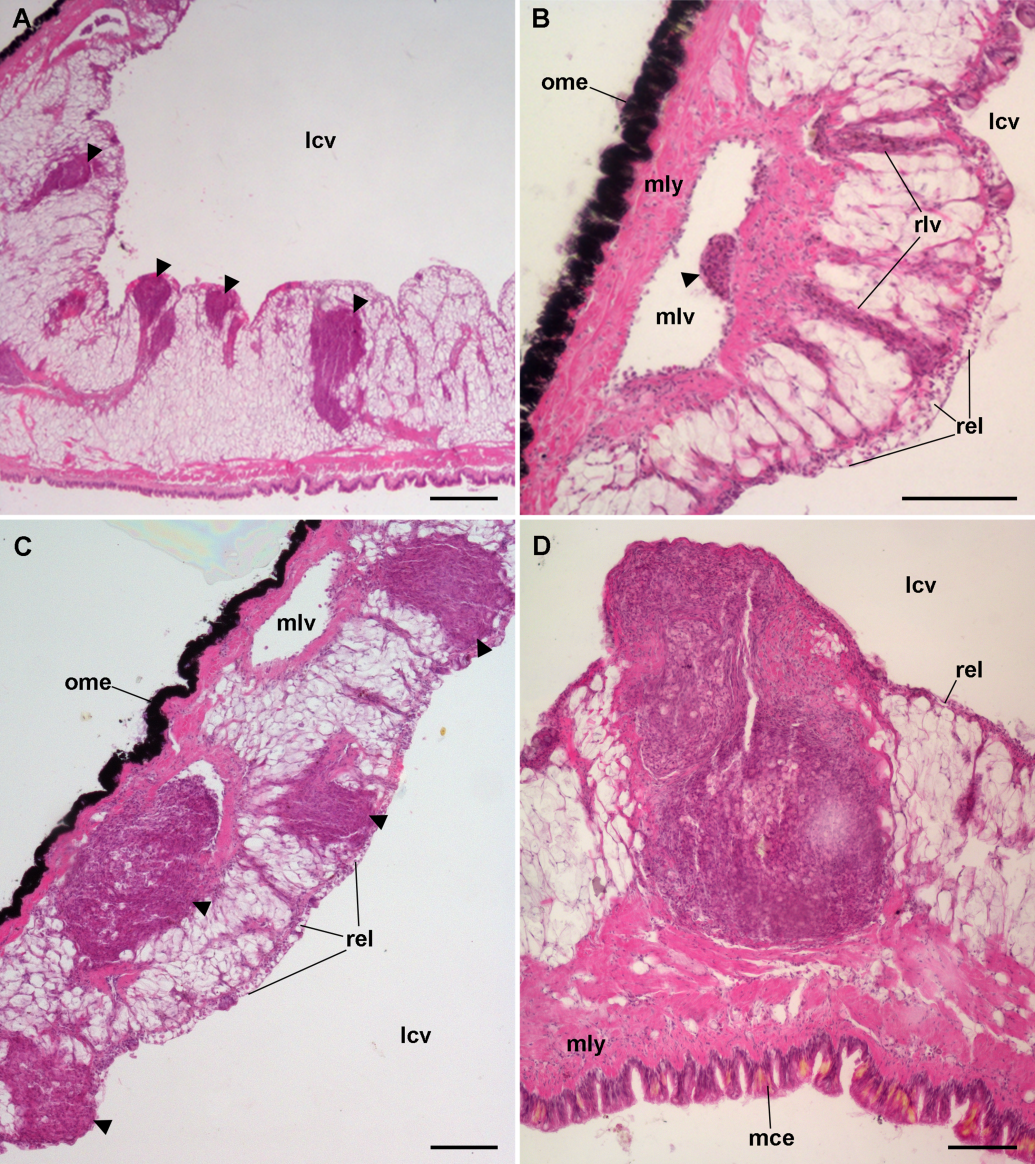
Lung hemocyte reactions after an immune challenge. (A) Several hemocyte nodules near the roof-floor junction. (B) Incipient hemocyte aggregations within a main vessel and its radial branches in the lung roof. (C) Nodules within main and radial vessels in the lung roof. (D) A large nodule in the floor protruding into the lung cavity. The hemocyte aggregates of different sizes appear to be formed initially in the main vessels and to extend later within radial vessels towards the lung cavity. Hematoxylin-eosin. Scale bars represent: (A) 250 µm; (B–D), 100 µm. Black triangles indicate nodules. Abbreviations: lcv, lung cavity; mce, mantle cavity epithelium; mlv, main lung vessel; mly, muscle layer; ome, outer mantle epithelium; rlv, radial lung vessel; rel, respiratory lamina.

The lung did not show permanent hemocyte aggregations as those of the kidney (Fig. 8), though intravascular and perivascular hemocyte aggregates and nodules were formed in treated animals (Fig. 9), and they extended towards the perivascular urate tissue described by Giraud-Billoud et al. (2008). Sometimes, hemocyte aggregation began within the main lung vessels or their radial branches (Fig. 9B), where they could grow towards the respiratory lamina and protrude towards the lung cavity or the surrounding urate tissue of the lung roof (Fig. 9C) and floor (Figs. 9A and 9D).

Hemocyte recruitment towards the blood spaces of the respiratory lamina was also observed (though this was not quantified). Lung nodules, like those in the kidney, were mainly composed of hyalinocytes. They also contained extracellular lacunae, apoptotic bodies, and brown lipofuscin-like concretions, and were sometimes delimited by a layer of flattened cells. Nodules grew out to large sizes, could be multicentered, and were many times visible to the naked eye (Fig. 9D). No nodule formation was observed in the gill.

## Discussion

### Blood circulation in kidney and lung and their roles as immune barriers

Recently Little, Hultmark & Read (2005) have warned against the limits of a reductionist approach to invertebrate immunology, which is currently largely if not wholly concerned with identifying the molecules, cells and functional cascades, and they pointed that this search for molecules runs the risk of missing important phenomena. This may be particularly true for a lesser known, non-model species such as *P. canaliculata*, whose anatomical peculiarities need first to be disclosed to define the way this gastropod can react to immune challenges.

This is also certainly the case of the circulatory system of this gastropod and other ampullariids, whose general organization has been studied by Andrews (1965). In general, the blood coming from the head and foot first passes through the kidney, while all blood coming from the visceral mass and the mantle cavity first passes through the lung and partly through the gill, before reaching the heart auricle through the efferent renal and pulmonary/ctenidial veins, respectively. Because of these positions in the circulation, the kidney and lung were investigated here as organ barriers preventing the spread of intruders. Additionally, the kidney was noteworthy because of its hemocyte islets, which participate in alien recognition and phagocytosis and which are likely candidates to have a role in hematopoiesis (Cueto et al., 2015).

The kidney showed an extensive vascular bed supplied by the ‘perpendicular vessels’ originated from branches of the greater afferent renal vessel. The hemocyte accretions around the afferent perpendicular vessels were also in contact with the system of blood sinuses between the epithelial crypts (Fig. 2) which contained the conspicuous hemocyte islets and which were anchored by cytoplasmic projections of the crypts’ epithelial cells (Figs. 3–5). In this way, the kidney offers an extended, low-pressure bed where blood flows slowly and in which the potential intruders would be in close contact with the surface of both hemocyte islets anchored within the sinuses and the hemocyte accretions around the perpendicular afferent vessels. It should be emphasized that the elaborate array of renal epithelial projections—and their direct contact with the islet’s hemocytes—suggests a close functional relationship between them. In addition, the occurrence of abundant hemocyanin particles in the blood spaces suggests some functional relationship of this large protein with renal epithelial cells and hemocytes. Beyond the role of hemocyanin in oxygen transport, this notably large protein (7.6 × 10^3^ kDa; Barros, Cruz-Höfling & Matsuura, 1993) and its derived peptides are known to serve various roles in innate immunity.

In turn, the lung vasculature is composed of the main vessels conveying blood either directly from the viscera or indirectly through the gill vasculature (Andrews, 1965). The current study showed that blood in the main vessels was conducted through the smaller radial vessels to a continuous lamina of small blood chambers (the respiratory lamina), which lined the lung cavity (Fig. 8). In this way, the lung would provide another ample bed where intruders conveyed in the slowly flowing blood would become in close contact with hemocytes.

Therefore, both the kidney and the lung may serve a role analogous to that of lymph nodes in vertebrates, where foreign particles and organisms circulating in the lymph can be trapped and inactivated (Pawlina, 2017). Curiously, the gill did not show nodulation after yeast injection. However, this organ may also serve as an immune barrier, but one in which intraepithelial granulocytes played the most significant role (C Rodriguez, GI Prieto, IA Vega & A Castro-Vazquez, 2018, unpublished data).

### Nodulation in the immune barriers

A notable feature of the kidney and lung was to host spheroidal hemocyte aggregates or ‘nodules’ which were formed in response to an immune challenge (Figs. 6 and 9). Satyavathi, Minz & Nagaraju (2014) defined ‘nodulation’ as a process involving the entrapment of invading microorganisms by the aggregation of hemocytes around them. Nodulation involves phagocytic hemocytes and may be quantitatively the most important defense mechanism against bacterial and fungal infections in invertebrates (Miller, Nguyen & Stanley-Samuelson, 1994).

In the kidney of *P. canaliculata*, nodules were formed on the surface of hemocyte islets and as outgrowths of the perivascular hemocyte accretions (Fig. 6). In the lung of this gastropod, however, hemocyte aggregates did not occur as permanent structures, but they were readily formed within the main supplying vessels after yeast injection. These aggregates extended into the radial vessels and/or probably got stuck into them or in the respiratory lamina (Fig. 9). In fact, these nodules shared structural similarity with those formed *in vitro* by hemocytes of this species, i.e., they showed compact core/s, zones with extracellular lacunae and an outer zone of more flattened cells (Cueto, Vega & Castro-Vazquez, 2013). In that study, a variable proportion of dying cells was observed within nodules even in axenic hemocyte cultures.

It is interesting that similar nodulation has also been observed in other molluscs (e.g., Abdel-Aziz, 2009; Nacif-Pimenta et al., 2012; Pauley & Krassner, 1972; Penagos-Tabares et al., 2018; Van der Knaap, Adema & Sminia, 1993) as well as in other taxa representing the major Metazoan clades, such as cnidarians (e.g., Robb et al., 2014), crustaceans (e.g., Burnett & Burnett, 2015; Cerenius et al., 2010; Martin et al., 1996; Martin et al., 1993), insects (e.g., Duressa, Vanlaer & Huybrechts, 2015; Satyavathi, Minz & Nagaraju, 2014), and echinoderms (e.g., Canicattì & D’Ancona, 1989). Even chordates could be included here if granulomatous reactions were considered a particular form of nodulation in which non-phagocytic blood cells, such as lymphocytes, may also participate (Davis & Ramakrishnan, 2009, e.g., Swaim et al., 2006). Also, pigment deposition was frequently found in these nodules (Swaim et al., 2006; Valembois, Seymour & Lassègues, 1994) and they may be either lipofuscin (as a consequence of lipoprotein peroxidation, e.g., Canicattì, D’Ancona & Farina-Lipari, 1989) or melanin (as a consequence of the phenoloxidase cascade, e.g., Satyavathi, Minz & Nagaraju, 2014).

The seemingly universal distribution of nodulation in the Metazoa almost parallels that of phagocytosis, which is certainly the most basal and evolutionary conserved defense mechanism in animals (see Cueto, Vega & Castro-Vazquez, 2013, for *P. canaliculata*).

### Proposed hematopoietic sites in gastropods

Gastropod hematopoiesis and its controlling factors have been reviewed recently (Pila et al., 2016). In general, hematopoiesis may be localized to specific tissues/organs (e.g., Salamat & Sullivan, 2008; Sullivan, Cheng & Howland, 1984) or may be widespread in the connective tissue and/or the general circulation (e.g., Sminia, 1974). Research on localized hematopoiesis has been practically limited to six families of Panpulmonata (Heterobranchia), namely, Planorbidae (e.g., Jeong, Lie & Heyneman, 1983; Lie, Heyneman & Yau, 1975; Nacif-Pimenta et al., 2012; Pan, 1958; Pan, 1965; Richards, 1975; Sullivan, Pikios & Alonzo, 2004), Bulinidae ( Kinoti, 1971), Lymnaeidae (e.g., Rondelaud & Barthe, 1981; Ruellan & Rondelaud, 1992), Physidae (Sullivan, 1988), Bradybaenidae, and Strophocheilidae (Rohr & Amato, 2014). For these families, hematopoietic sites have been proposed in a region lying between the mantle cavity, the saccular kidney and the pericardium. In all these cases, these proposed sites were supplied by blood sinuses and/or ‘spaces’ which warranted the passage of the newly formed hemocytes to the systemic blood circulation.

However, there is a paucity of data in other gastropod clades, even in the Caenogastropoda, which encompasses 60% of species in the widely diverse class Gastropoda. Studies in caenogastropods are restricted to two species of Ampullariidae, *P. canaliculata* and *Marisa cornuarietis*. In a previous report, Accorsi, Ottaviani & Malagoli (2014) proposed a pericardial hematopoietic district in *P. canaliculata*. At first glance, this could suggest a site homologous to those reported in the Panpulmonata. However, these authors only reported dividing cells in the epicardial membrane and in the fluid contained within the pericardial coelom, which does not warrant a direct passage to the hemocoel, because the pericardial coelom rather connects with the renal chamber through the renopericardial canal, i.e., with urine and not with blood. Also, these authors did not report any quantitative response of dividing cells to the stimulus used (repeated blood withdrawals).

Other tissues/organs, namely the lung roof of *M. cornuarietis* (Yousif, Blähser & Lämmler, 1980) and the renal islets of *P. canaliculata* (Cueto et al., 2015) have been proposed as possible hematopoietic sites. Yousif, Blähser & Lämmler (1980) reported a band of tissue in the lung roof, which thickens and forms nodules in response to the experimental infection with the nematode *A. cantonensis*, while Cueto et al. (2015) proposed that the permanent hemocyte aggregations in renal islets would be a site of hematopoiesis*.*

Because of the proposed hematopoietic site in the lung of *M. cornuarietis* (Yousif, Blähser & Lämmler, 1980), we have searched for a similar site in *P. canaliculata* (C Rodriguez, GI Prieto, IA Vega & A Castro-Vazquez, 2018, unpublished data). In that study, we serially sectioned the lung of this species but did not find any similar tissue band, which might be peculiar to *M. cornuarietis*.

In the current study, we have confirmed that the renal hemocyte islets are permanent and rather compact aggregations (Cueto et al., 2015). Besides that, we showed a close association of the hemocyte islets with a mesh of cytoplasmic projections of renal epithelial cells, with a frequently discontinuous basal membrane, and which anchored the islets within the efferent blood sinuses. Also, a striking abundance of hemocyanin particles was found in blood spaces in the periphery of the islets, suggesting some role of the kidney in the metabolism and/or function of this notably large protein (7.6 × 10^3^ kDa; Barros, Cruz-Höfling & Matsuura, 1993) which has diverse immune roles (Coates & Nairn, 2014).

Furthermore, changes in hemocyte proliferation in renal islets after yeast injection were measured by determining the increase in BrdU incorporation in hemocyte nuclei and the increase in the area occupied by hemocytes at the end of the experiment. Statistically significant increases in BrdU incorporation in hemocytes were shown 48 h after challenge (Fig. 7B) and in the islet’s area 96 h after challenge. Also, BrdU incorporation in hemocytes in the control group indicated that a rather high basal proliferation rate was sustained in the islets. The decrease in BrdU positive nuclei observed 96 h after yeast injection (Fig. 7D) may be explained by a left shift of the proliferation curve, or to an increase in cell death.

However, future research should further refine these findings by considering the possible inward and outward hemocyte movements, to and from the islets, as well as the accumulation of newly formed hemocytes. These variables have been studied for the hematopoietic organ of crayfish (Söderhäll, 2016; Söderhäll et al., 2003). However, no similar studies have yet been made in gastropods (Pila et al., 2016). To our knowledge, the current report is the first quantitative approach to hemocyte proliferation in a caenogastropod and points to the renal islets as a potentially significant hematopoietic site in *P. canaliculata*, either by resident or migrating cells.

Furthermore, we showed that nodules also formed in the lung of *P. canaliculata* after challenge, an organ in which preexisting hemocyte aggregates seldom occur and they are likely responses to some occasional infection. Nodules are likely formed by the locally proliferating hemocytes, but also by hemocytes attracted to particular places (Figs. 6 and 9), and these nodules may also represent non-permanent sites of hematopoiesis. Whether hematopoiesis within these nodules is only directed to eliminate the intruder that elicited the reaction or, alternatively, they contribute to circulating hemocyte levels, would have to be determined.

Besides this evidence of localized hematopoiesis in the Ampullariidae, it is worth mentioning there is evidence of hematopoietic progenitor cells in the circulation of *P. canaliculata* (IA Vega, C Rodriguez, V Simon & P Conget, 2018, unpublished data). Therefore, it is possible that both widespread and localized hematopoiesis occur in this species.

## Conclusions

Here the intricate blood circulation in the kidney and lung of *P. canaliculata* was unraveled, i.e., where hemocytes may become in close contact with intruders. Moreover, striking ultrastructural features of the renal hemocyte islets’ architecture have been disclosed, altogether with the unexpected finding of abundant hemocyanin particles into renal blood sinuses. Also, nodular aggregations of hemocytes were shown in both organs as a conspicuous reaction to the immune challenge. And finally, hemocyte proliferation in the renal islets has been quantified following the immune challenge. According to this evidence, the renal hemocyte islets emerge as possibly important, and perhaps the only permanent hematopoietic sites in *P. canaliculata.*

##  Supplemental Information

10.7717/peerj.5789/supp-1 Figure S1 Anatomical references for injections and sampling (A) Injections of yeast suspension or buffer in the visceral hemocoel were made through a small hole (∼1 mm) in the shell (red dot), drilled manually at midway between sutures of the second whorl after inducing body withdrawal by gently handling the animal. No exsanguination was induced (Sakharov & Rózsa, 1989) . The tip of the injecting needle was introduced ∼4 mm below the shell surface, in the digestive gland close to the testis. Neither spillage of the injected material nor further retraction of the columellar muscle was observed; thus, the whole procedure did not appear to be painful for the animals. (B) Dorsal view of the animal showing shaded areas corresponding to the samples obtained from the kidney and lung. Abbreviations: ctn, ctenidium; kid, kidney; lng, lung; opc, operculum; pod, foot; prc, pericardium; shl, shell; urt, ureter; whl, second whorl of the shell.

10.7717/peerj.5789/supp-2 Data S1 Raw data of the BrdU experiment

## Additional Information and Declarations

### Data Availability

The following information was supplied regarding data availability: The measurements of each case in BrdU experiment are available in Data S1.
