# Supplementary figures and images for: Assessment of the kidney and lung as immune barriers and hematopoietic sites in the invasive apple snail Pomacea canaliculata (part 2 of 2)
Source: PeerJ. 2018 Oct 12;6:e5789. doi: 10.7717/peerj.5789 (PMC6187997; doi:10.7717/peerj.5789)

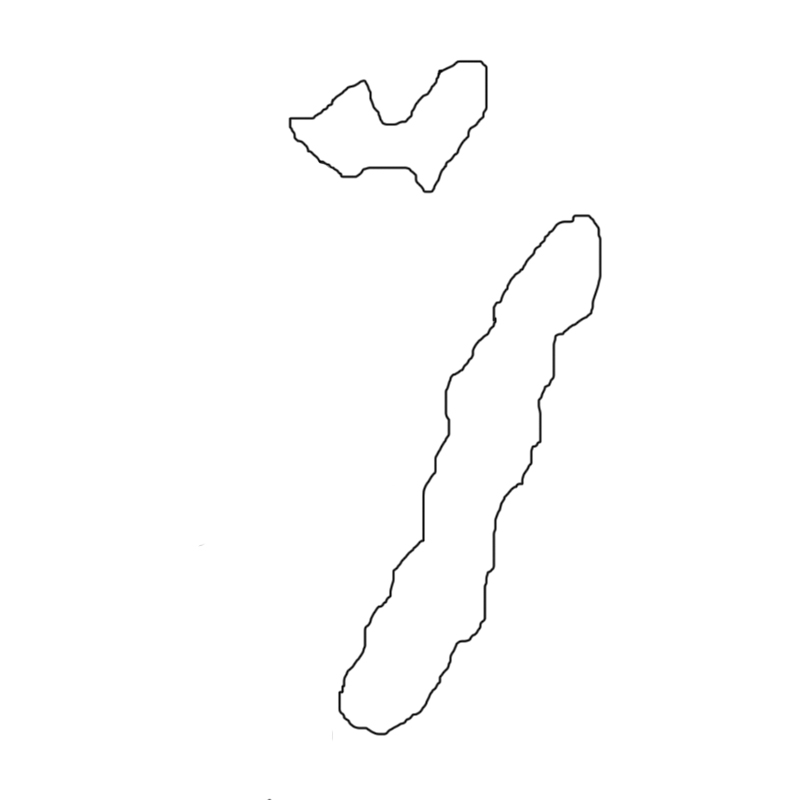

Supplement: Data S1 [file peerj-06-5789-s002.zip › C6/Image0004 GRID.jpg]

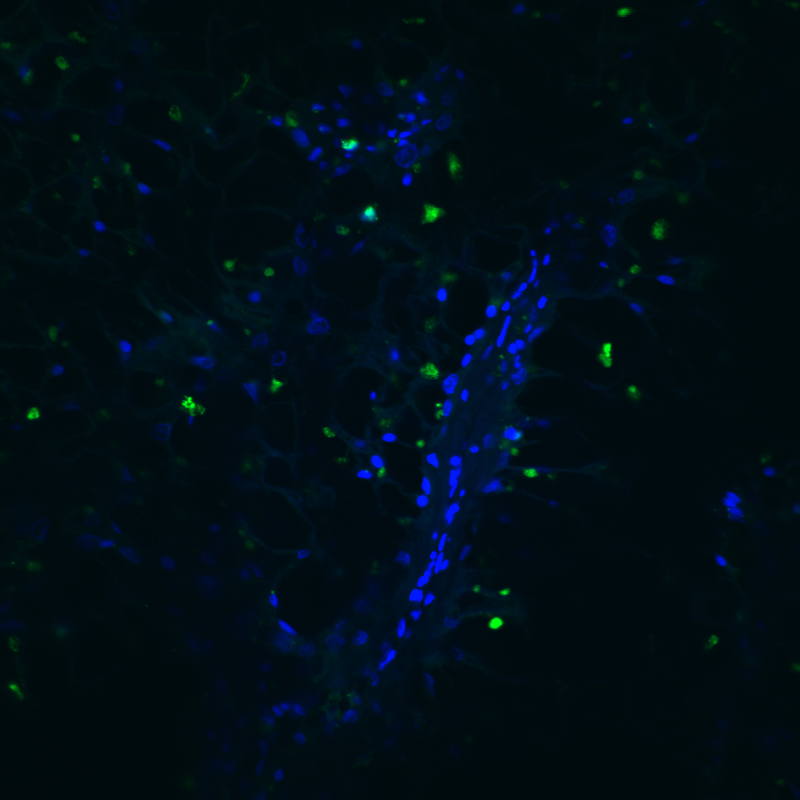

Supplement: Data S1 [file peerj-06-5789-s002.zip › C6/Image0004.jpg]

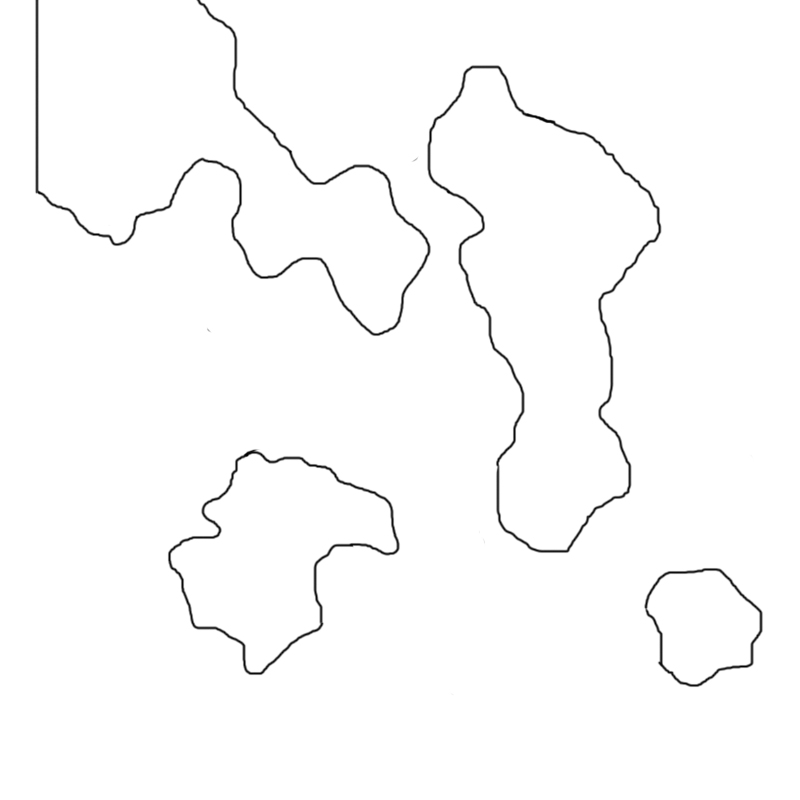

Supplement: Data S1 [file peerj-06-5789-s002.zip › C6/Image0005 GRID.jpg]

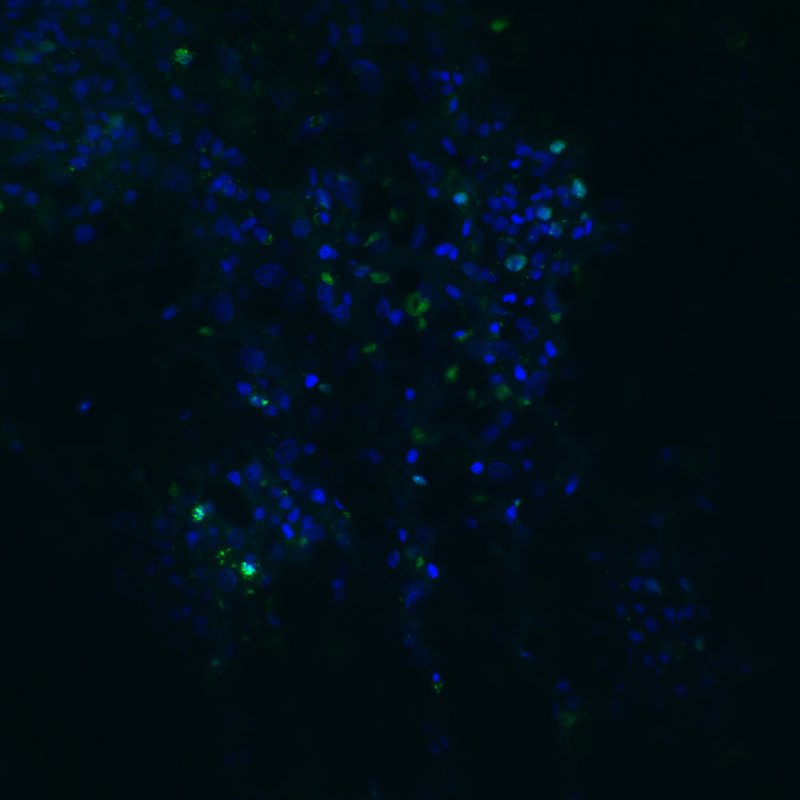

Supplement: Data S1 [file peerj-06-5789-s002.zip › C6/Image0005.jpg]

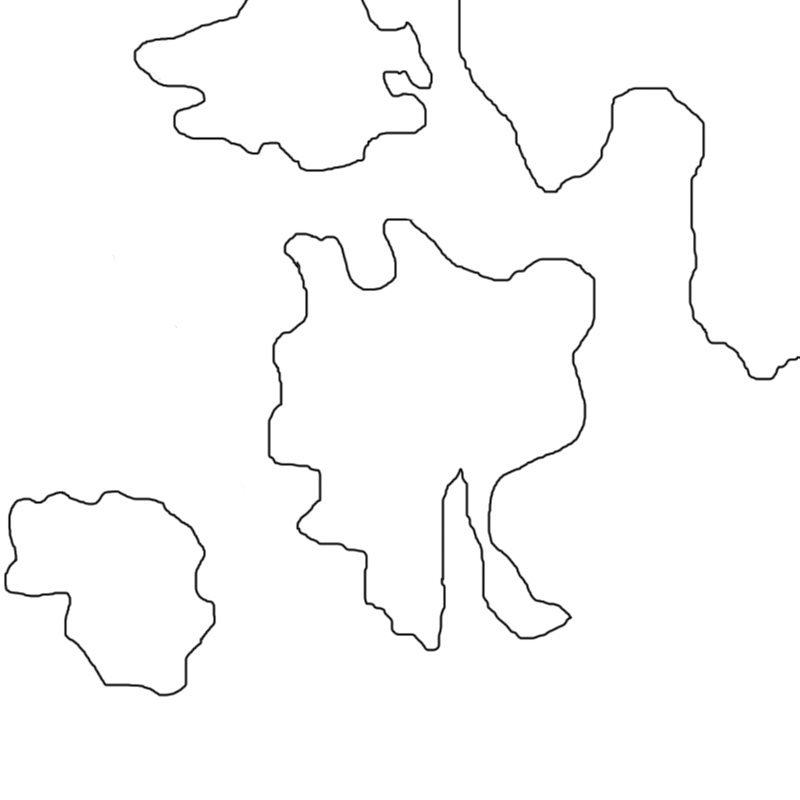

Supplement: Data S1 [file peerj-06-5789-s002.zip › C6/Image0006 GRID.jpg]

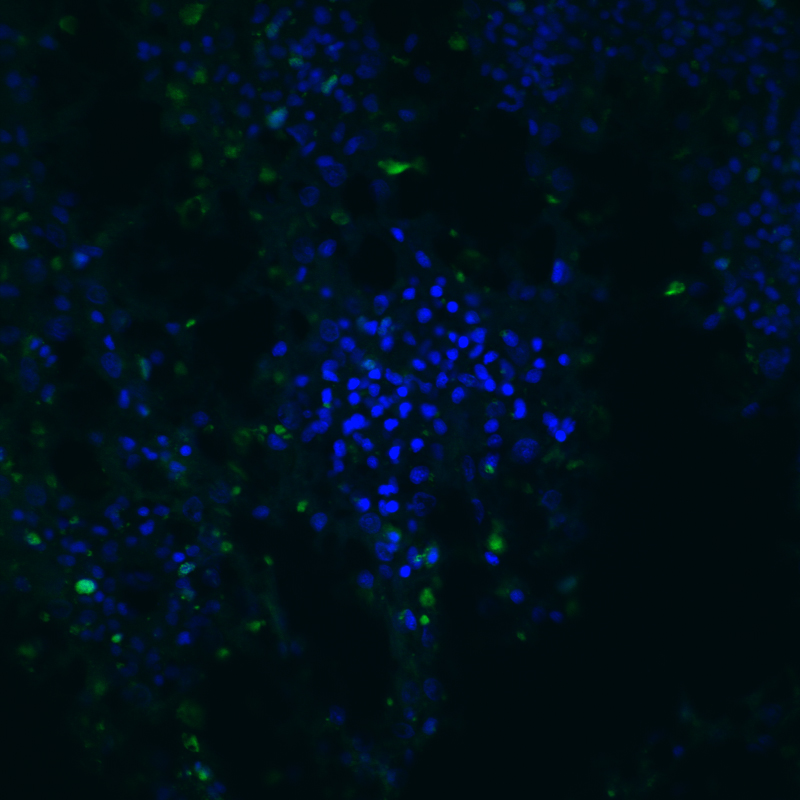

Supplement: Data S1 [file peerj-06-5789-s002.zip › C6/Image0006.jpg]

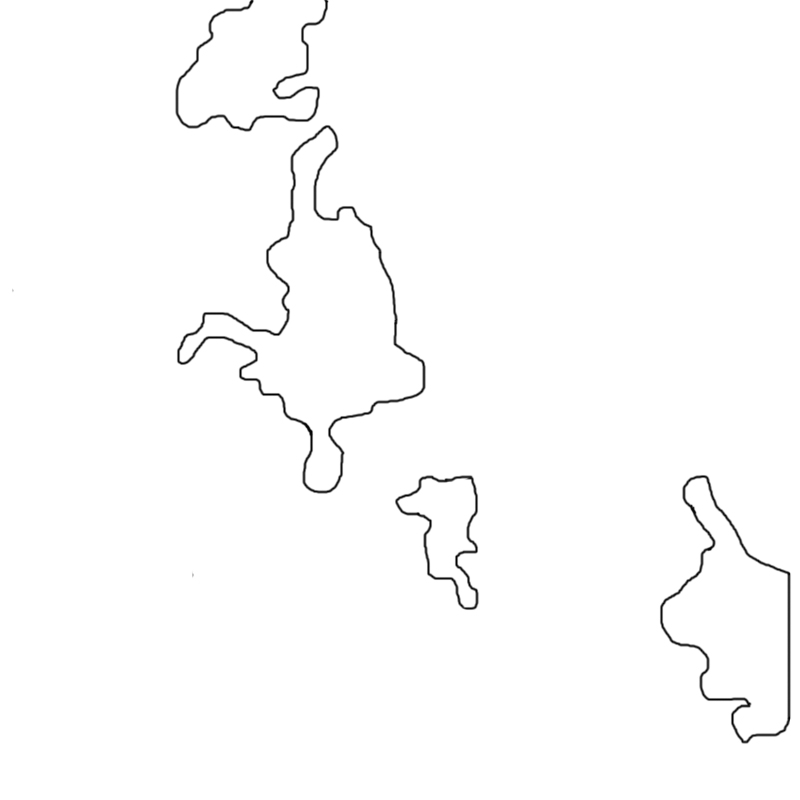

Supplement: Data S1 [file peerj-06-5789-s002.zip › C6/Image0007 GRID.jpg]

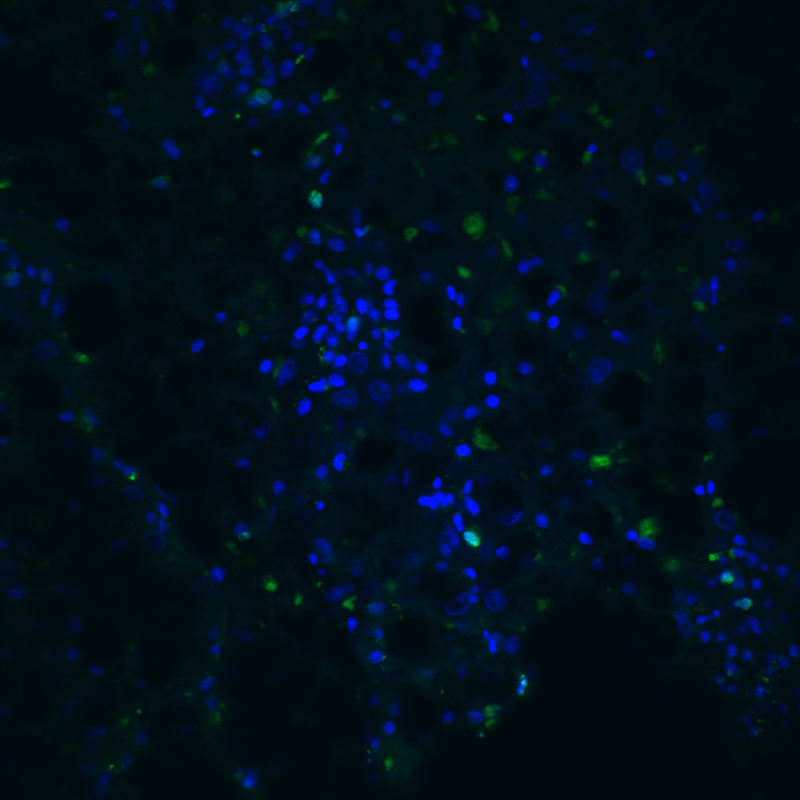

Supplement: Data S1 [file peerj-06-5789-s002.zip › C6/Image0007.jpg]

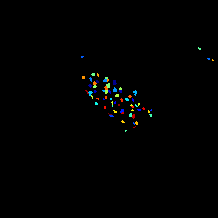

Supplement: Data S1 [file peerj-06-5789-s002.zip › T1/Im05 Nuclei.png]

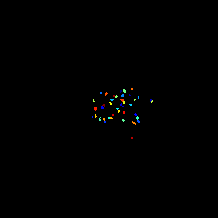

Supplement: Data S1 [file peerj-06-5789-s002.zip › T1/Im07 Nuclei.png]

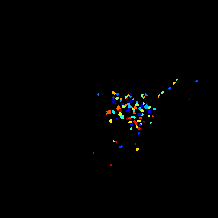

Supplement: Data S1 [file peerj-06-5789-s002.zip › T1/Im08 Nuclei.png]

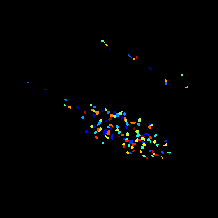

Supplement: Data S1 [file peerj-06-5789-s002.zip › T1/Im09 Nuclei.png]

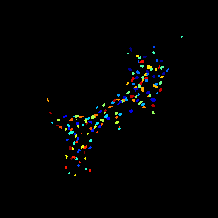

Supplement: Data S1 [file peerj-06-5789-s002.zip › T1/Im10 Nuclei.png]

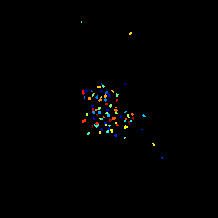

Supplement: Data S1 [file peerj-06-5789-s002.zip › T1/Im16 Nuclei.png]

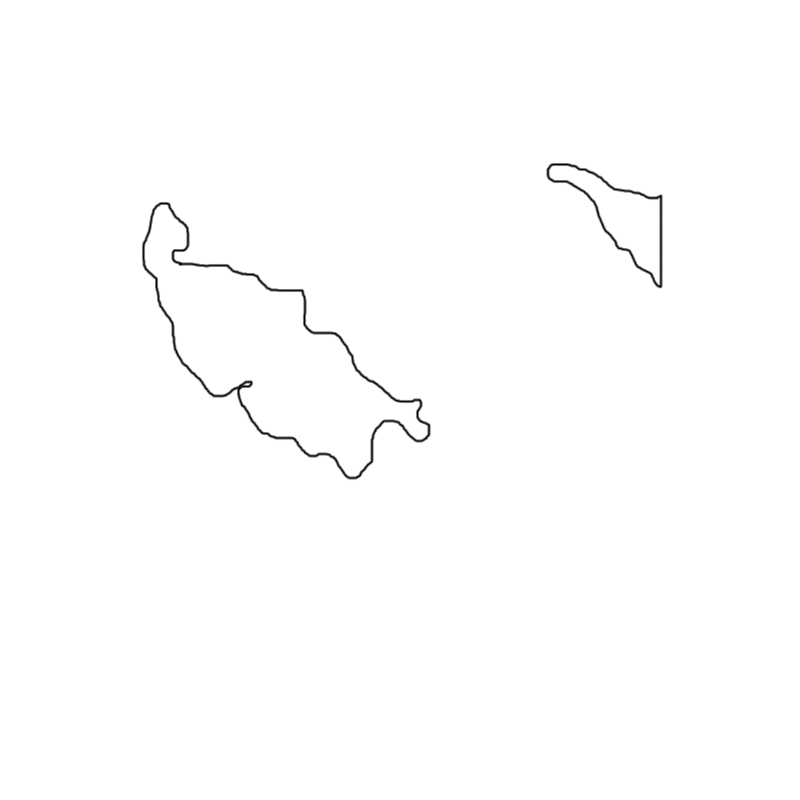

Supplement: Data S1 [file peerj-06-5789-s002.zip › T1/Image0005 GRID.jpg]

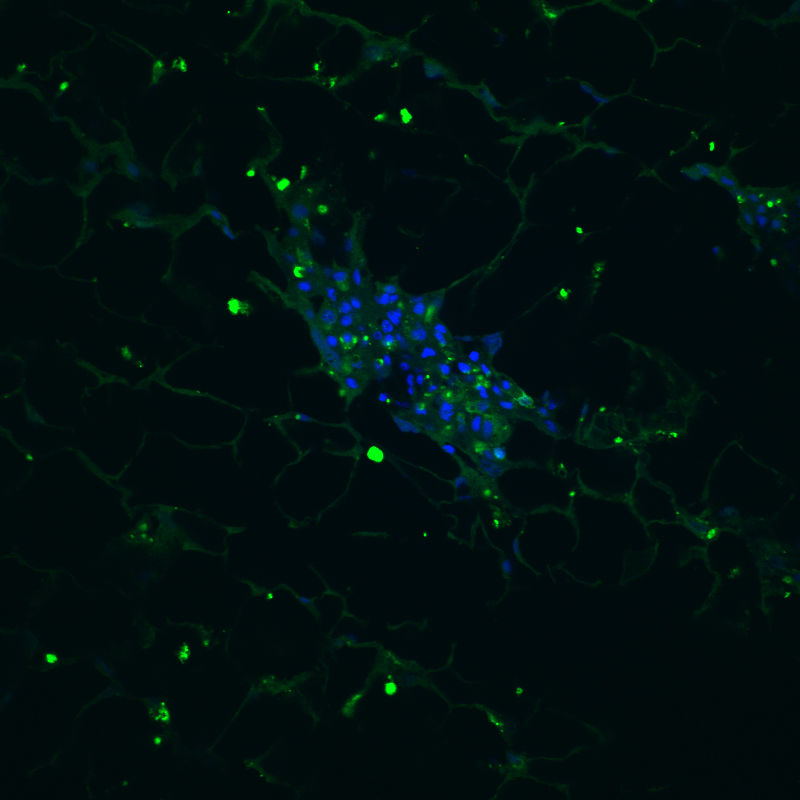

Supplement: Data S1 [file peerj-06-5789-s002.zip › T1/Image0005_1.jpg]

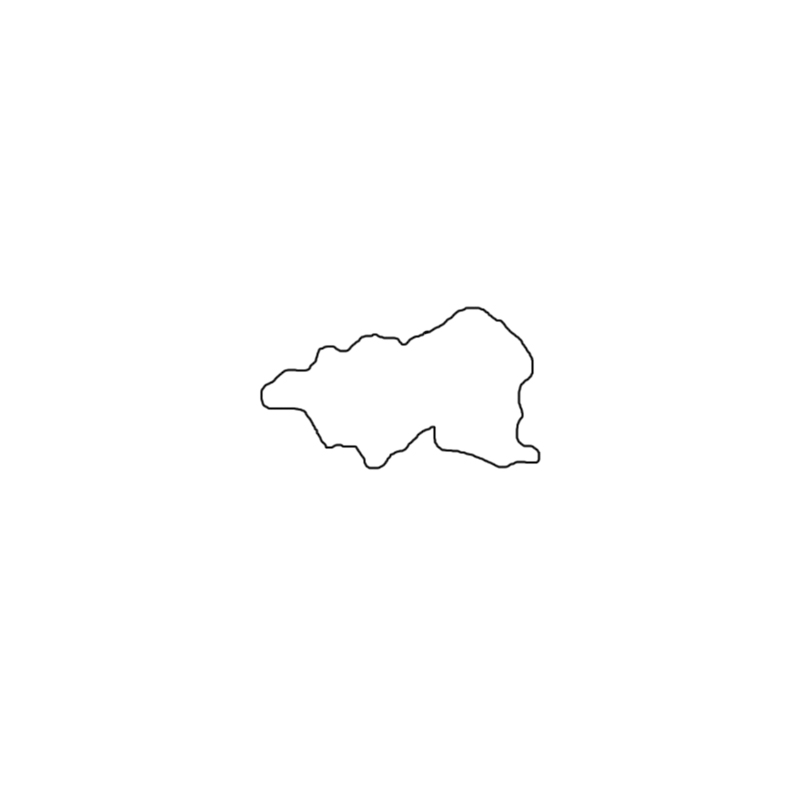

Supplement: Data S1 [file peerj-06-5789-s002.zip › T1/Image0007 GRID.jpg]

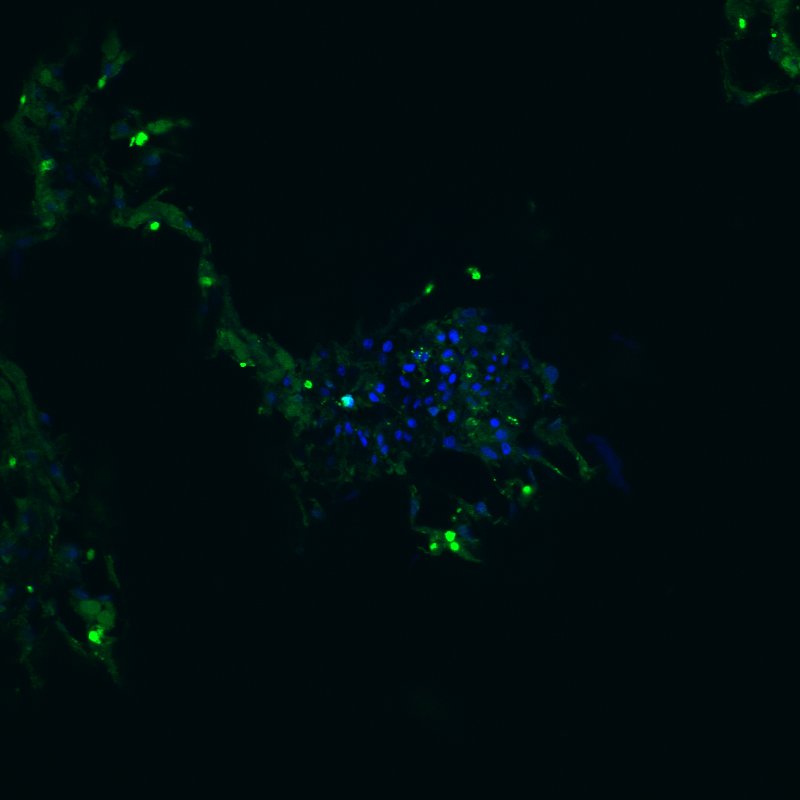

Supplement: Data S1 [file peerj-06-5789-s002.zip › T1/Image0007_1.jpg]

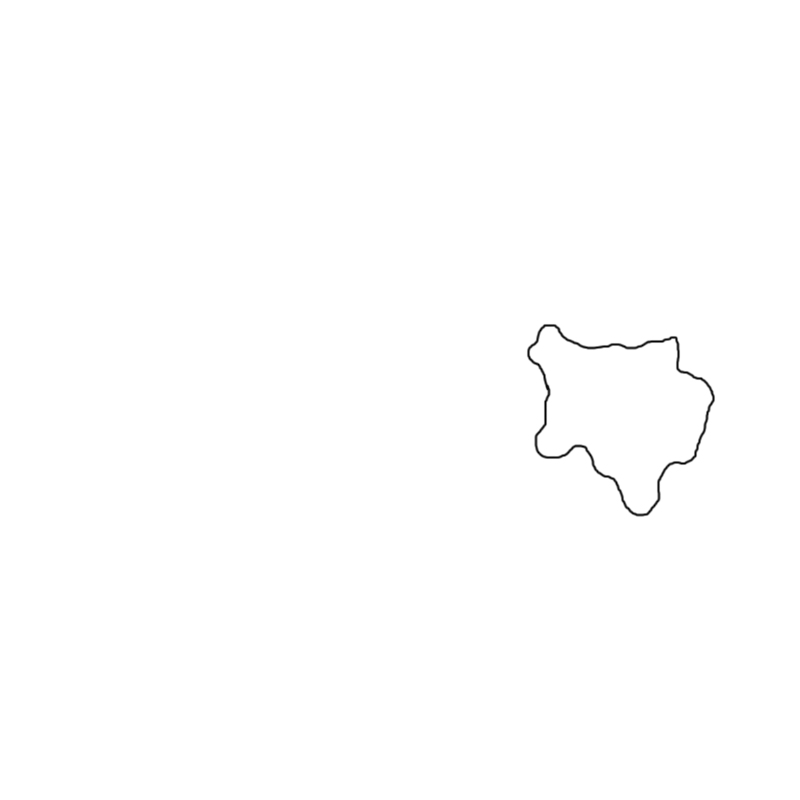

Supplement: Data S1 [file peerj-06-5789-s002.zip › T1/Image0008 GRID.jpg]

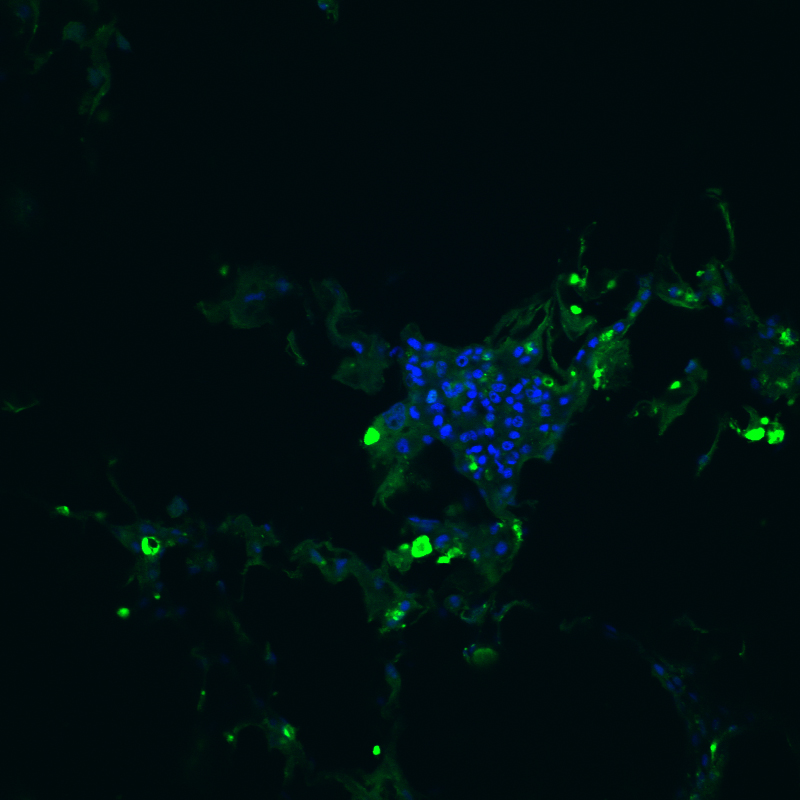

Supplement: Data S1 [file peerj-06-5789-s002.zip › T1/Image0008.jpg]

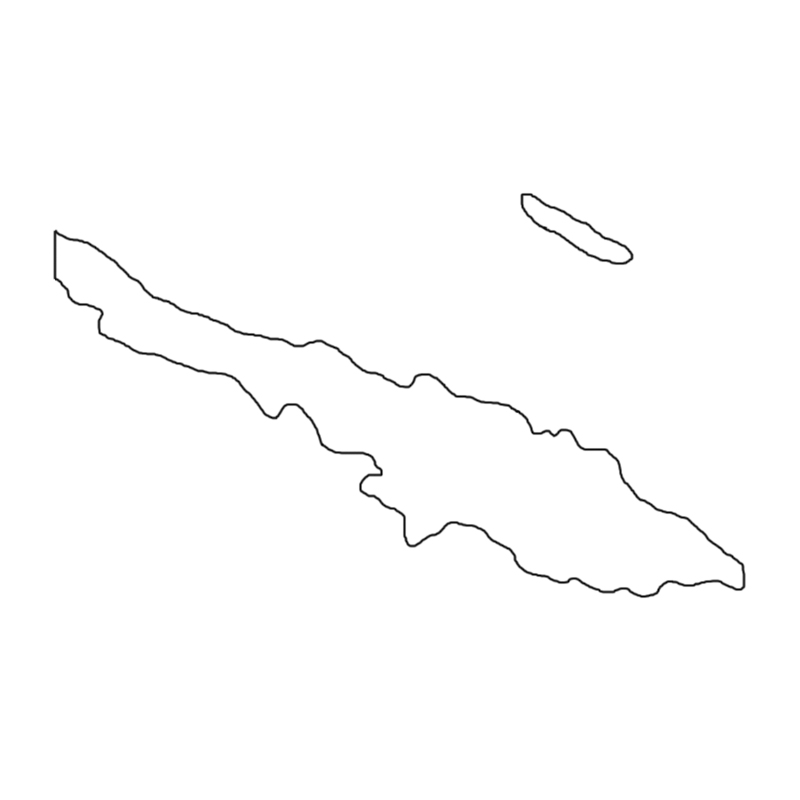

Supplement: Data S1 [file peerj-06-5789-s002.zip › T1/Image0009 GRID.jpg]

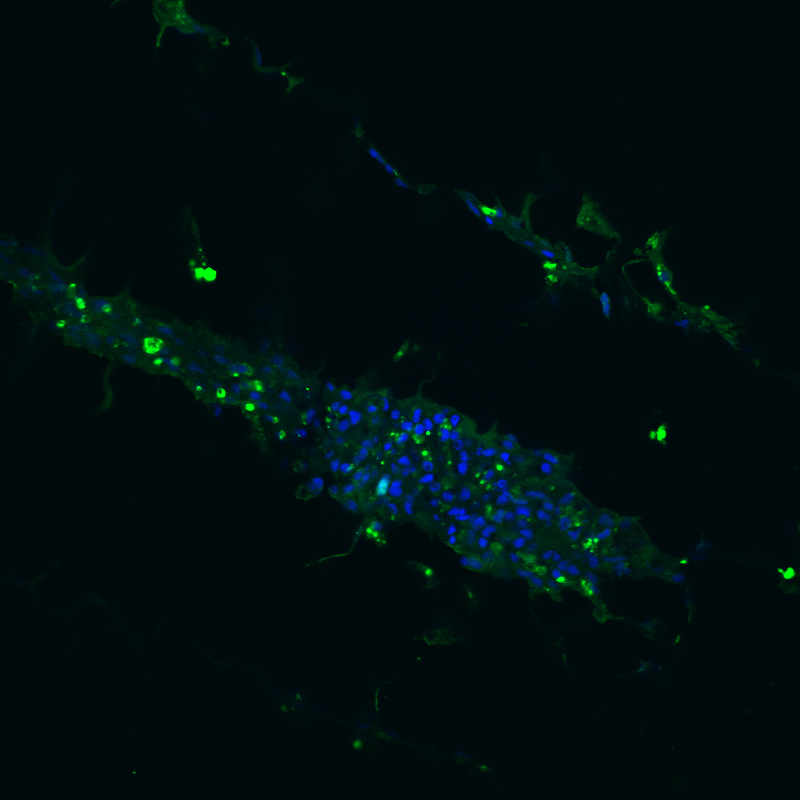

Supplement: Data S1 [file peerj-06-5789-s002.zip › T1/Image0009.jpg]

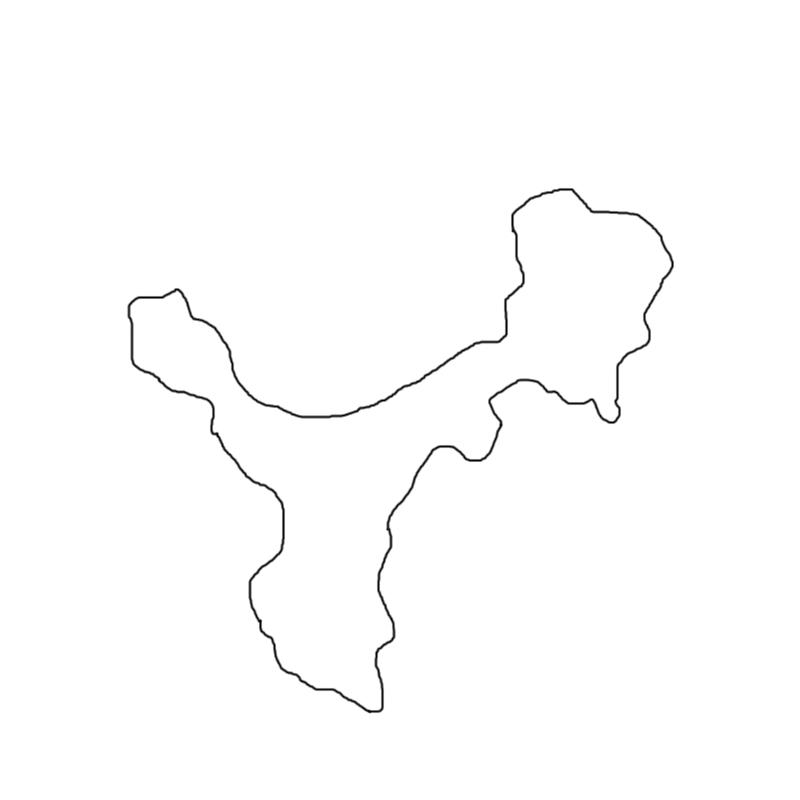

Supplement: Data S1 [file peerj-06-5789-s002.zip › T1/Image0010 GRID.jpg]

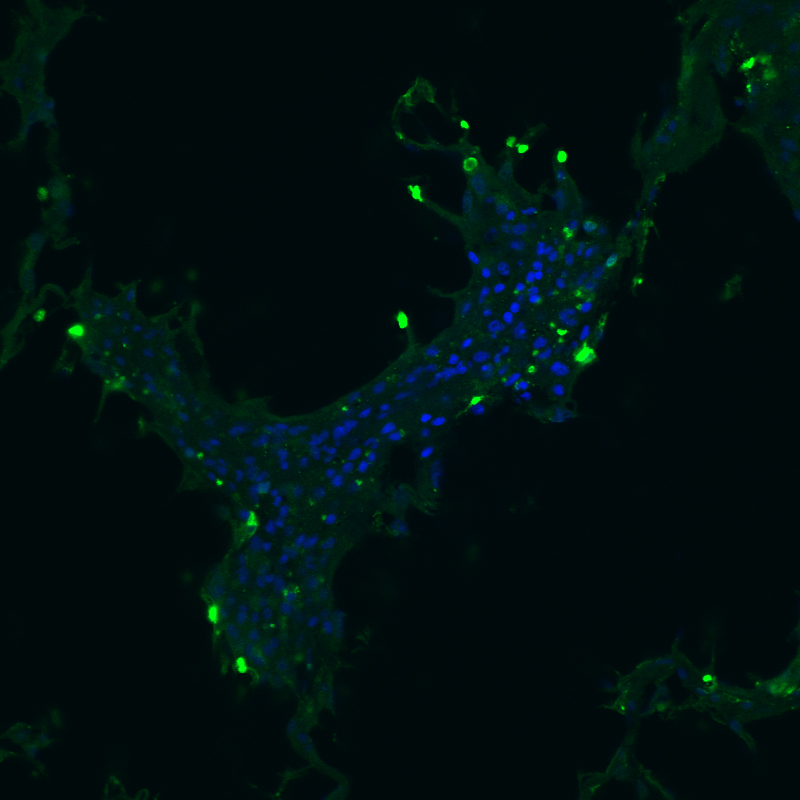

Supplement: Data S1 [file peerj-06-5789-s002.zip › T1/Image0010.jpg]

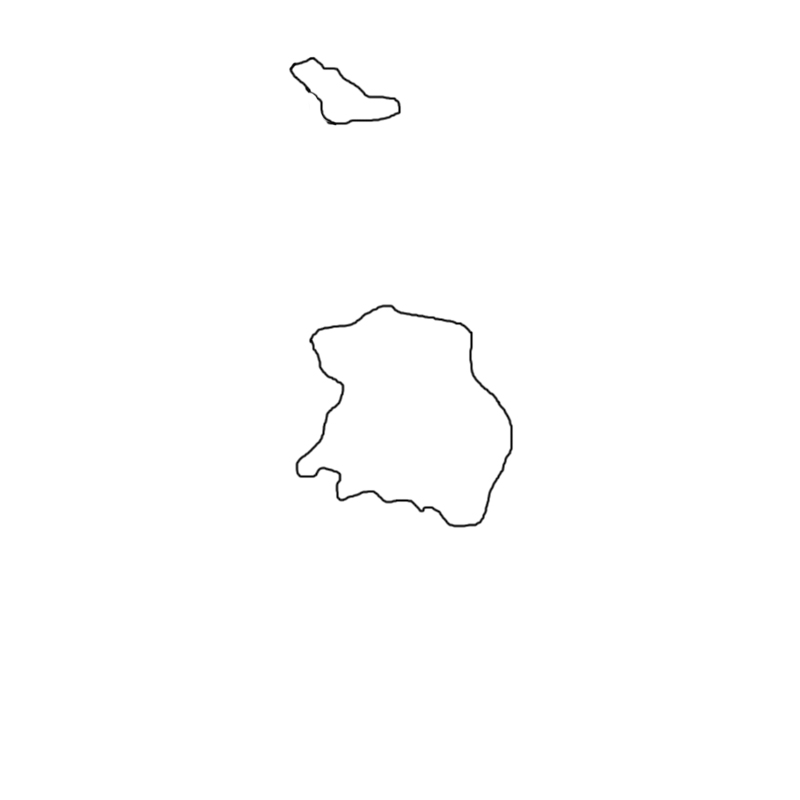

Supplement: Data S1 [file peerj-06-5789-s002.zip › T1/Image0016 GRID.jpg]

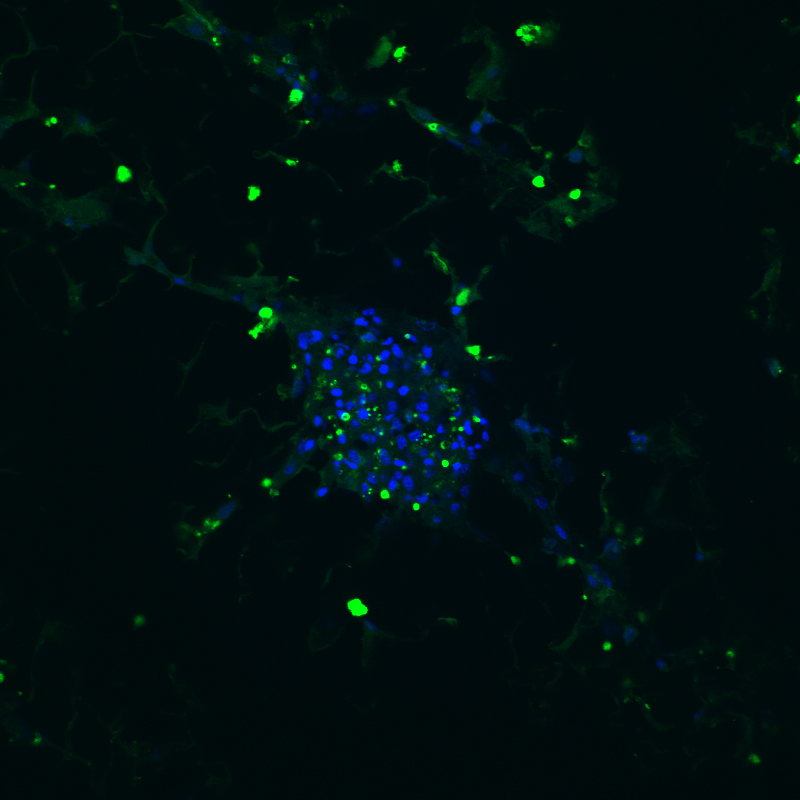

Supplement: Data S1 [file peerj-06-5789-s002.zip › T1/Image0016_1.jpg]

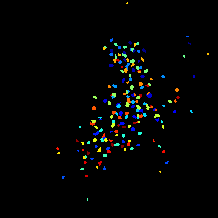

Supplement: Data S1 [file peerj-06-5789-s002.zip › T2/Im11 Nuclei.png]

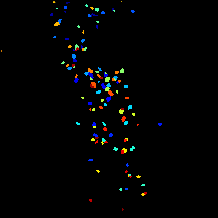

Supplement: Data S1 [file peerj-06-5789-s002.zip › T2/Im12 Nuclei.png]

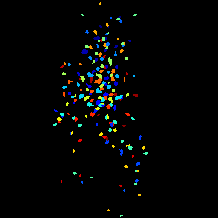

Supplement: Data S1 [file peerj-06-5789-s002.zip › T2/Im13 Nuclei.png]

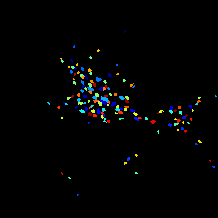

Supplement: Data S1 [file peerj-06-5789-s002.zip › T2/Im14 Nuclei.png]

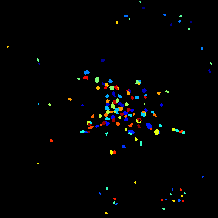

Supplement: Data S1 [file peerj-06-5789-s002.zip › T2/Im16 Nuclei.png]

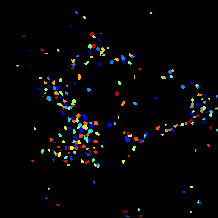

Supplement: Data S1 [file peerj-06-5789-s002.zip › T2/Im17 Nuclei.png]

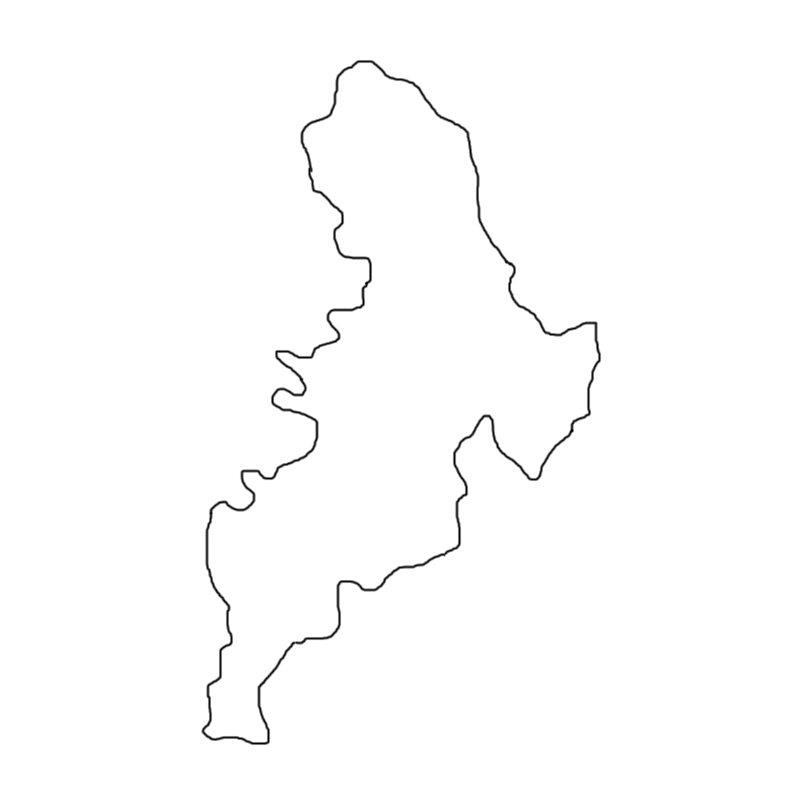

Supplement: Data S1 [file peerj-06-5789-s002.zip › T2/Image0011 GRID.jpg]

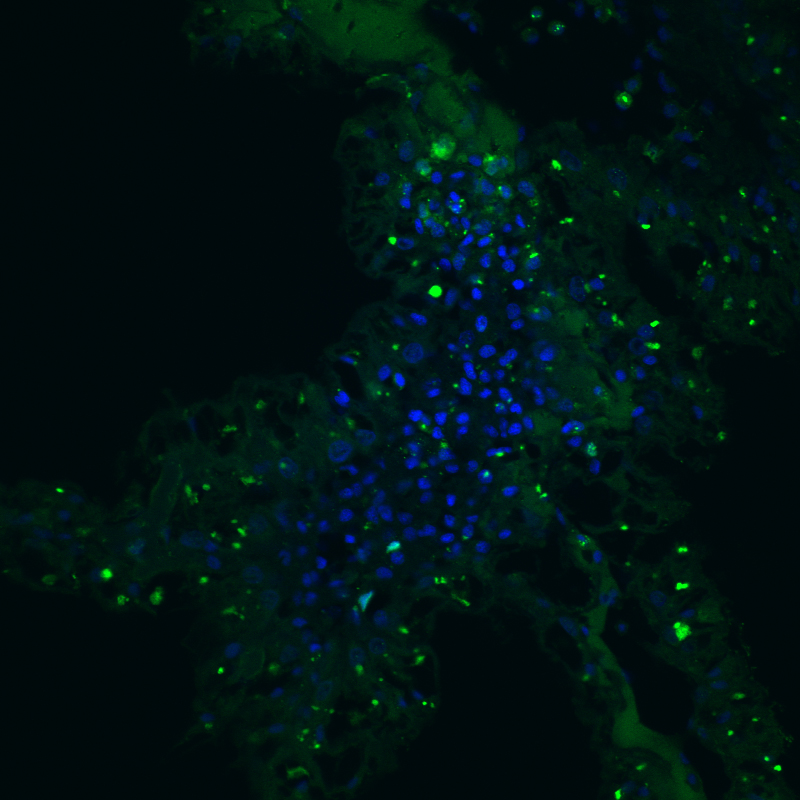

Supplement: Data S1 [file peerj-06-5789-s002.zip › T2/Image0011.jpg]

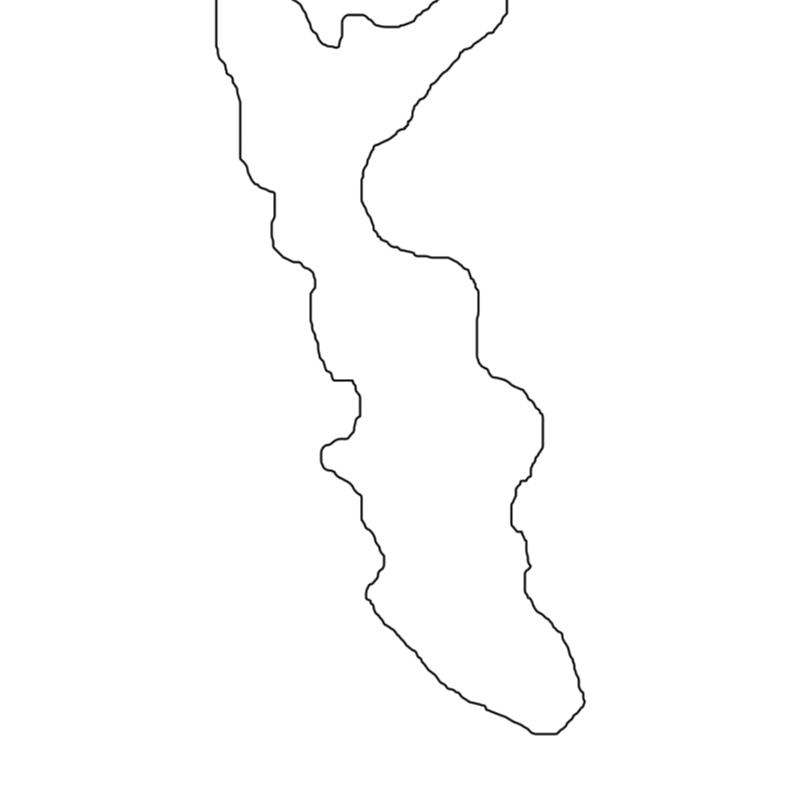

Supplement: Data S1 [file peerj-06-5789-s002.zip › T2/Image0012 GRID.jpg]

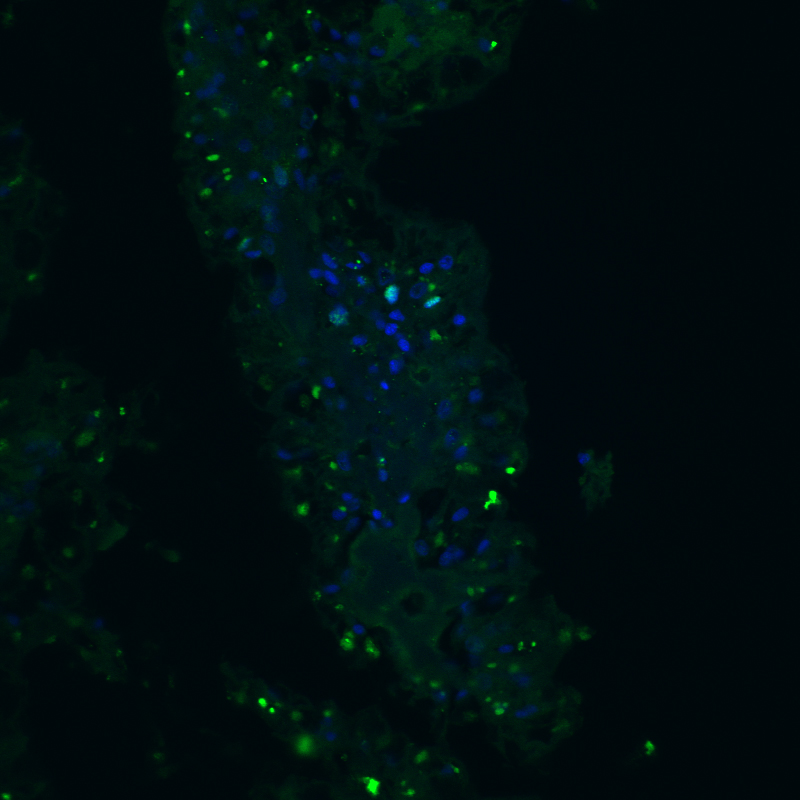

Supplement: Data S1 [file peerj-06-5789-s002.zip › T2/Image0012.jpg]

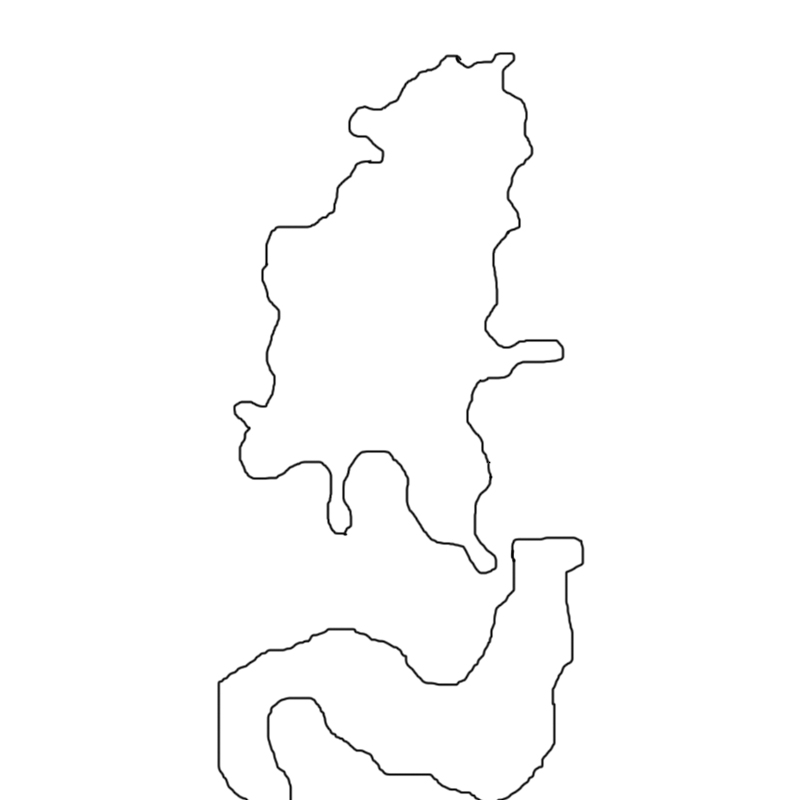

Supplement: Data S1 [file peerj-06-5789-s002.zip › T2/Image0013 GRID.jpg]

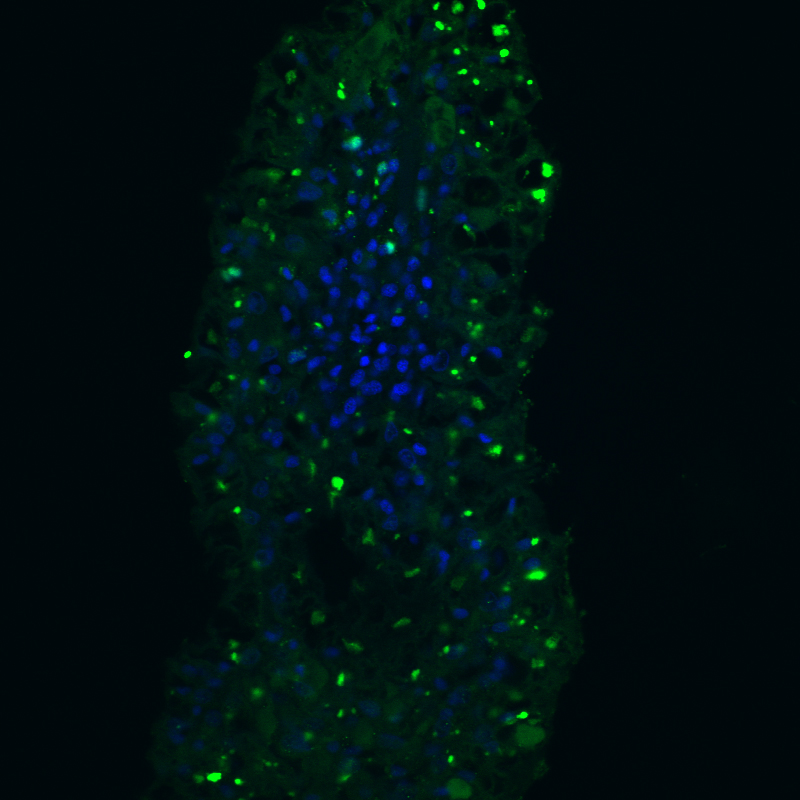

Supplement: Data S1 [file peerj-06-5789-s002.zip › T2/Image0013.jpg]

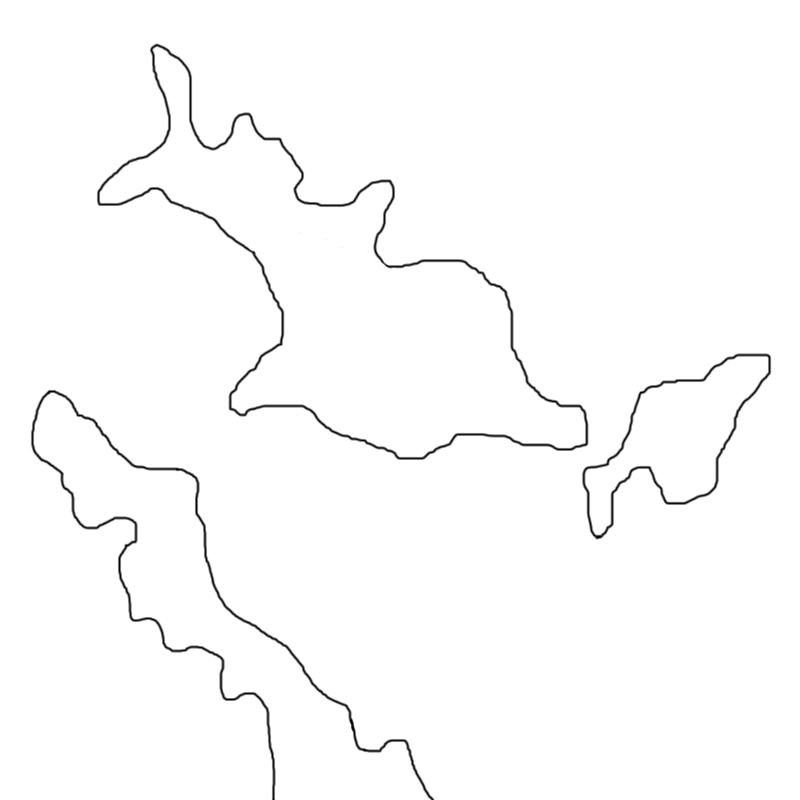

Supplement: Data S1 [file peerj-06-5789-s002.zip › T2/Image0014 GRID.jpg]

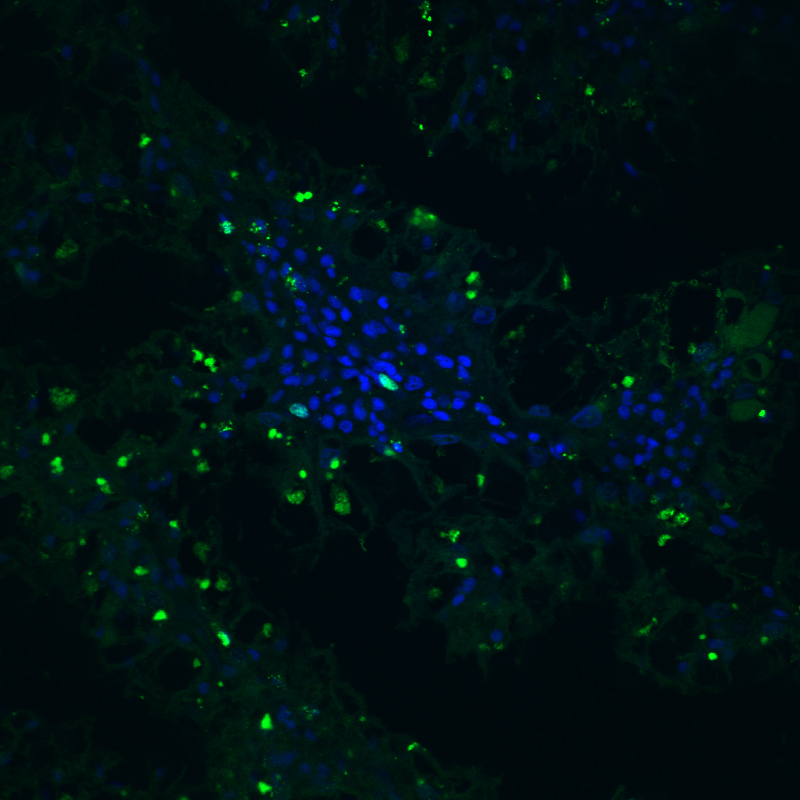

Supplement: Data S1 [file peerj-06-5789-s002.zip › T2/Image0014.jpg]

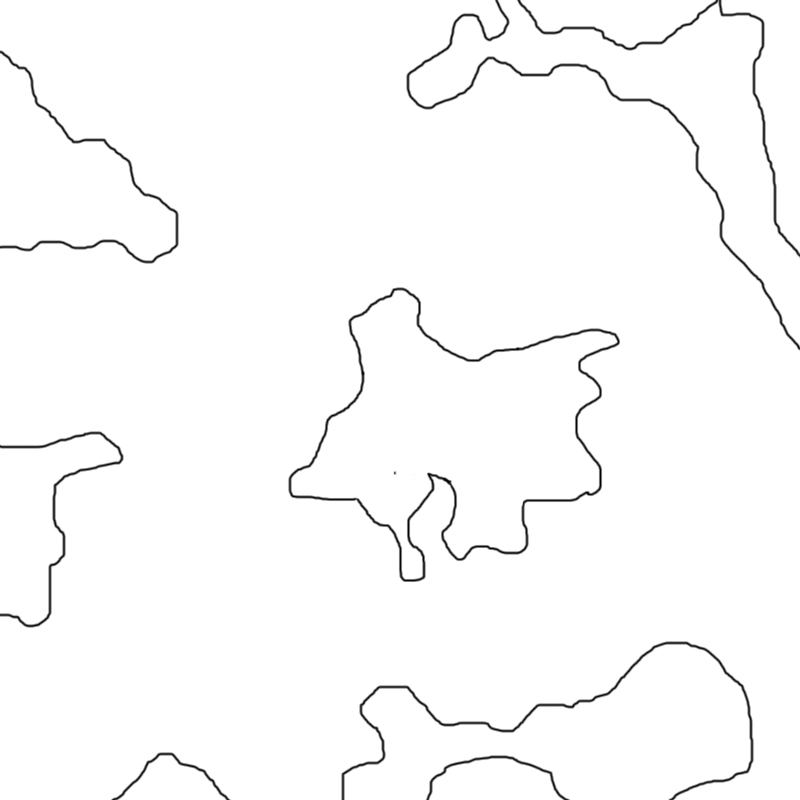

Supplement: Data S1 [file peerj-06-5789-s002.zip › T2/Image0016 GRID.jpg]

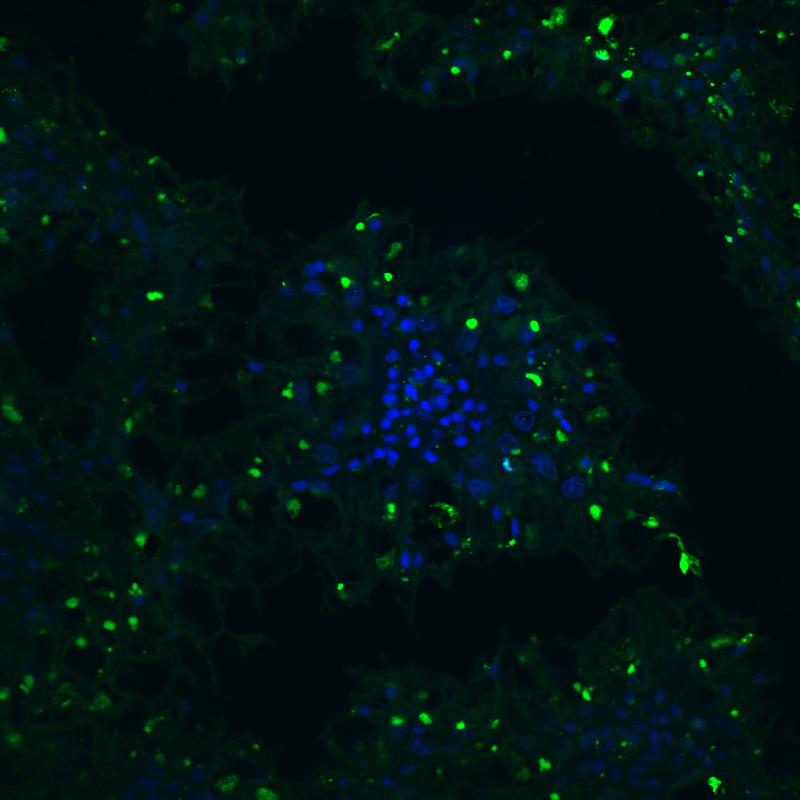

Supplement: Data S1 [file peerj-06-5789-s002.zip › T2/Image0016.jpg]

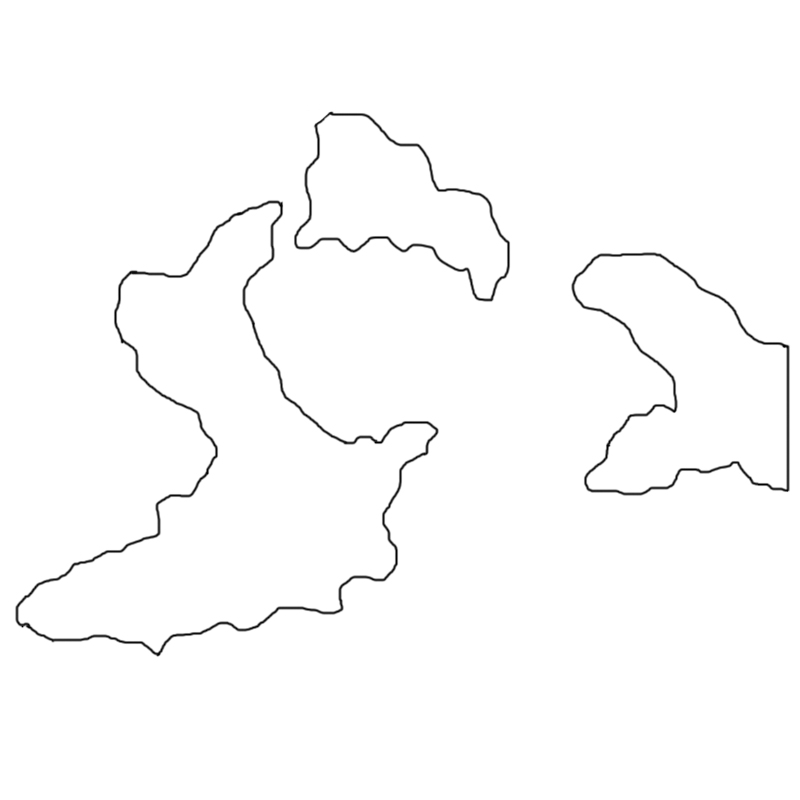

Supplement: Data S1 [file peerj-06-5789-s002.zip › T2/Image0017 GRID.jpg]

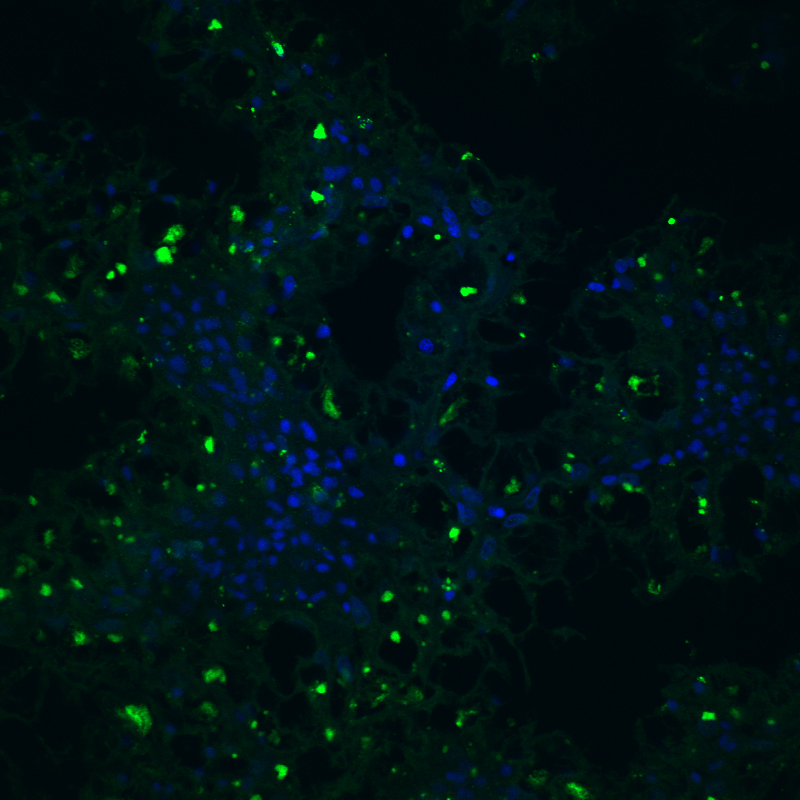

Supplement: Data S1 [file peerj-06-5789-s002.zip › T2/Image0017.jpg]

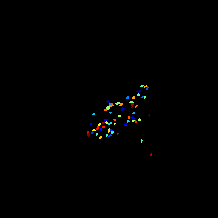

Supplement: Data S1 [file peerj-06-5789-s002.zip › T3/Im01 Nuclei.png]

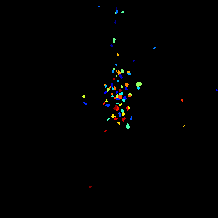

Supplement: Data S1 [file peerj-06-5789-s002.zip › T3/Im03 Nuclei.png]

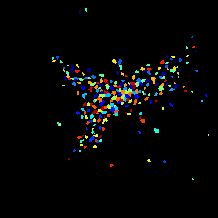

Supplement: Data S1 [file peerj-06-5789-s002.zip › T3/Im04 Nuclei.png]

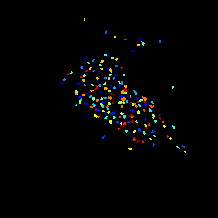

Supplement: Data S1 [file peerj-06-5789-s002.zip › T3/Im05 Nuclei.png]
