# Supplementary figures and images for: Assessment of the kidney and lung as immune barriers and hematopoietic sites in the invasive apple snail Pomacea canaliculata (part 1 of 2)
Source: PeerJ. 2018 Oct 12;6:e5789. doi: 10.7717/peerj.5789 (PMC6187997; doi:10.7717/peerj.5789)

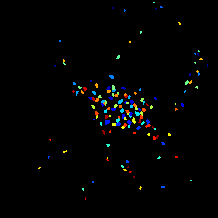

Supplement: Data S1 [file peerj-06-5789-s002.zip › C1/Im02 Nuclei.png]

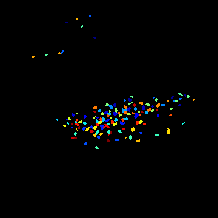

Supplement: Data S1 [file peerj-06-5789-s002.zip › C1/Im03 Nuclei.png]

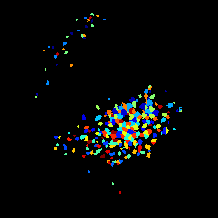

Supplement: Data S1 [file peerj-06-5789-s002.zip › C1/Im04 Nuclei.png]

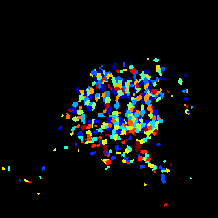

Supplement: Data S1 [file peerj-06-5789-s002.zip › C1/Im06 Nuclei.png]

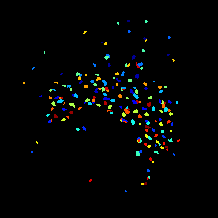

Supplement: Data S1 [file peerj-06-5789-s002.zip › C1/Im07 Nuclei.png]

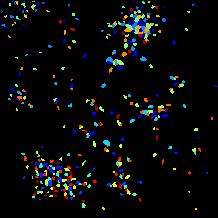

Supplement: Data S1 [file peerj-06-5789-s002.zip › C1/Im08 Nuclei.png]

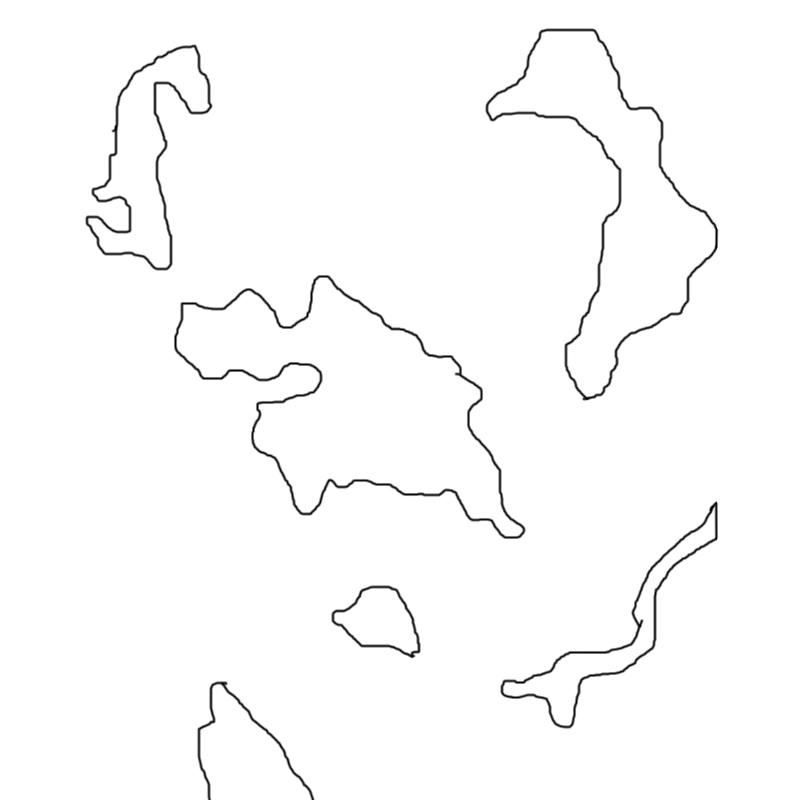

Supplement: Data S1 [file peerj-06-5789-s002.zip › C1/Image0002 GRID.jpg]

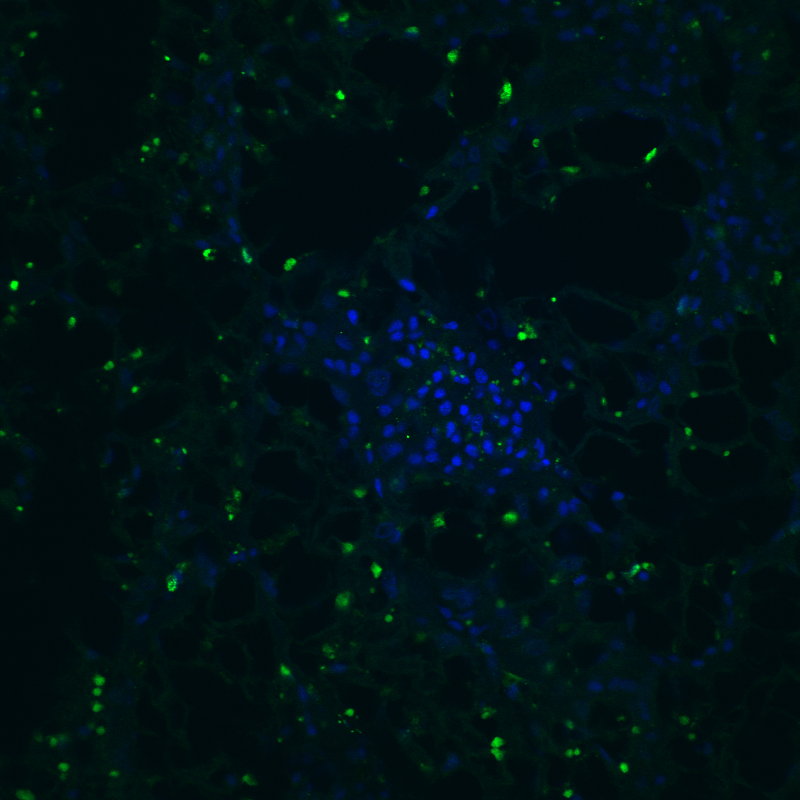

Supplement: Data S1 [file peerj-06-5789-s002.zip › C1/Image0002.jpg]

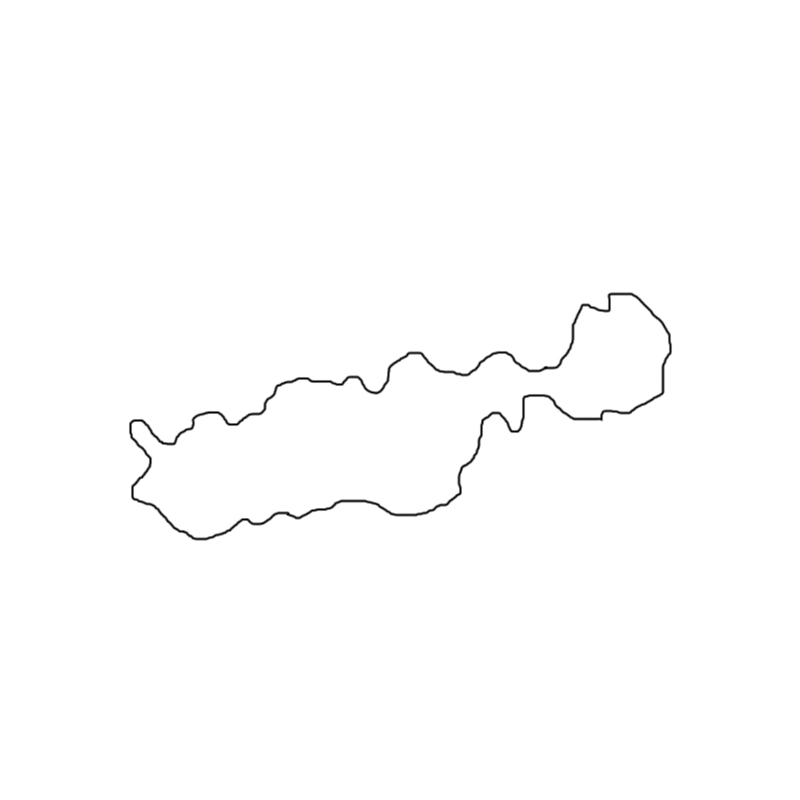

Supplement: Data S1 [file peerj-06-5789-s002.zip › C1/Image0003 GRID.jpg]

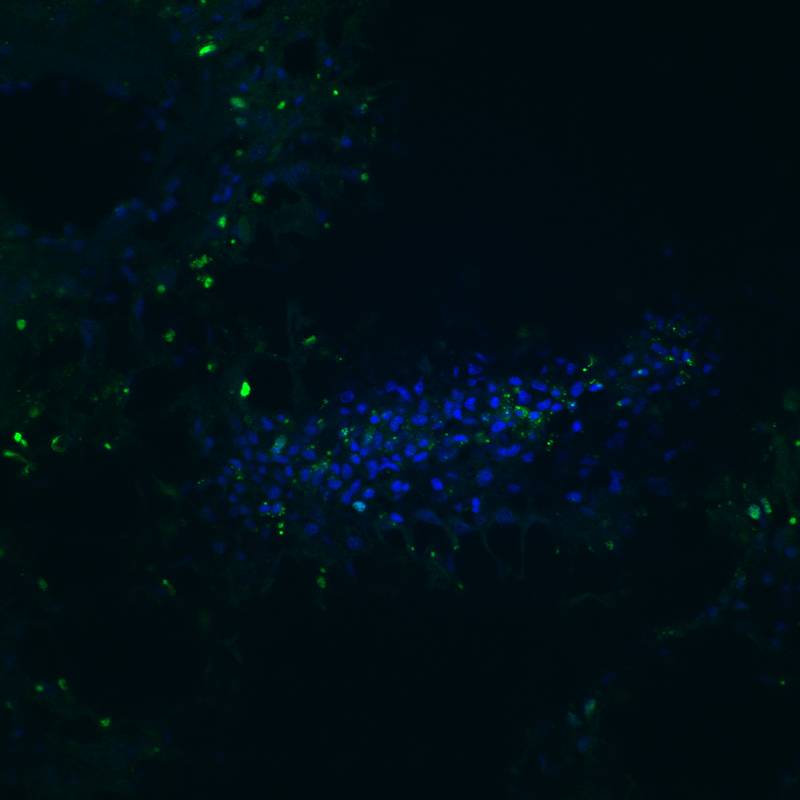

Supplement: Data S1 [file peerj-06-5789-s002.zip › C1/Image0003.jpg]

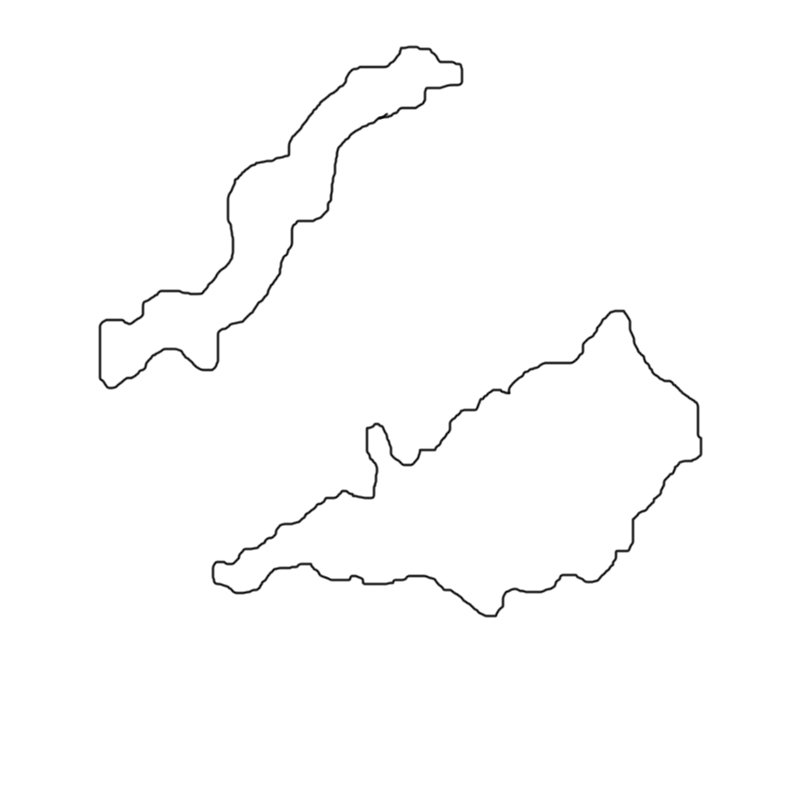

Supplement: Data S1 [file peerj-06-5789-s002.zip › C1/Image0004 GRID.jpg]

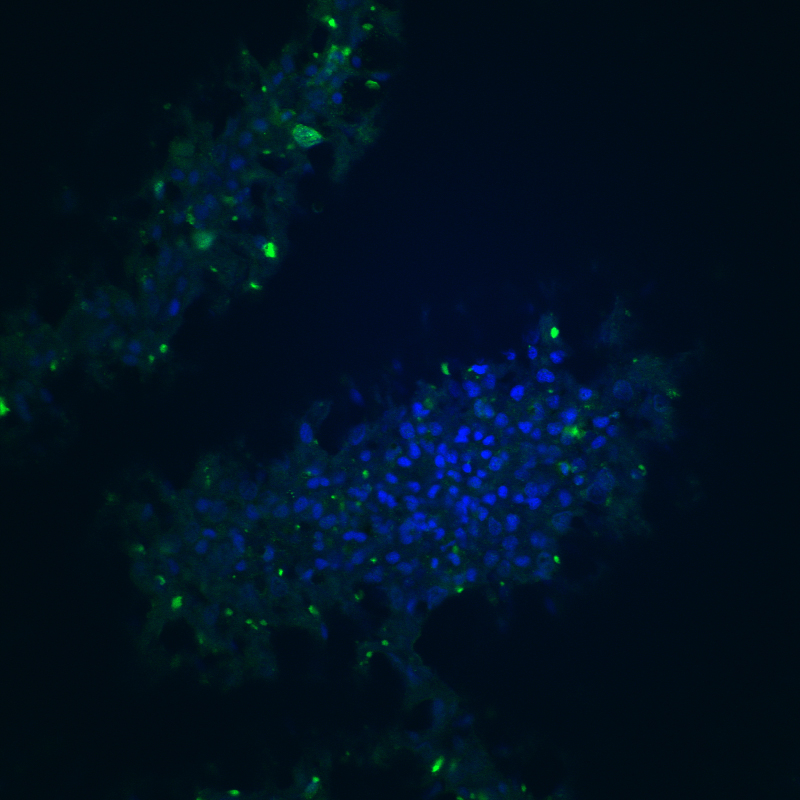

Supplement: Data S1 [file peerj-06-5789-s002.zip › C1/Image0004.jpg]

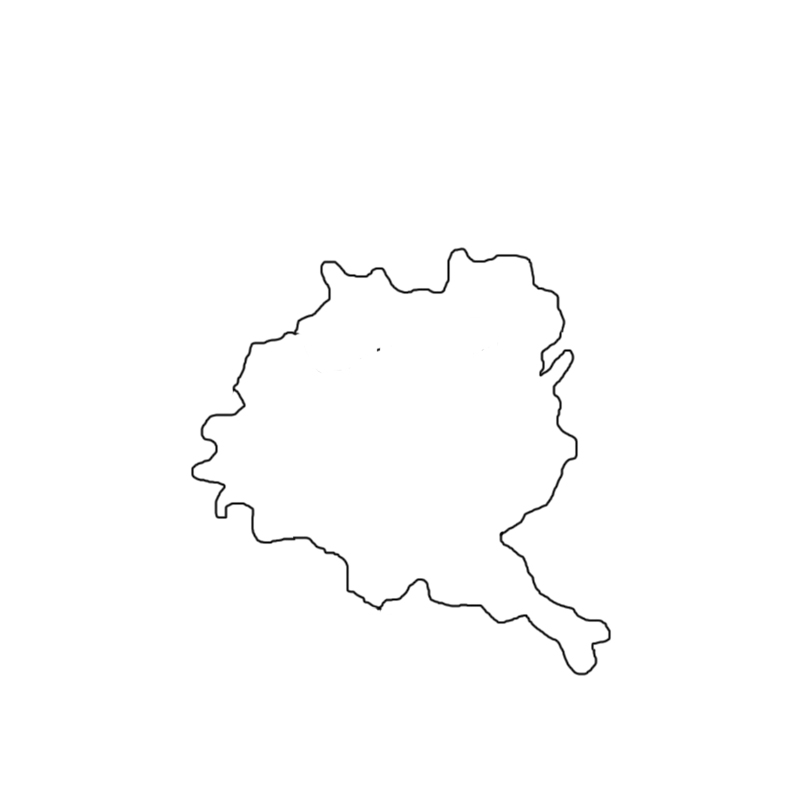

Supplement: Data S1 [file peerj-06-5789-s002.zip › C1/Image0006 GRID.jpg]

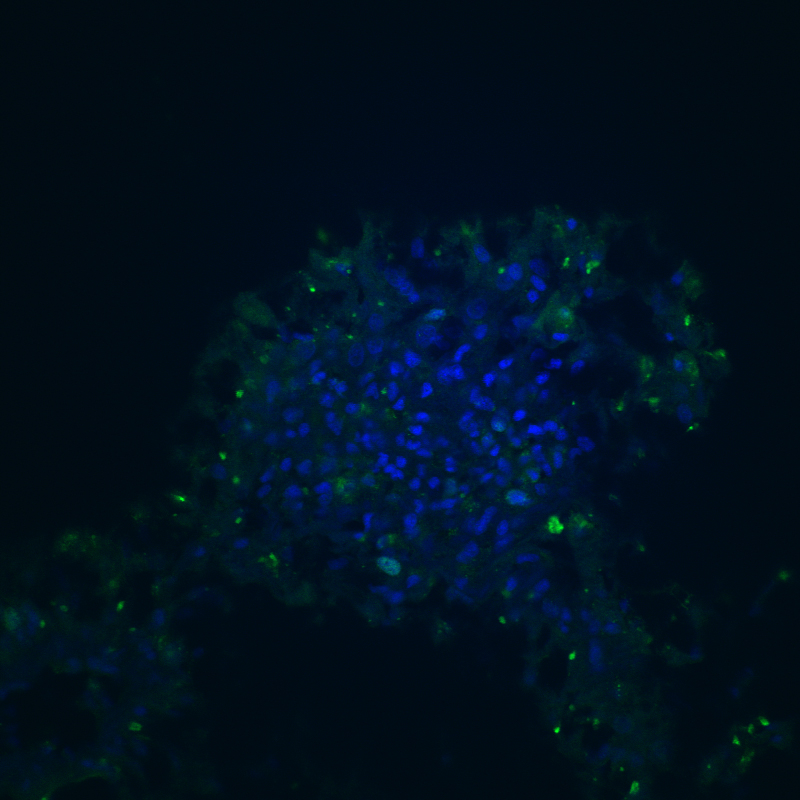

Supplement: Data S1 [file peerj-06-5789-s002.zip › C1/Image0006.jpg]

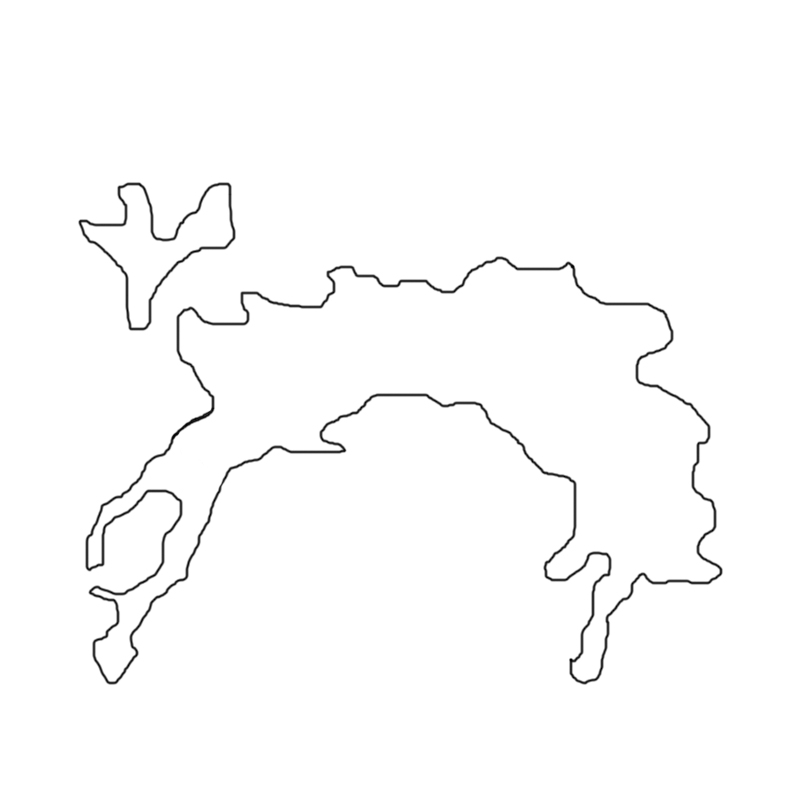

Supplement: Data S1 [file peerj-06-5789-s002.zip › C1/Image0007 GRID.jpg]

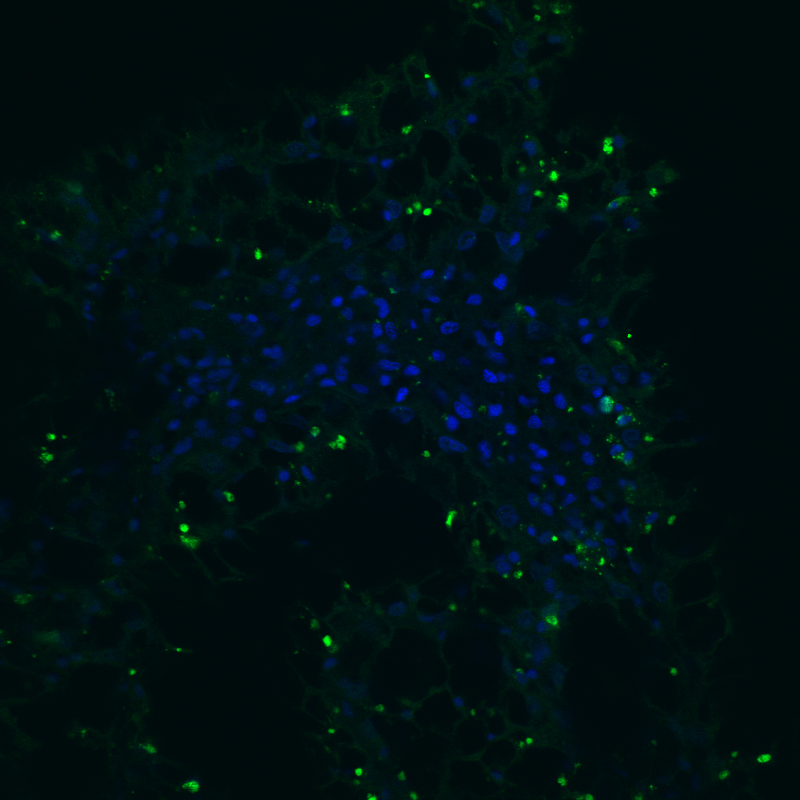

Supplement: Data S1 [file peerj-06-5789-s002.zip › C1/Image0007.jpg]

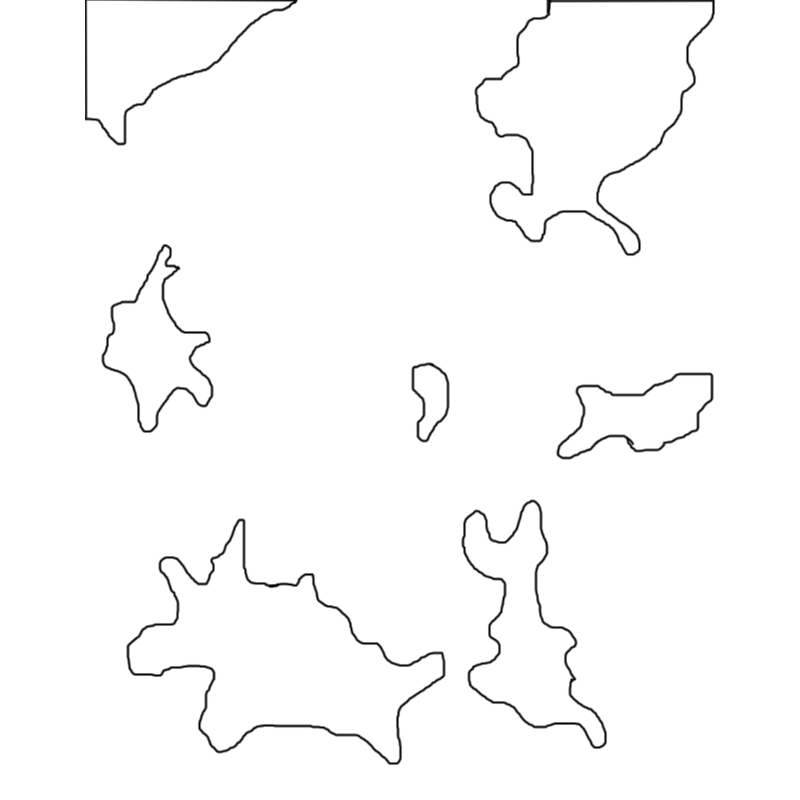

Supplement: Data S1 [file peerj-06-5789-s002.zip › C1/Image0008 GRID.jpg]

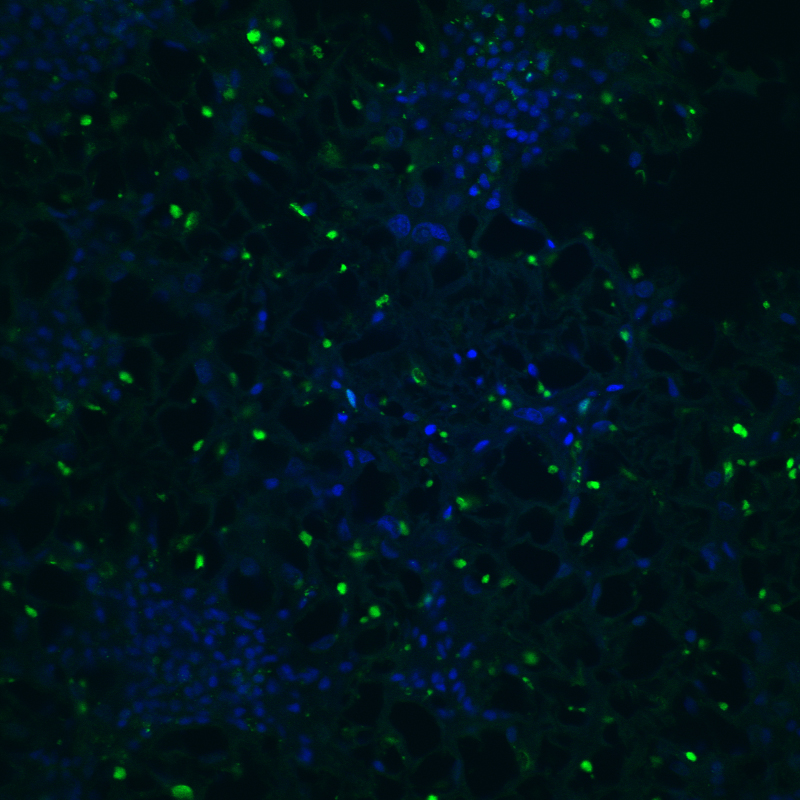

Supplement: Data S1 [file peerj-06-5789-s002.zip › C1/Image0008.jpg]

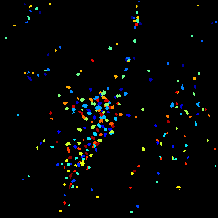

Supplement: Data S1 [file peerj-06-5789-s002.zip › C2/Im08 Nuclei.png]

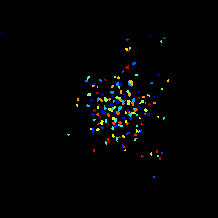

Supplement: Data S1 [file peerj-06-5789-s002.zip › C2/Im09 Nuclei.png]

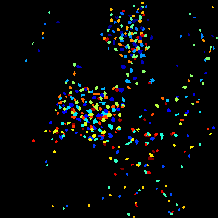

Supplement: Data S1 [file peerj-06-5789-s002.zip › C2/Im10 Nuclei.png]

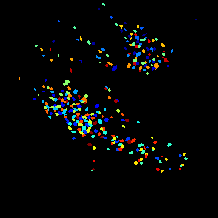

Supplement: Data S1 [file peerj-06-5789-s002.zip › C2/Im11 Nuclei.png]

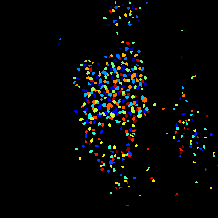

Supplement: Data S1 [file peerj-06-5789-s002.zip › C2/Im12 Nuclei.png]

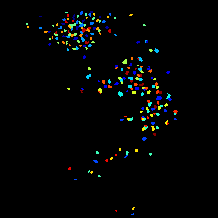

Supplement: Data S1 [file peerj-06-5789-s002.zip › C2/Im13 Nuclei.png]

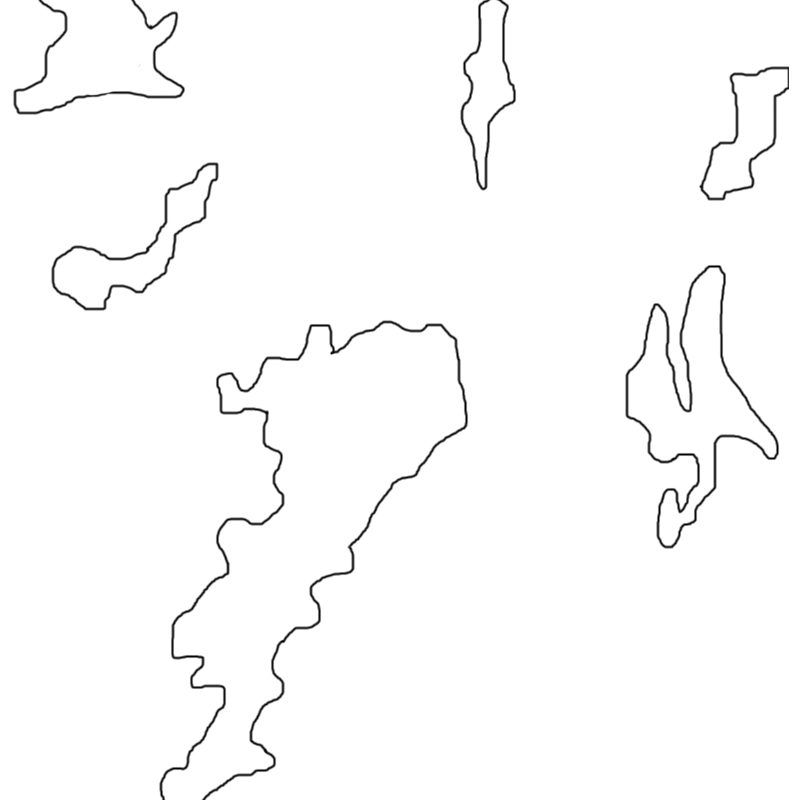

Supplement: Data S1 [file peerj-06-5789-s002.zip › C2/Image0008 GRID.jpg]

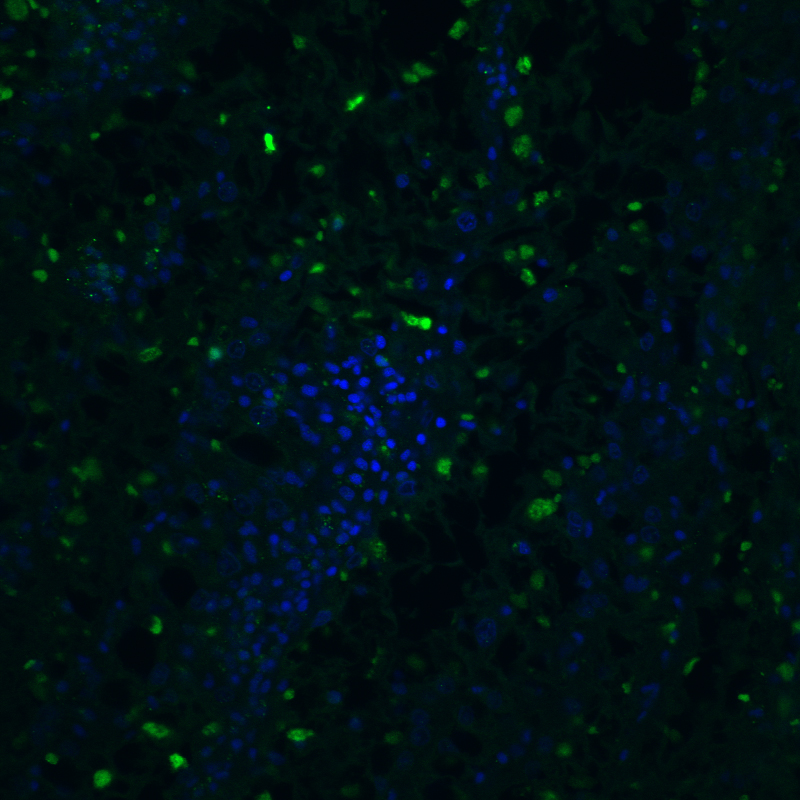

Supplement: Data S1 [file peerj-06-5789-s002.zip › C2/Image0008.jpg]

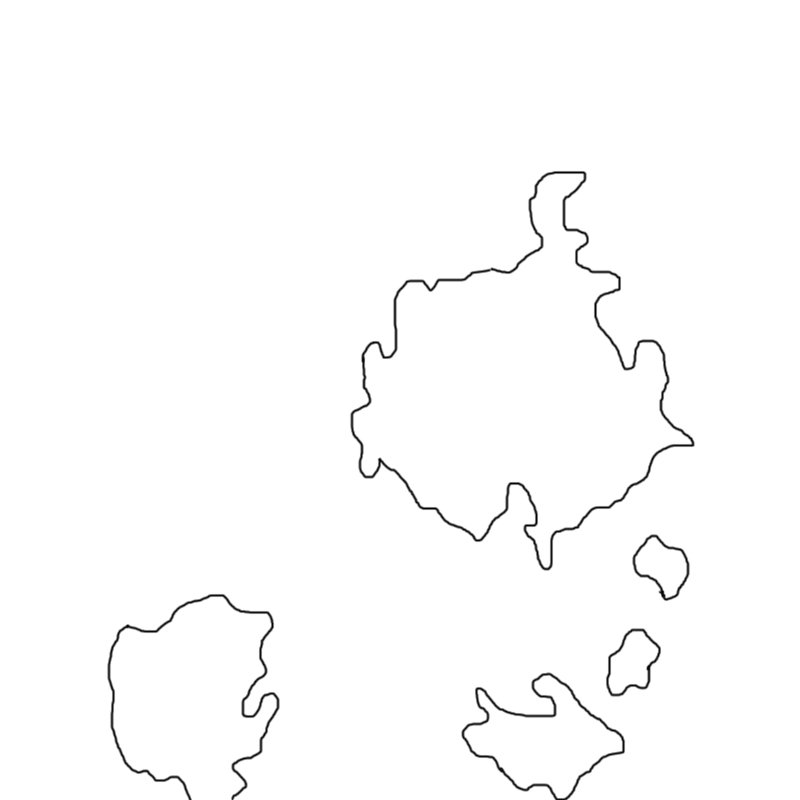

Supplement: Data S1 [file peerj-06-5789-s002.zip › C2/Image0009 GRID.jpg]

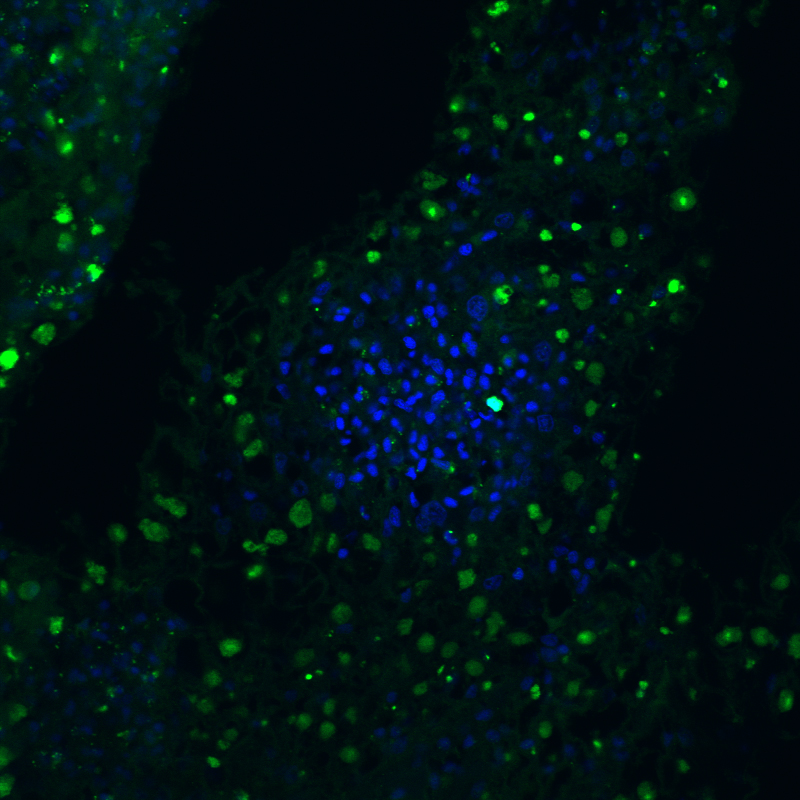

Supplement: Data S1 [file peerj-06-5789-s002.zip › C2/Image0009.jpg]

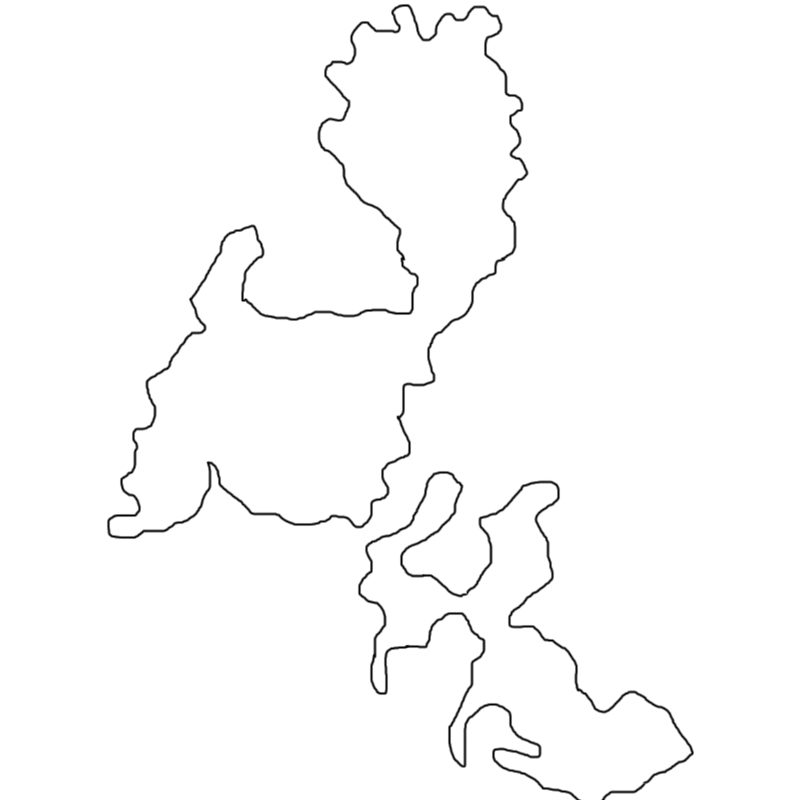

Supplement: Data S1 [file peerj-06-5789-s002.zip › C2/Image0010 GRID.jpg]

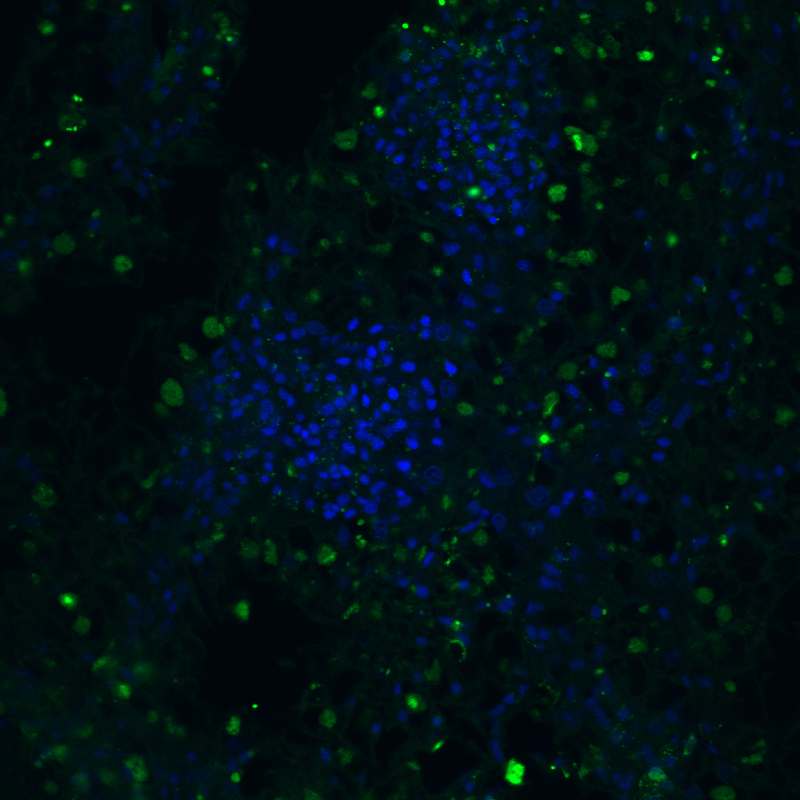

Supplement: Data S1 [file peerj-06-5789-s002.zip › C2/Image0010.jpg]

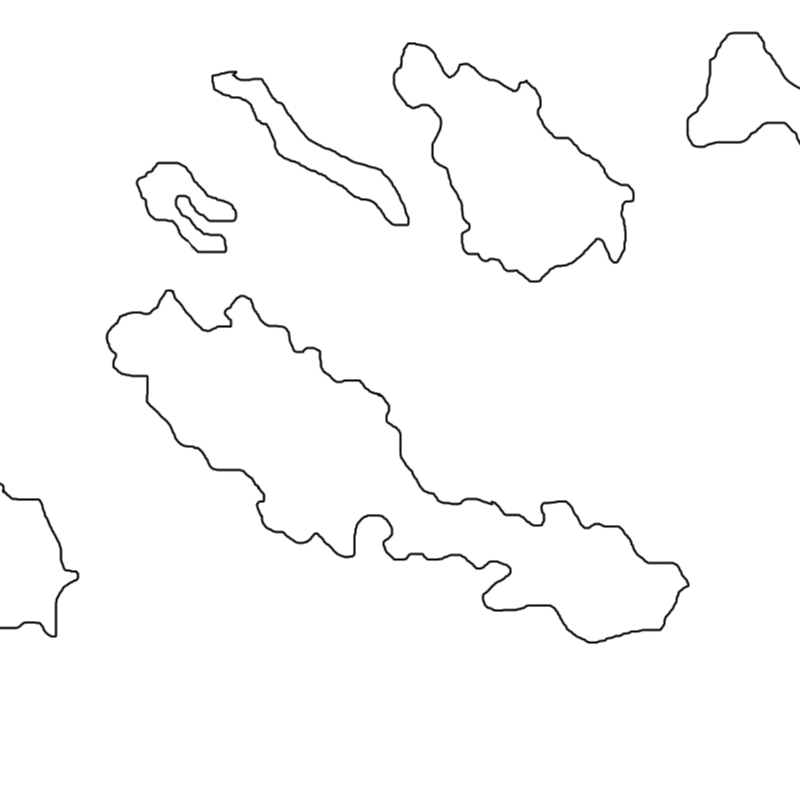

Supplement: Data S1 [file peerj-06-5789-s002.zip › C2/Image0011 GRID.jpg]

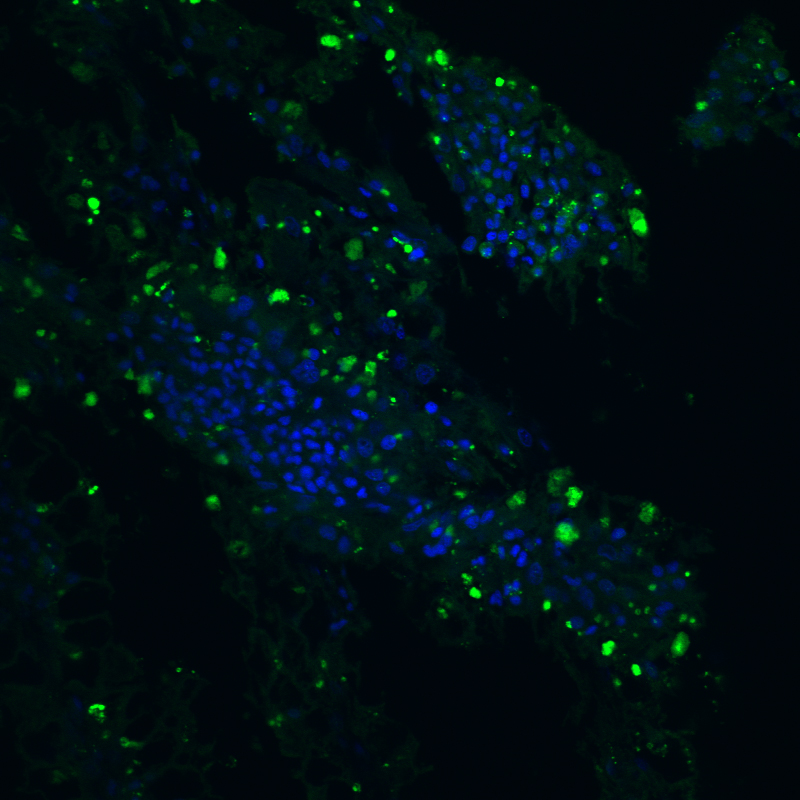

Supplement: Data S1 [file peerj-06-5789-s002.zip › C2/Image0011.jpg]

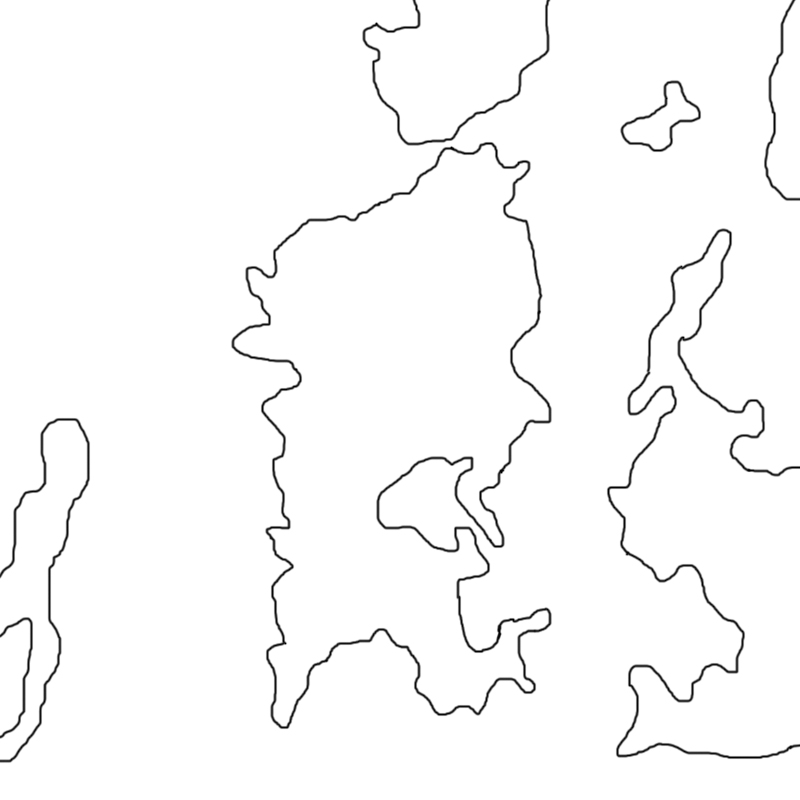

Supplement: Data S1 [file peerj-06-5789-s002.zip › C2/Image0012 GRID.jpg]

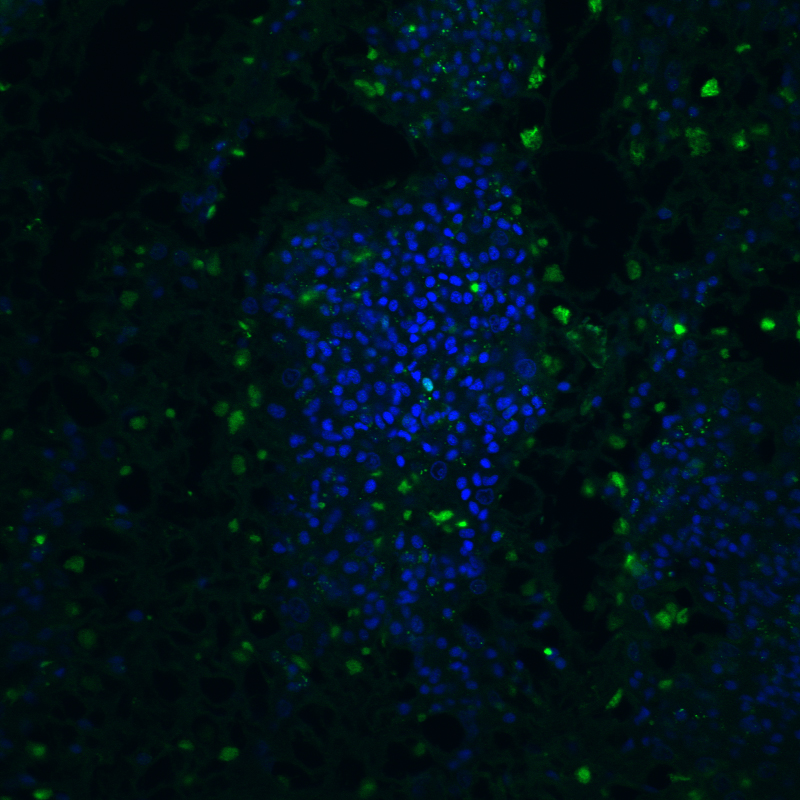

Supplement: Data S1 [file peerj-06-5789-s002.zip › C2/Image0012.jpg]

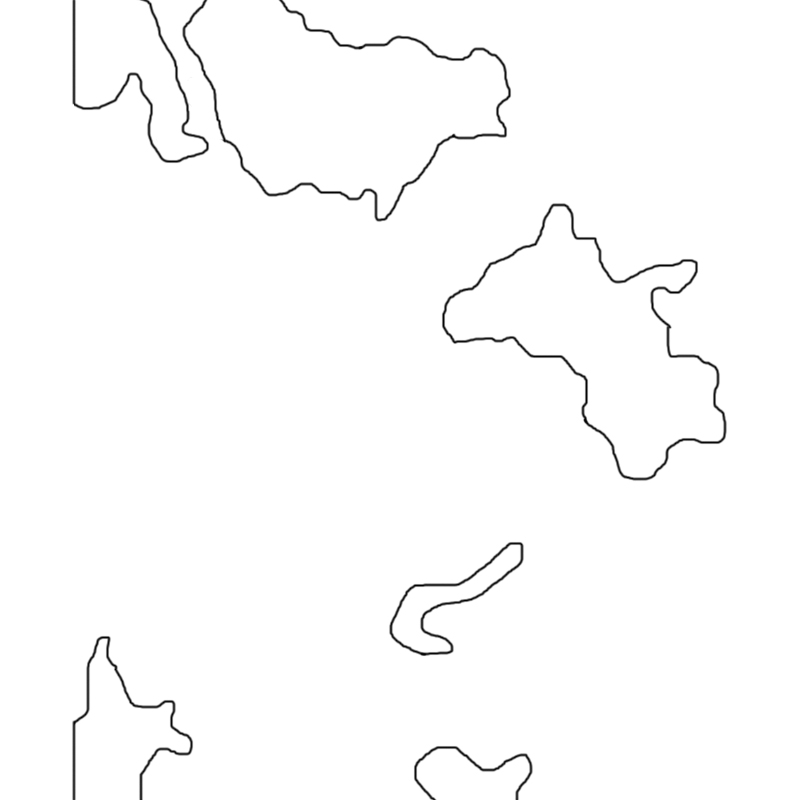

Supplement: Data S1 [file peerj-06-5789-s002.zip › C2/Image0013 GRID.jpg]

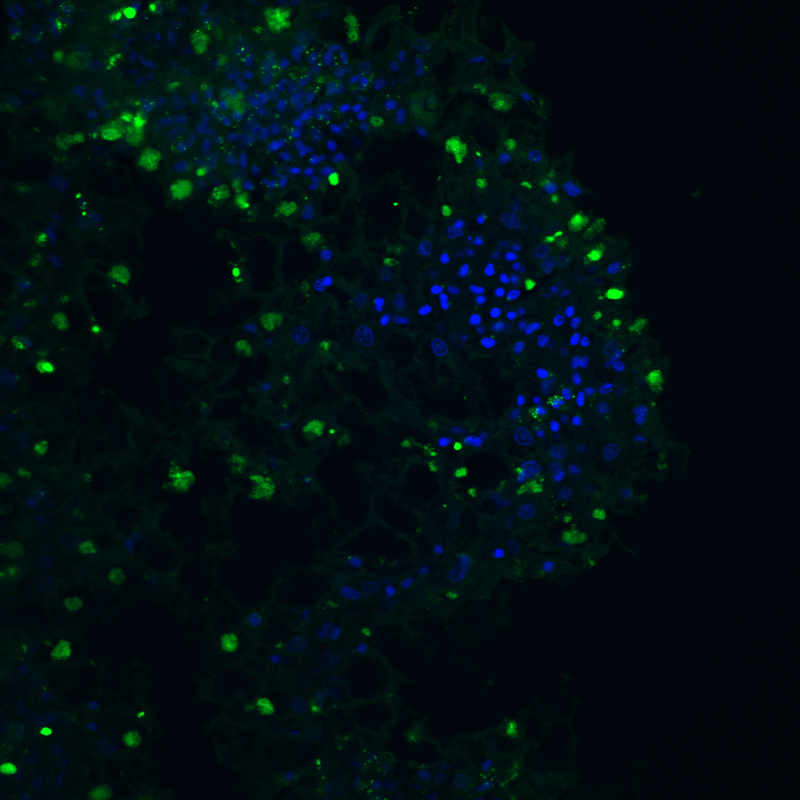

Supplement: Data S1 [file peerj-06-5789-s002.zip › C2/Image0013.jpg]

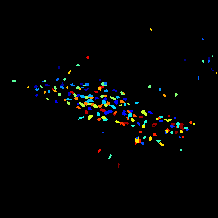

Supplement: Data S1 [file peerj-06-5789-s002.zip › C3/Im14 Nuclei.png]

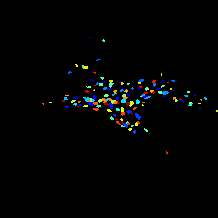

Supplement: Data S1 [file peerj-06-5789-s002.zip › C3/Im15 Nuclei.png]

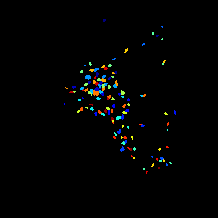

Supplement: Data S1 [file peerj-06-5789-s002.zip › C3/Im17 Nuclei.png]

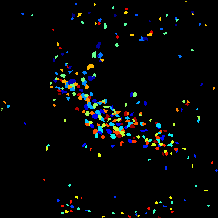

Supplement: Data S1 [file peerj-06-5789-s002.zip › C3/Im18 Nuclei.png]

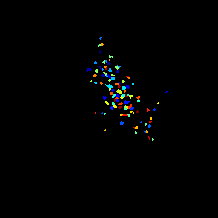

Supplement: Data S1 [file peerj-06-5789-s002.zip › C3/Im19 Nuclei.png]

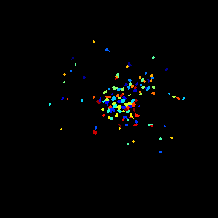

Supplement: Data S1 [file peerj-06-5789-s002.zip › C3/Im20 Nuclei.png]

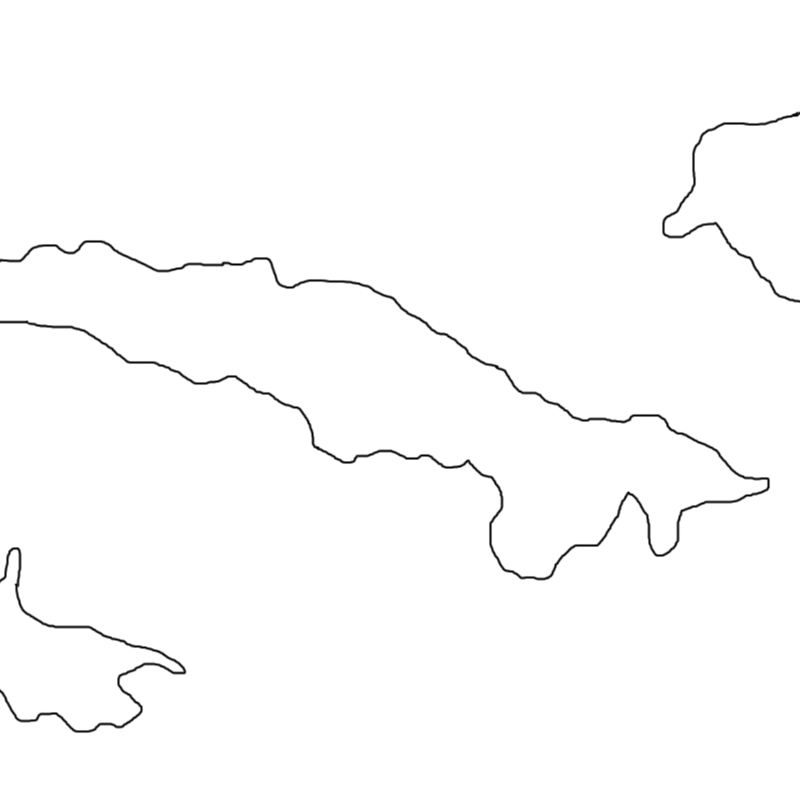

Supplement: Data S1 [file peerj-06-5789-s002.zip › C3/Image0014 GRID.jpg]

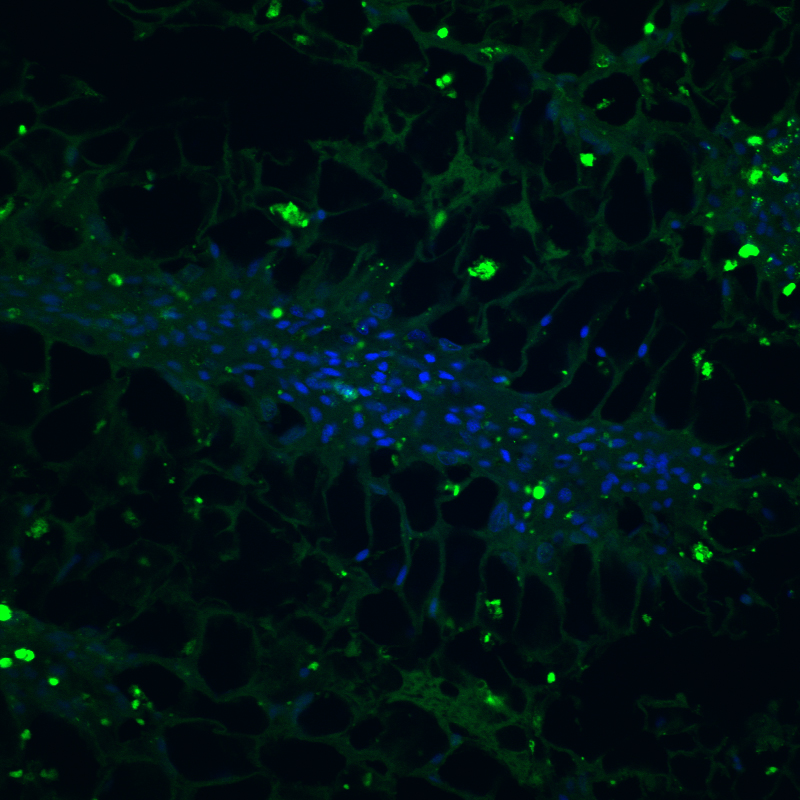

Supplement: Data S1 [file peerj-06-5789-s002.zip › C3/Image0014.jpg]

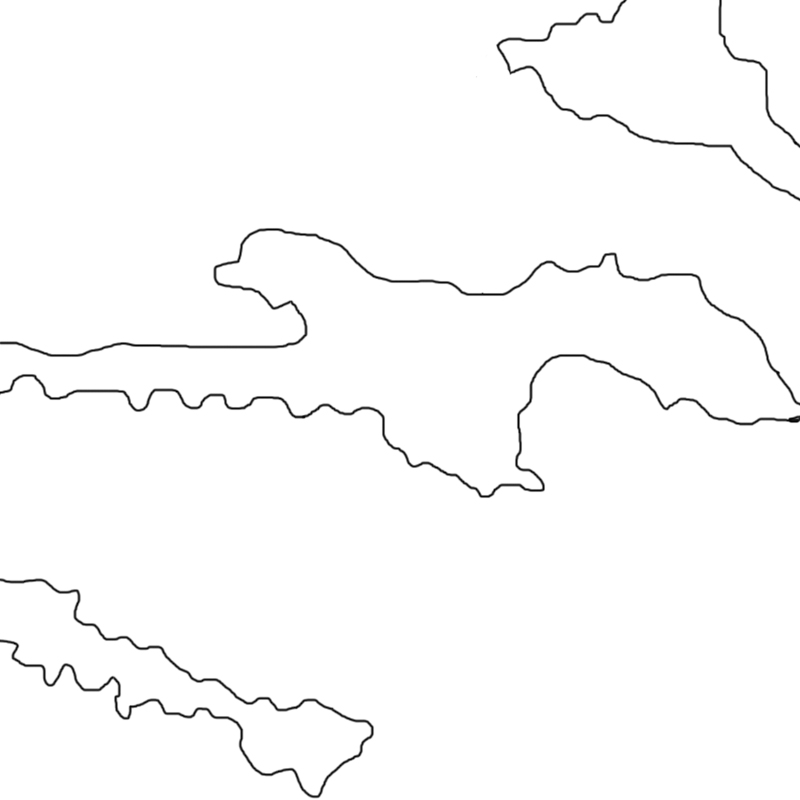

Supplement: Data S1 [file peerj-06-5789-s002.zip › C3/Image0015 GRID.jpg]

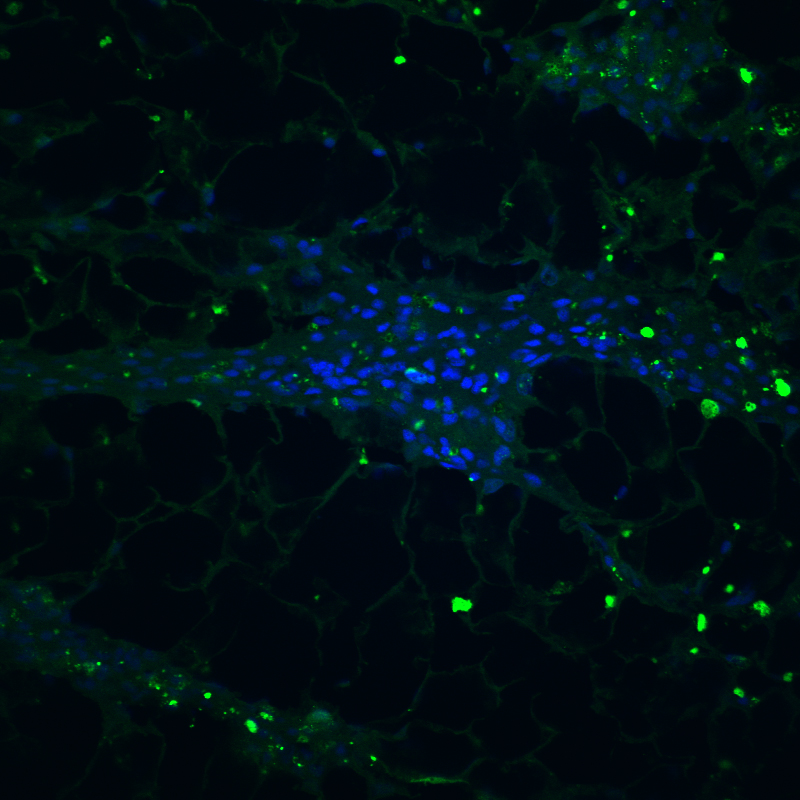

Supplement: Data S1 [file peerj-06-5789-s002.zip › C3/Image0015.jpg]

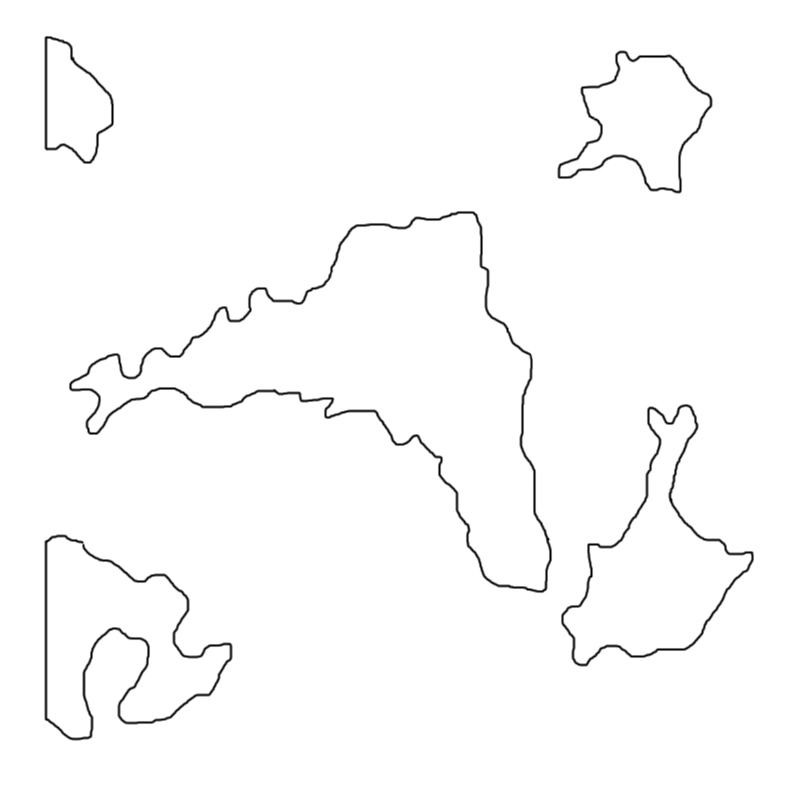

Supplement: Data S1 [file peerj-06-5789-s002.zip › C3/Image0017 GRID.jpg]

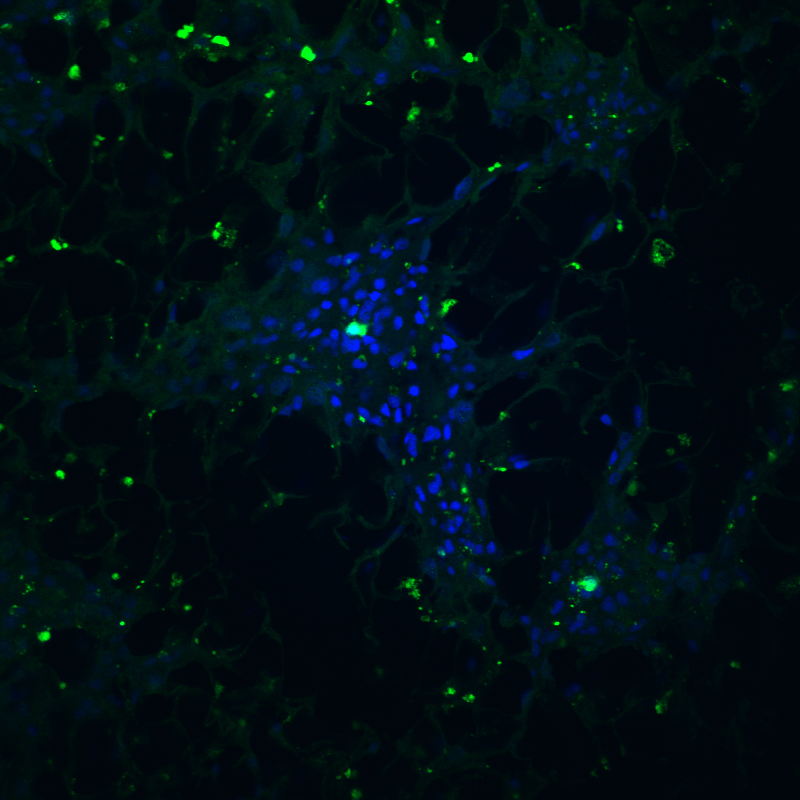

Supplement: Data S1 [file peerj-06-5789-s002.zip › C3/Image0017.jpg]

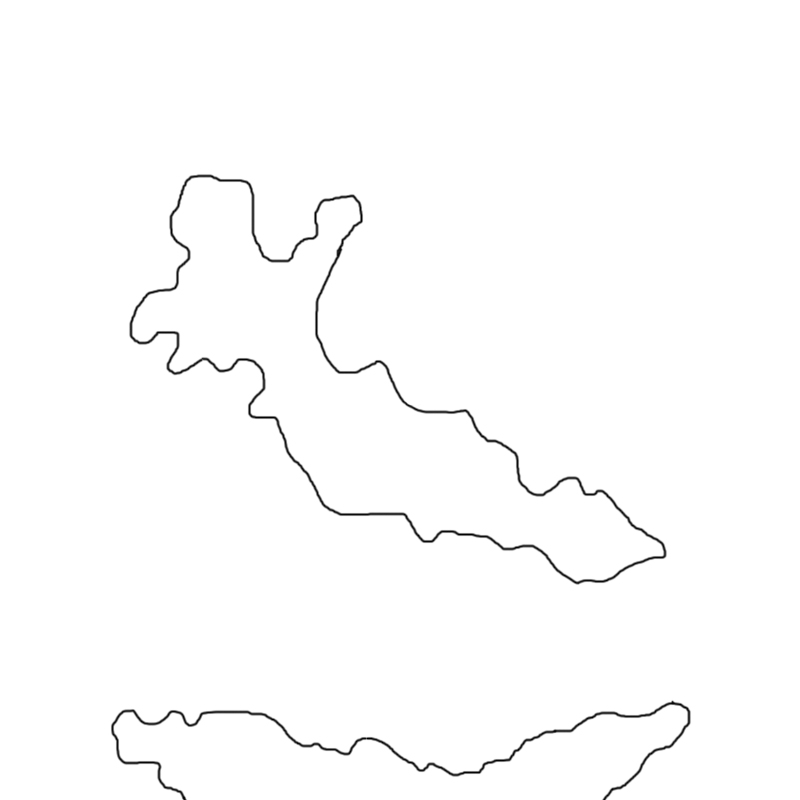

Supplement: Data S1 [file peerj-06-5789-s002.zip › C3/Image0018 GRID.jpg]

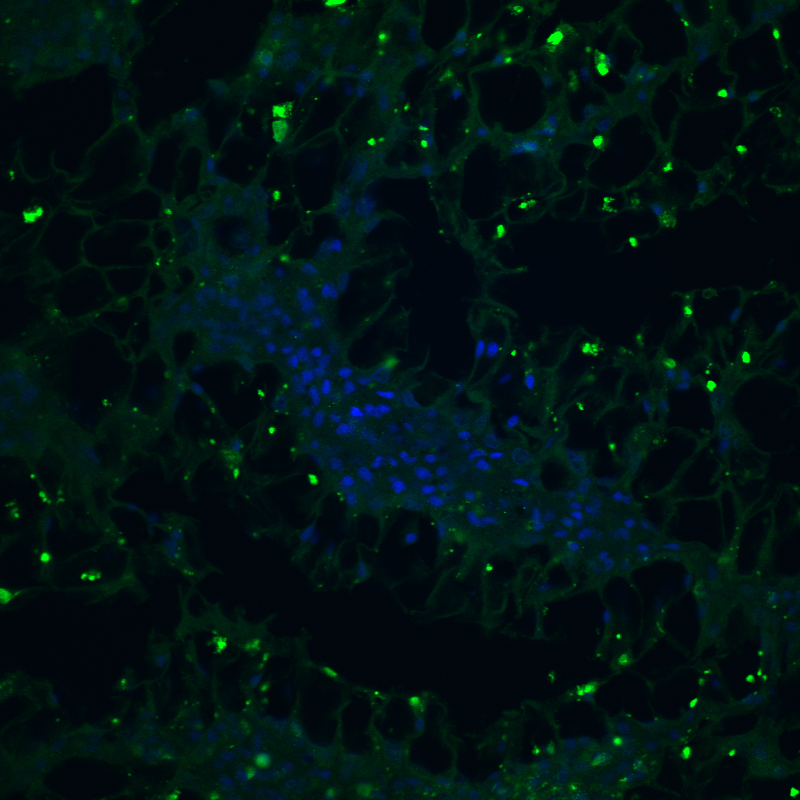

Supplement: Data S1 [file peerj-06-5789-s002.zip › C3/Image0018.jpg]

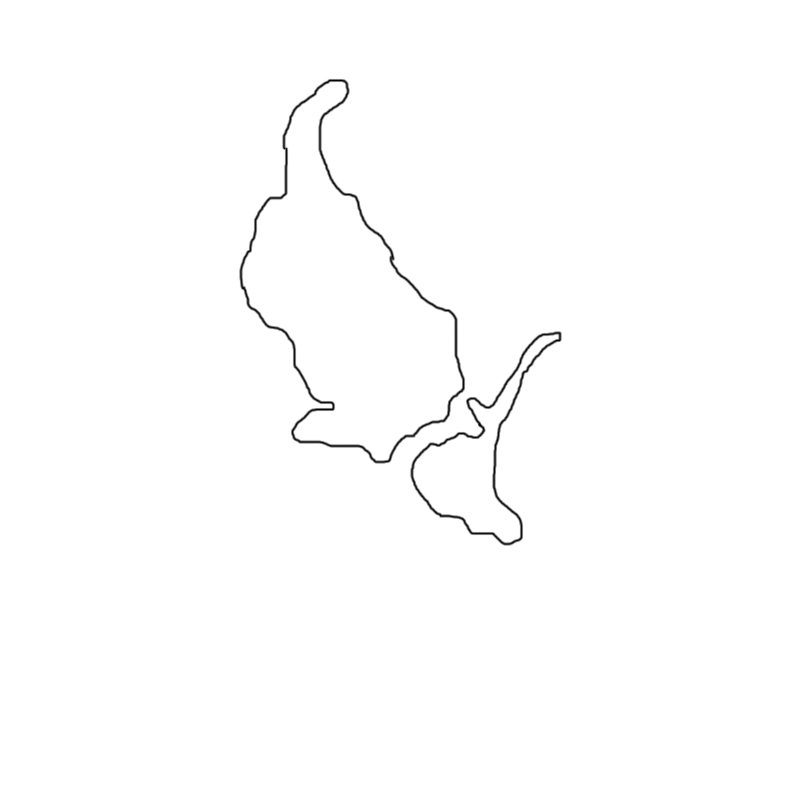

Supplement: Data S1 [file peerj-06-5789-s002.zip › C3/Image0019 GRID.jpg]

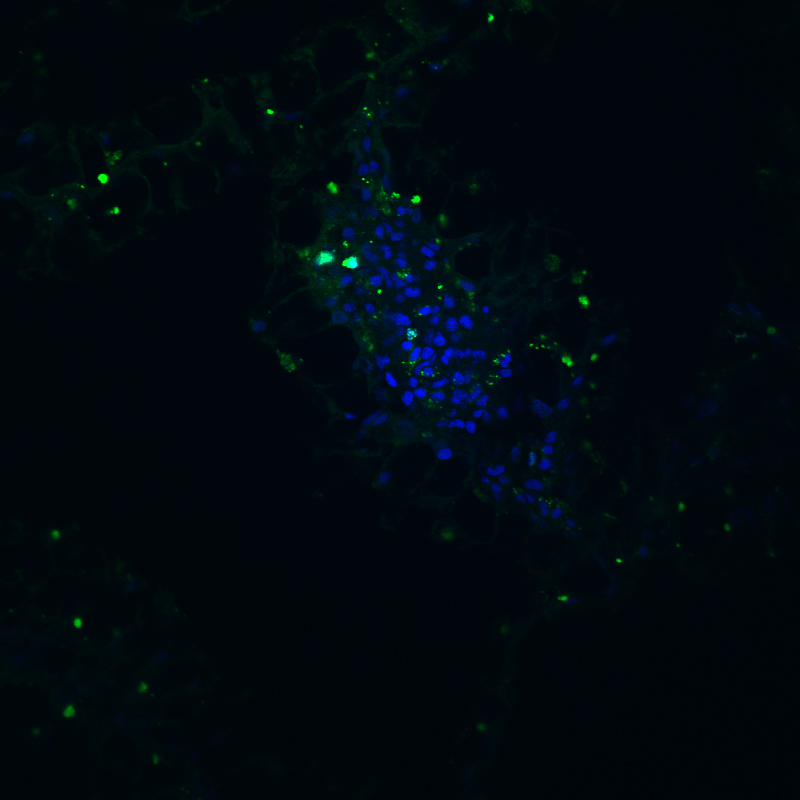

Supplement: Data S1 [file peerj-06-5789-s002.zip › C3/Image0019.jpg]

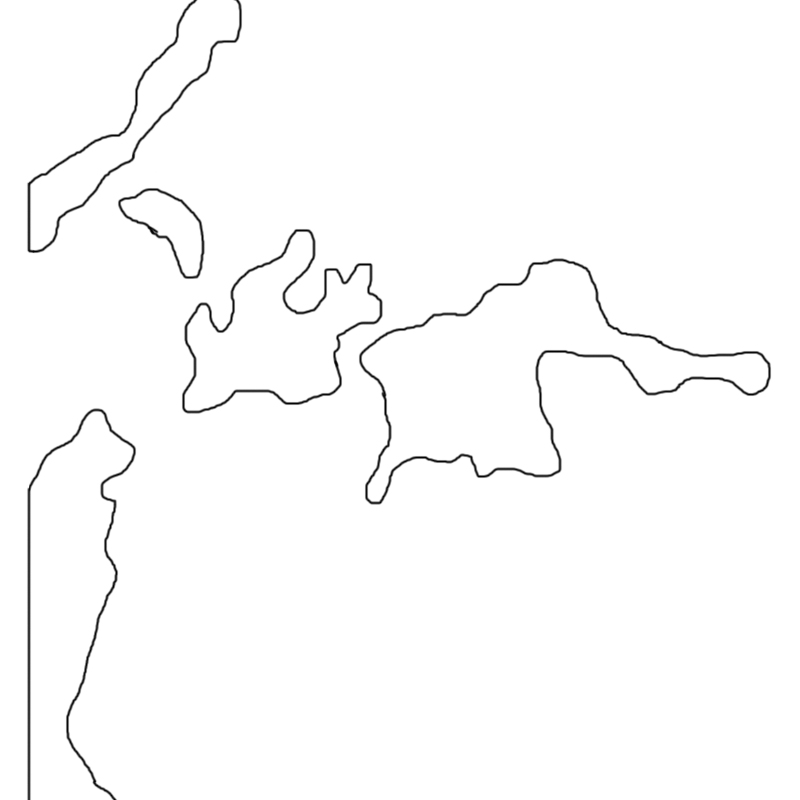

Supplement: Data S1 [file peerj-06-5789-s002.zip › C3/Image0020 GRID.jpg]

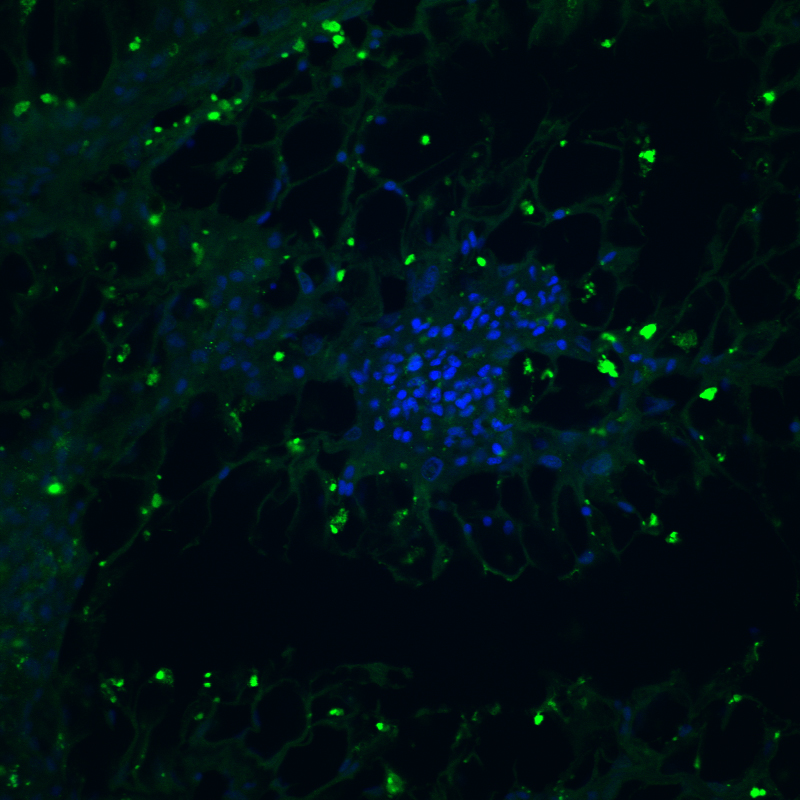

Supplement: Data S1 [file peerj-06-5789-s002.zip › C3/Image0020.jpg]

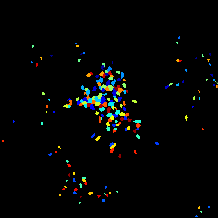

Supplement: Data S1 [file peerj-06-5789-s002.zip › C4/Im21 Nuclei.png]

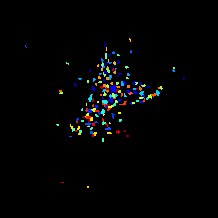

Supplement: Data S1 [file peerj-06-5789-s002.zip › C4/Im22 Nuclei.png]

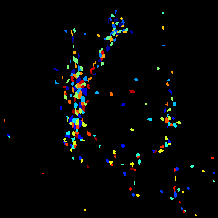

Supplement: Data S1 [file peerj-06-5789-s002.zip › C4/Im23 Nuclei.png]

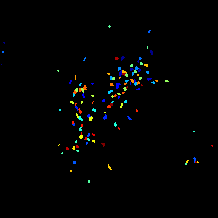

Supplement: Data S1 [file peerj-06-5789-s002.zip › C4/Im24 Nuclei.png]

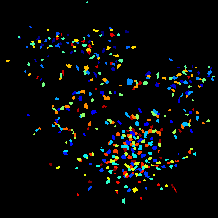

Supplement: Data S1 [file peerj-06-5789-s002.zip › C4/Im25 Nuclei.png]

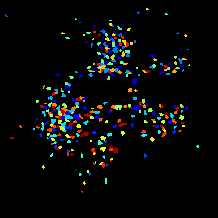

Supplement: Data S1 [file peerj-06-5789-s002.zip › C4/Im26 Nuclei.png]

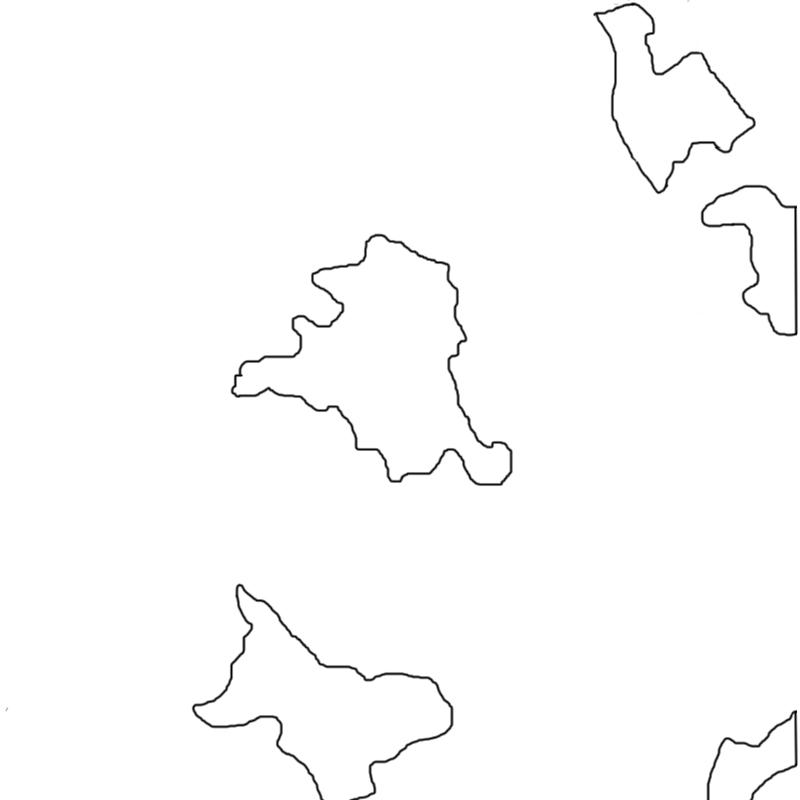

Supplement: Data S1 [file peerj-06-5789-s002.zip › C4/Image0021 GRID.jpg]

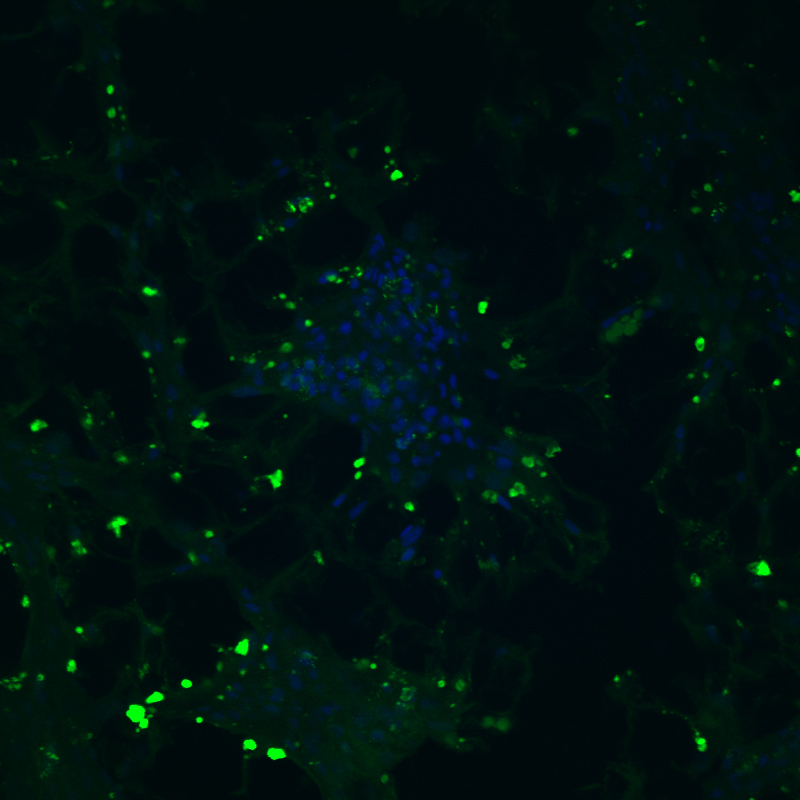

Supplement: Data S1 [file peerj-06-5789-s002.zip › C4/Image0021.jpg]

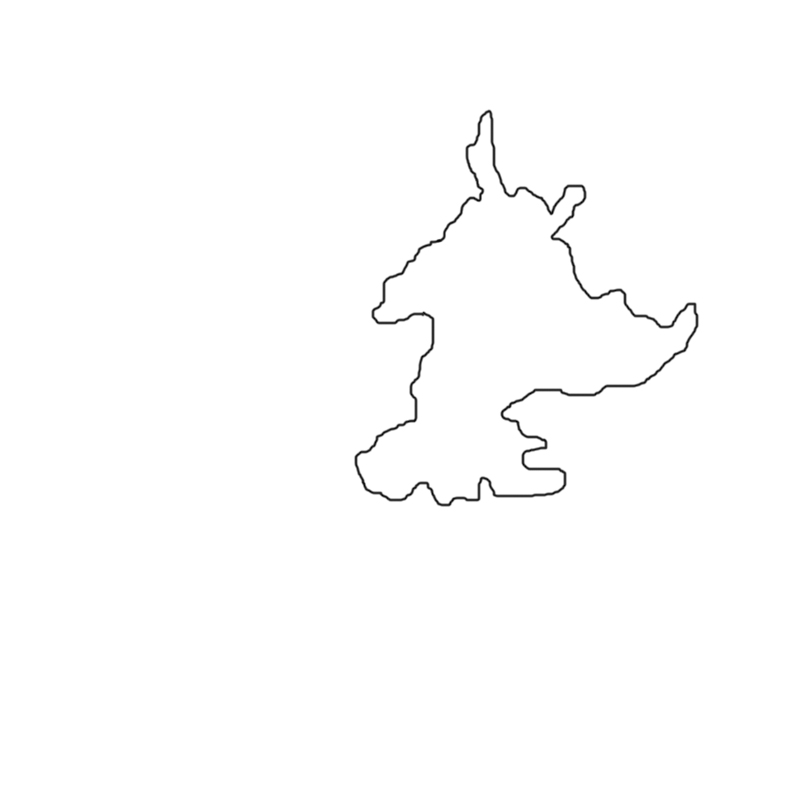

Supplement: Data S1 [file peerj-06-5789-s002.zip › C4/Image0022 GRID.jpg]

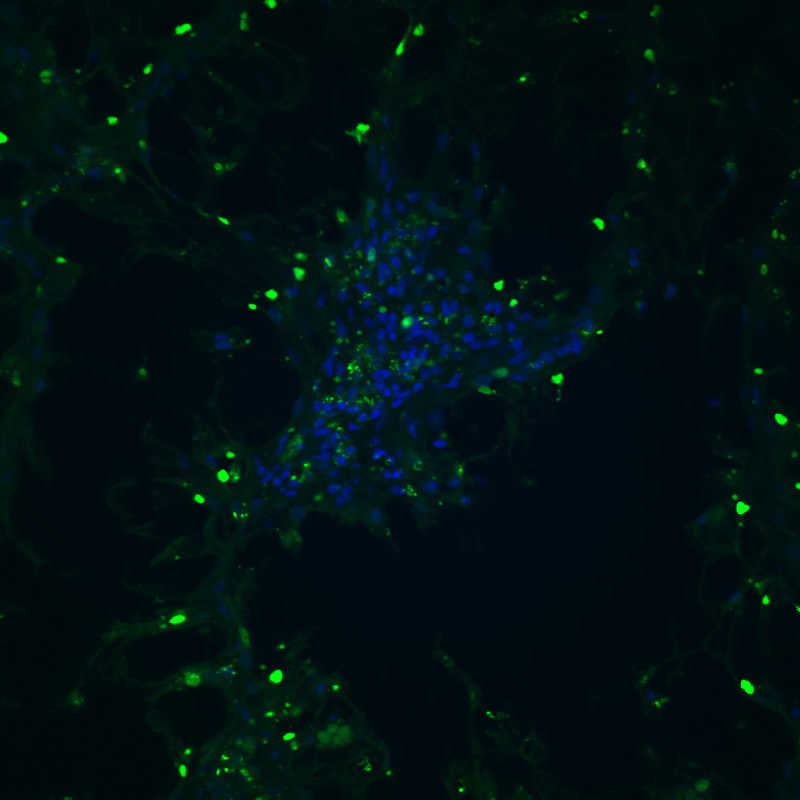

Supplement: Data S1 [file peerj-06-5789-s002.zip › C4/Image0022.jpg]

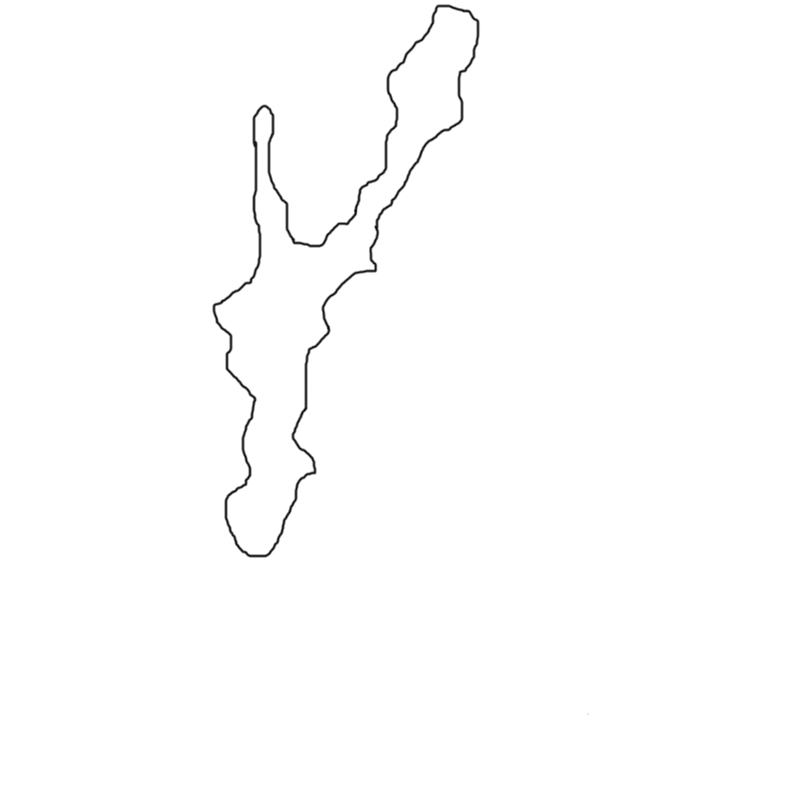

Supplement: Data S1 [file peerj-06-5789-s002.zip › C4/Image0023 GRID.jpg]

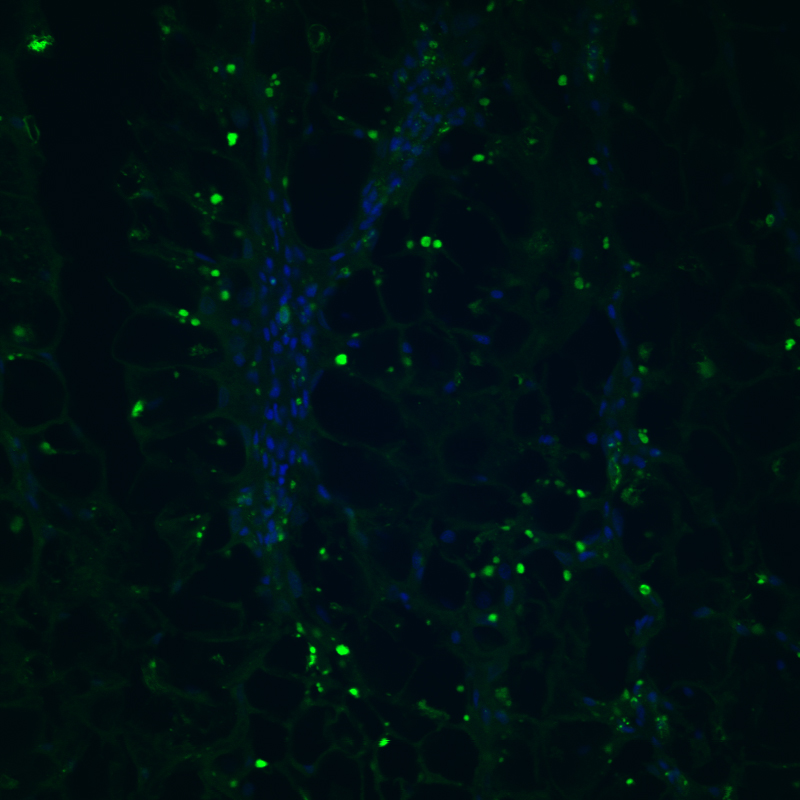

Supplement: Data S1 [file peerj-06-5789-s002.zip › C4/Image0023.jpg]

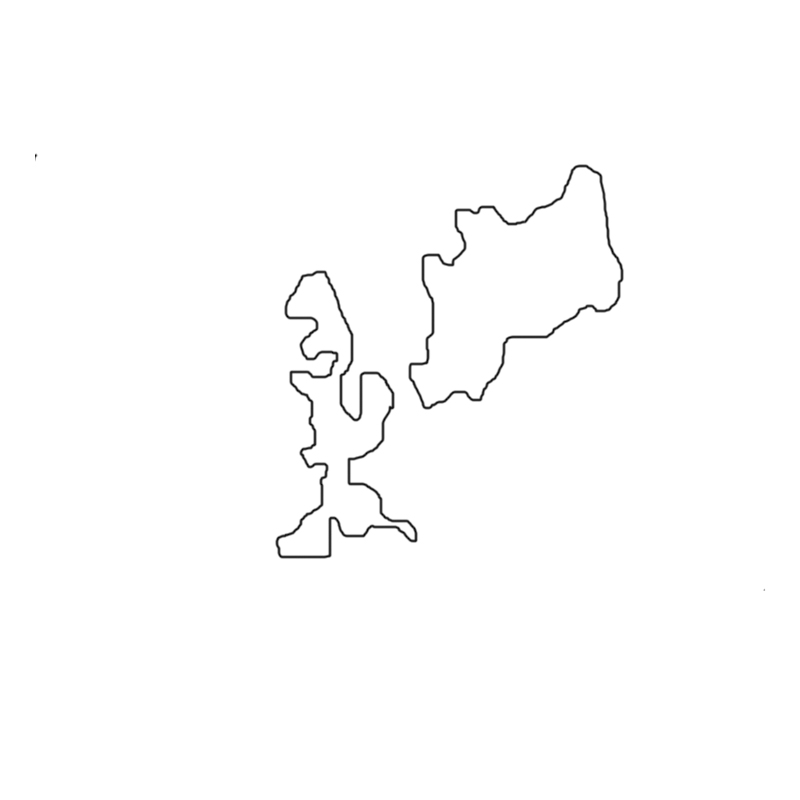

Supplement: Data S1 [file peerj-06-5789-s002.zip › C4/Image0024 GRID.jpg]

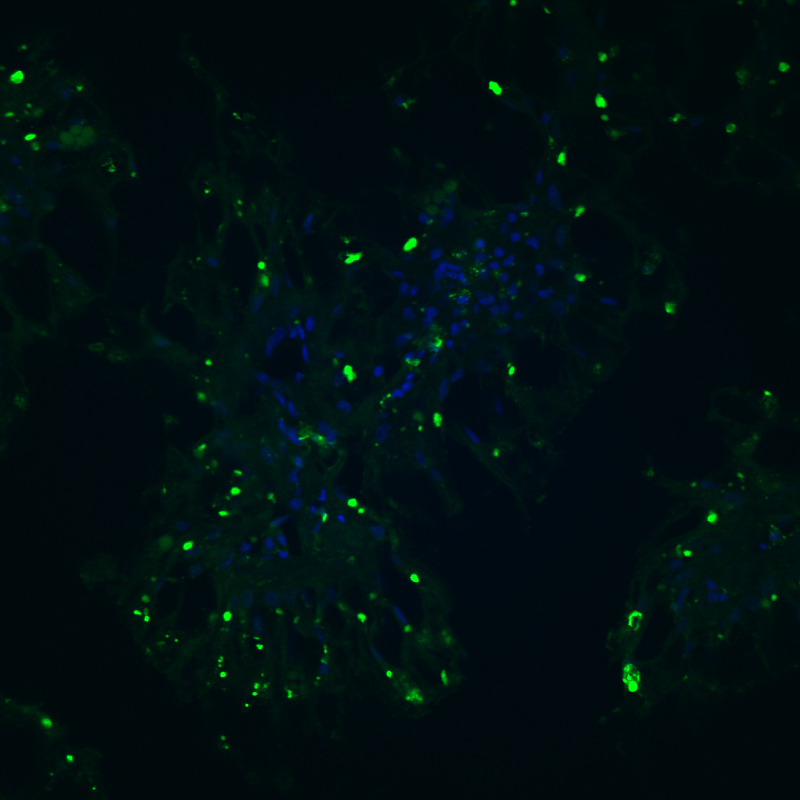

Supplement: Data S1 [file peerj-06-5789-s002.zip › C4/Image0024.jpg]

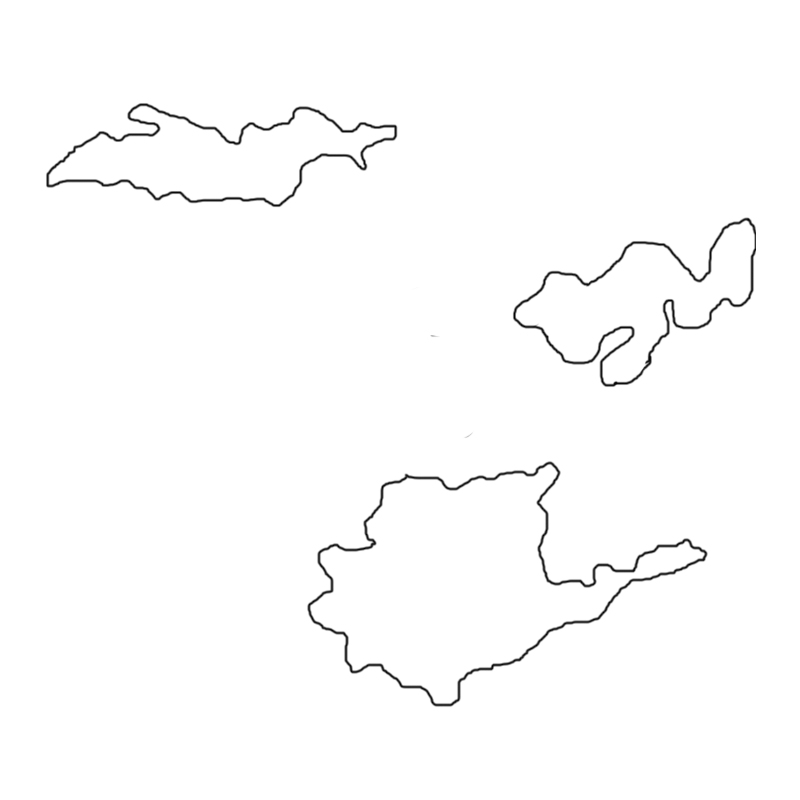

Supplement: Data S1 [file peerj-06-5789-s002.zip › C4/Image0025 GRID.jpg]

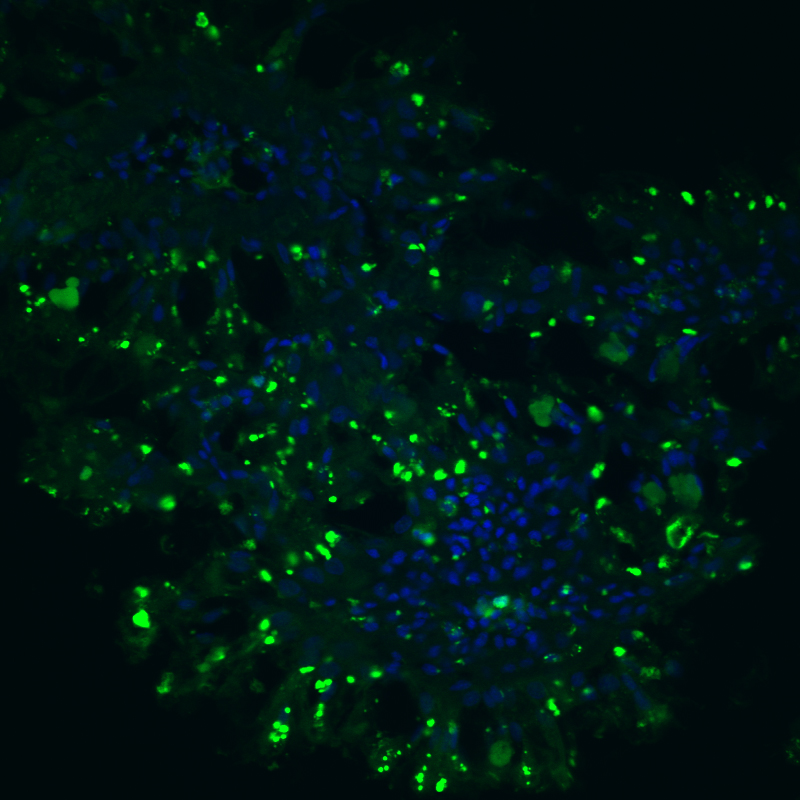

Supplement: Data S1 [file peerj-06-5789-s002.zip › C4/Image0025.jpg]

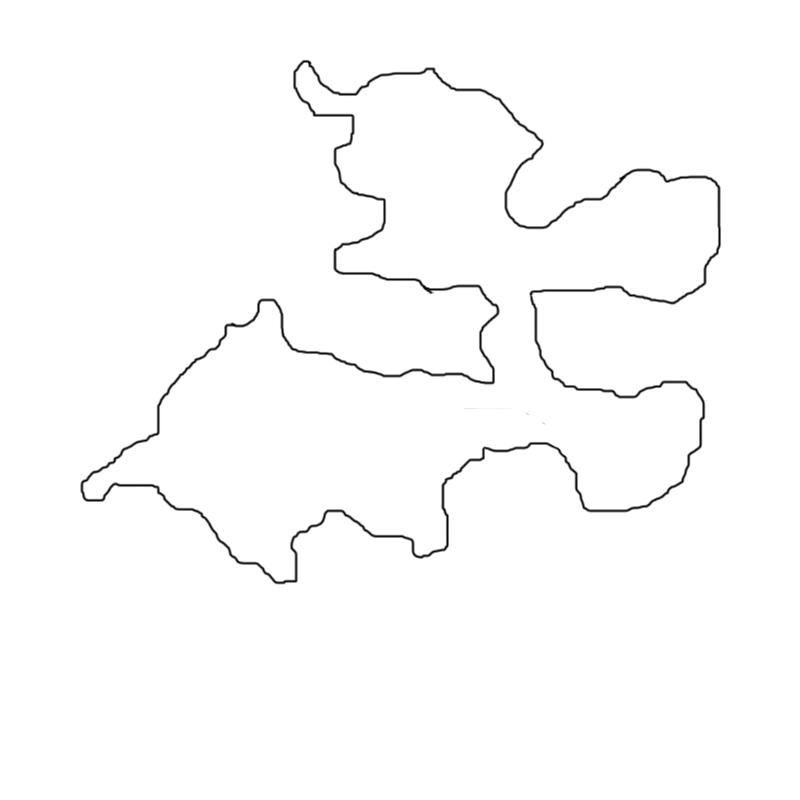

Supplement: Data S1 [file peerj-06-5789-s002.zip › C4/Image0026 GRID.jpg]

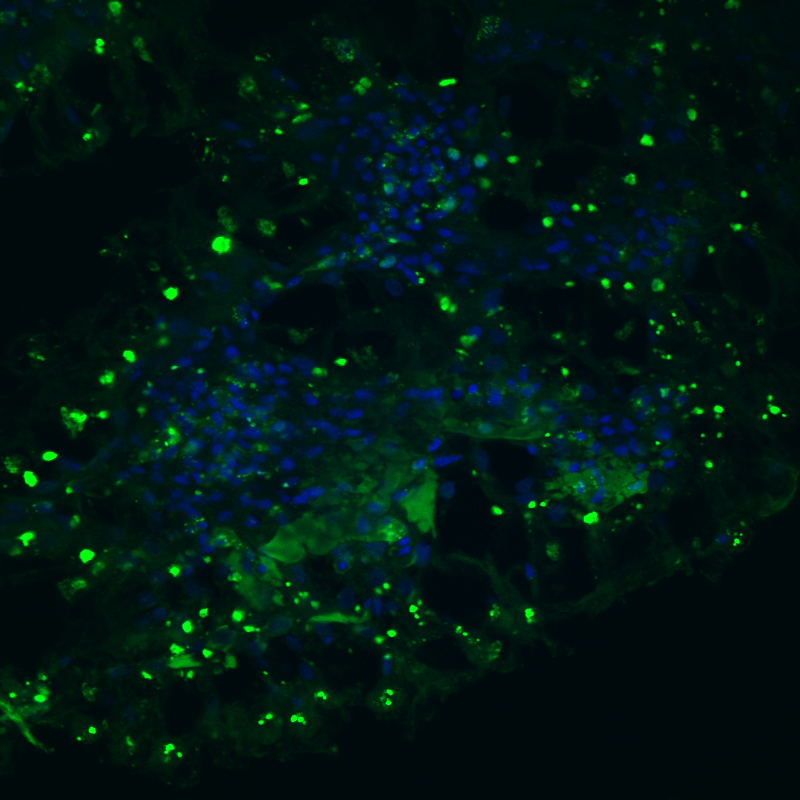

Supplement: Data S1 [file peerj-06-5789-s002.zip › C4/Image0026.jpg]

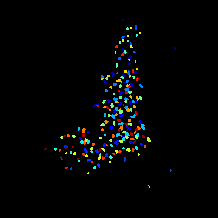

Supplement: Data S1 [file peerj-06-5789-s002.zip › C5/Im27 Nuclei.png]

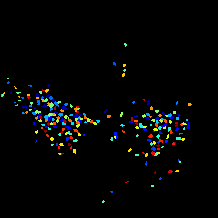

Supplement: Data S1 [file peerj-06-5789-s002.zip › C5/Im28 Nuclei.png]

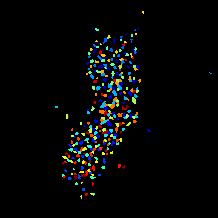

Supplement: Data S1 [file peerj-06-5789-s002.zip › C5/Im29 Nuclei.png]

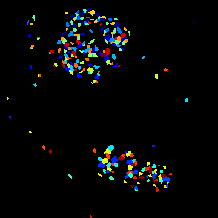

Supplement: Data S1 [file peerj-06-5789-s002.zip › C5/Im30 Nuclei.png]

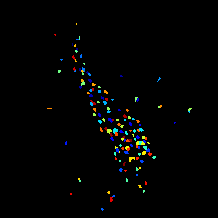

Supplement: Data S1 [file peerj-06-5789-s002.zip › C5/Im31 Nuclei.png]

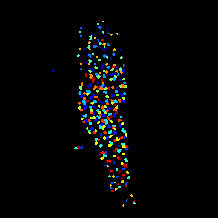

Supplement: Data S1 [file peerj-06-5789-s002.zip › C5/Im32 Nuclei.png]

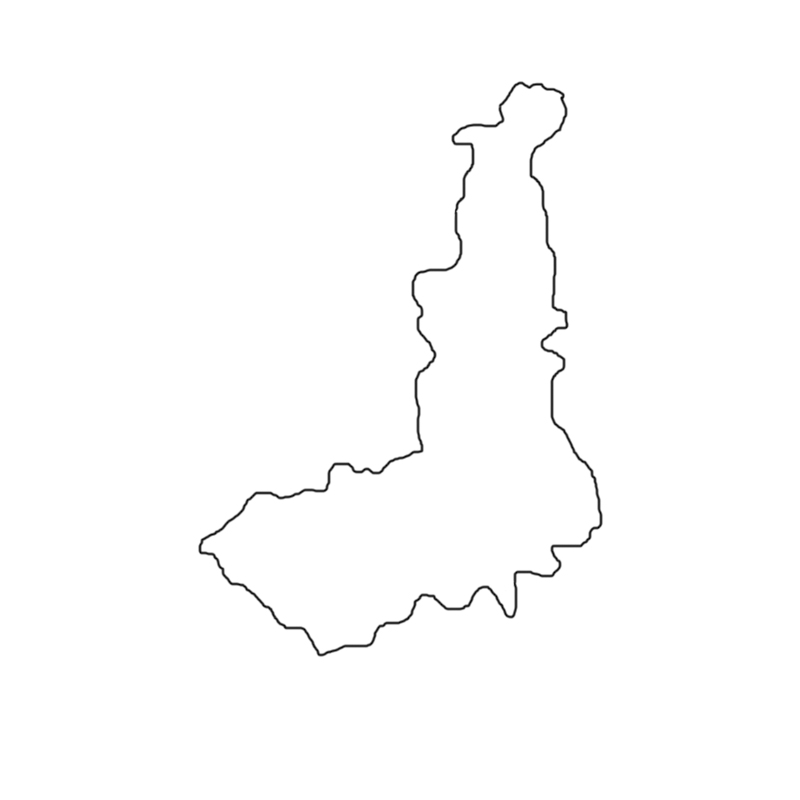

Supplement: Data S1 [file peerj-06-5789-s002.zip › C5/Image0027 GRID.jpg]

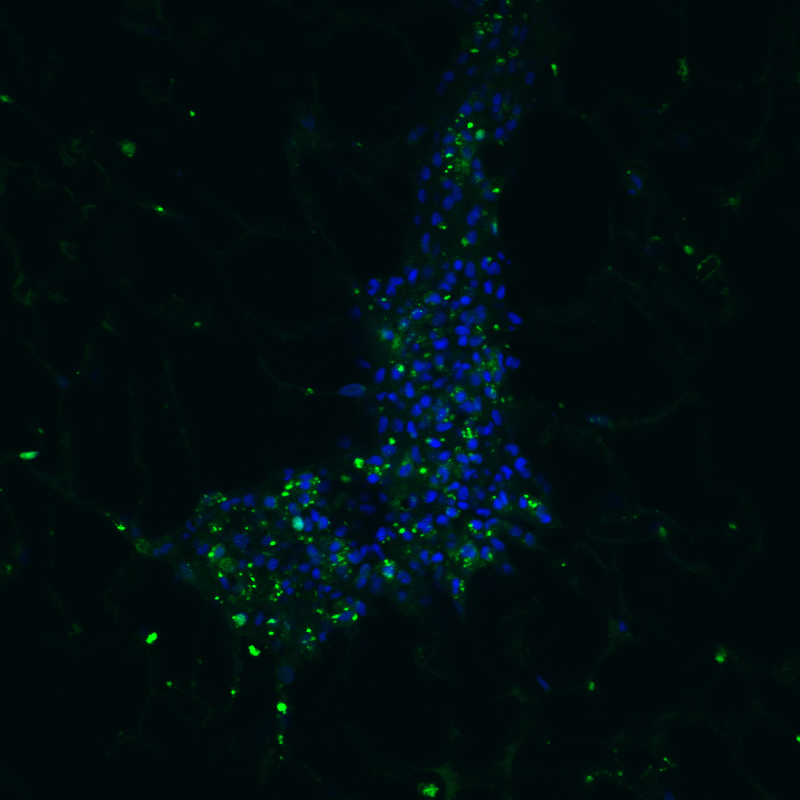

Supplement: Data S1 [file peerj-06-5789-s002.zip › C5/Image0027.jpg]

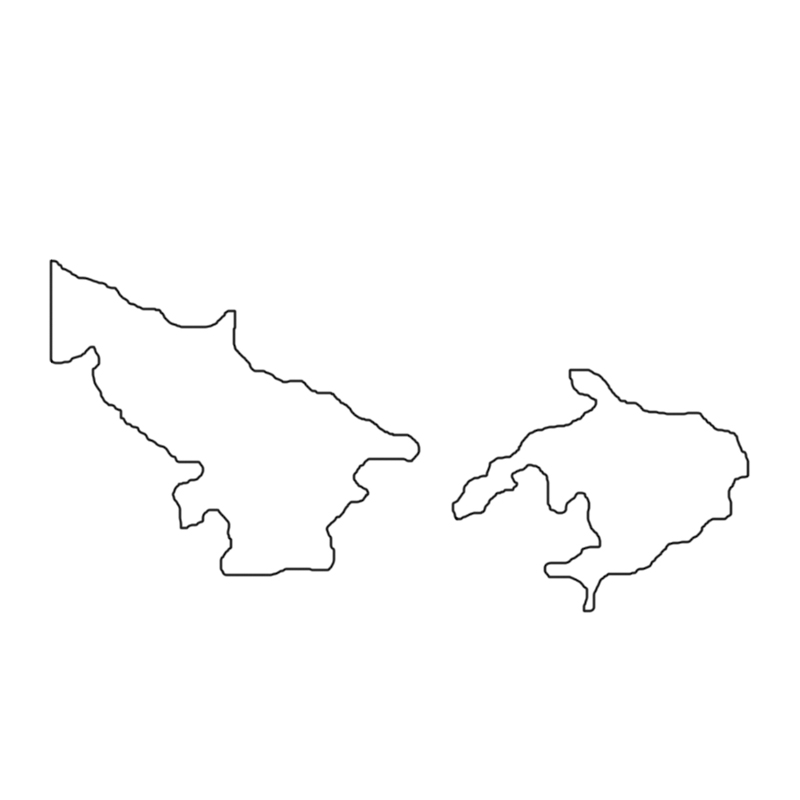

Supplement: Data S1 [file peerj-06-5789-s002.zip › C5/Image0028 GRID.jpg]

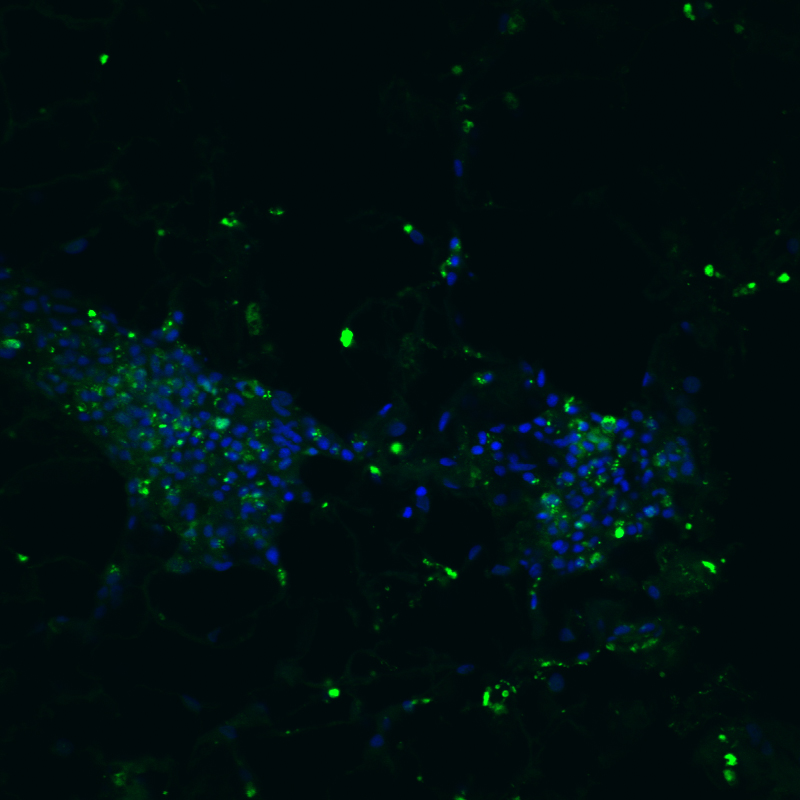

Supplement: Data S1 [file peerj-06-5789-s002.zip › C5/Image0028.jpg]

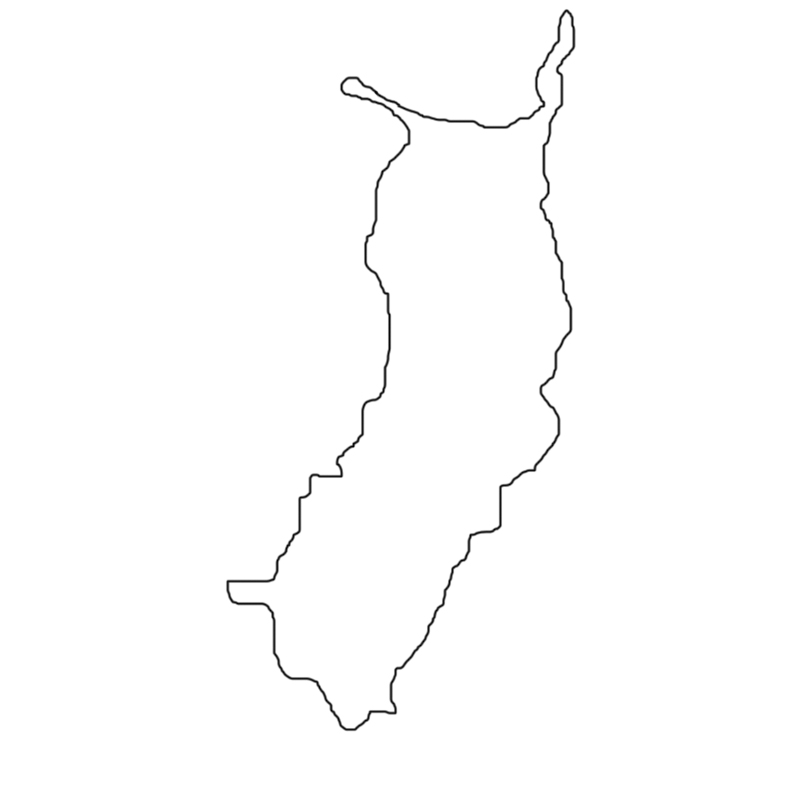

Supplement: Data S1 [file peerj-06-5789-s002.zip › C5/Image0029 GRID.jpg]

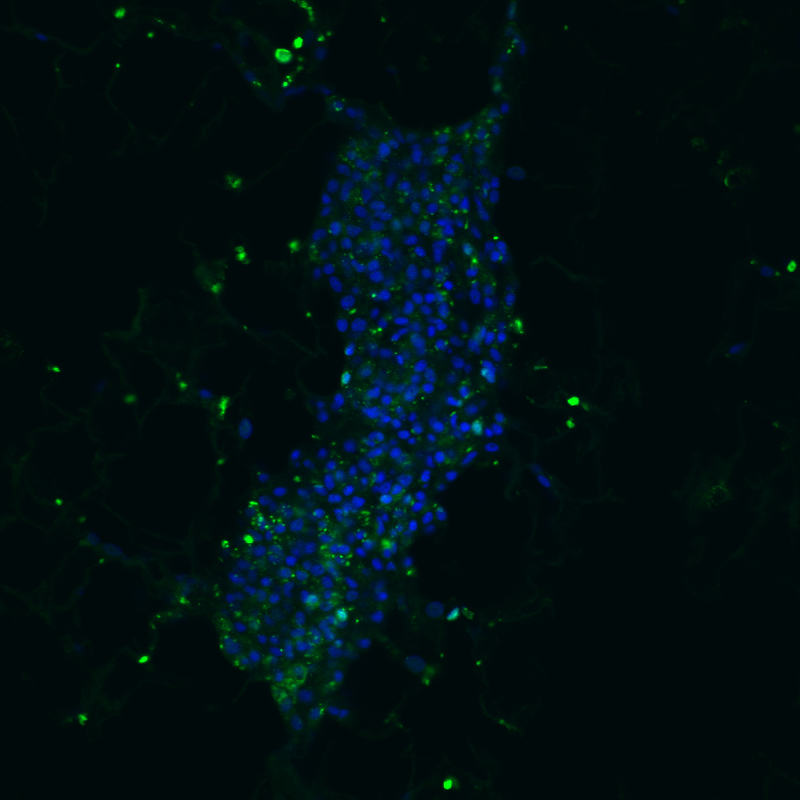

Supplement: Data S1 [file peerj-06-5789-s002.zip › C5/Image0029.jpg]

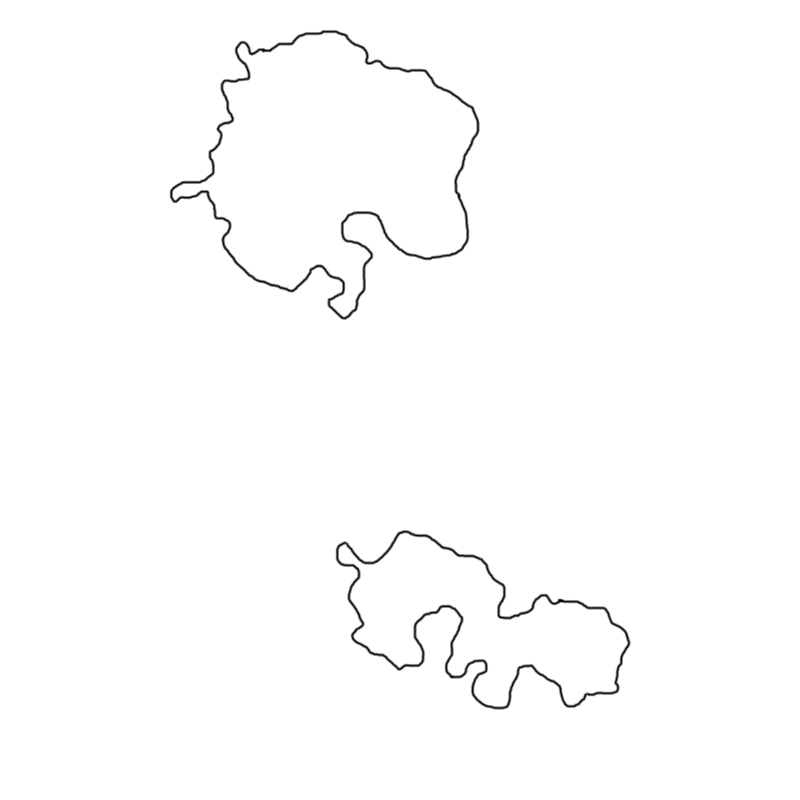

Supplement: Data S1 [file peerj-06-5789-s002.zip › C5/Image0030 GRID.jpg]

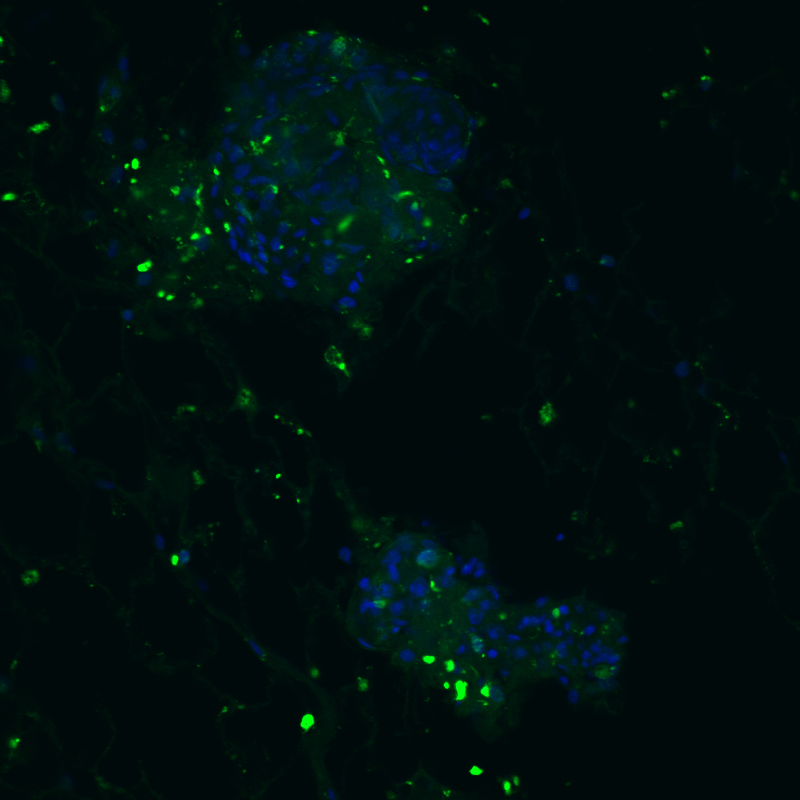

Supplement: Data S1 [file peerj-06-5789-s002.zip › C5/Image0030.jpg]

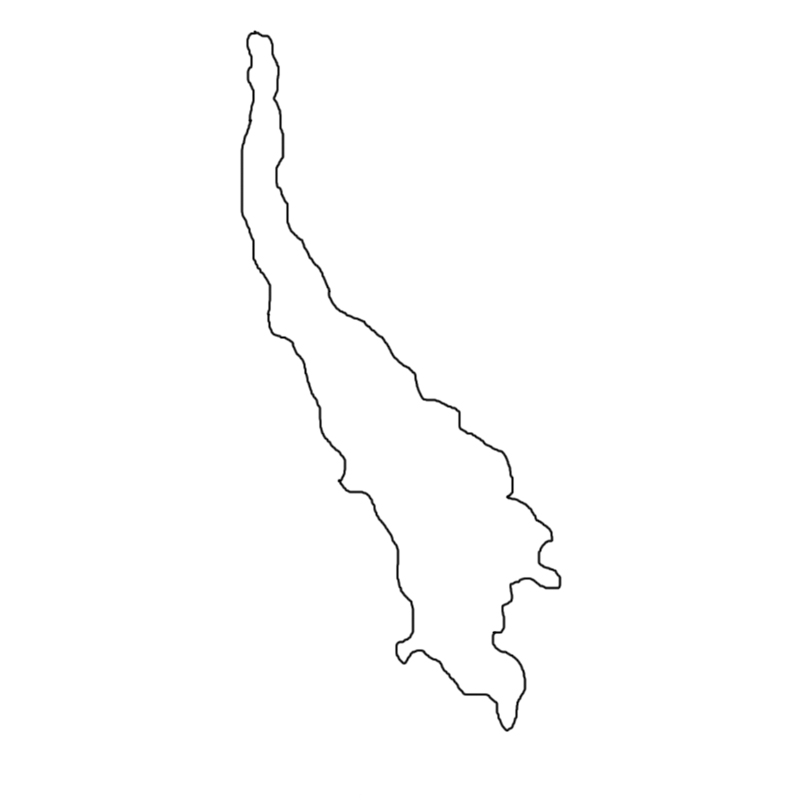

Supplement: Data S1 [file peerj-06-5789-s002.zip › C5/Image0031 GRID.jpg]

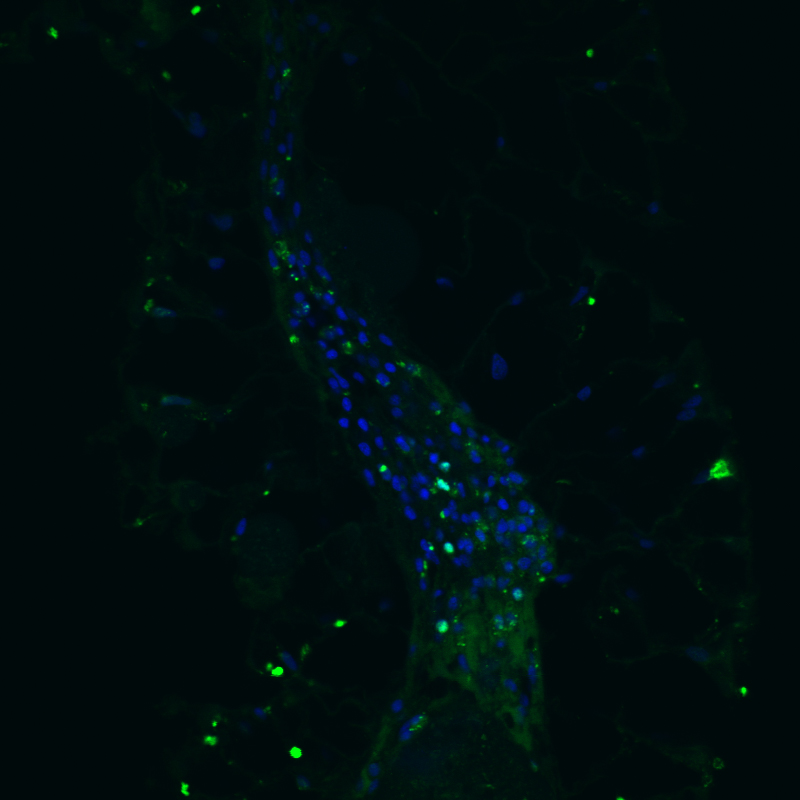

Supplement: Data S1 [file peerj-06-5789-s002.zip › C5/Image0031.jpg]

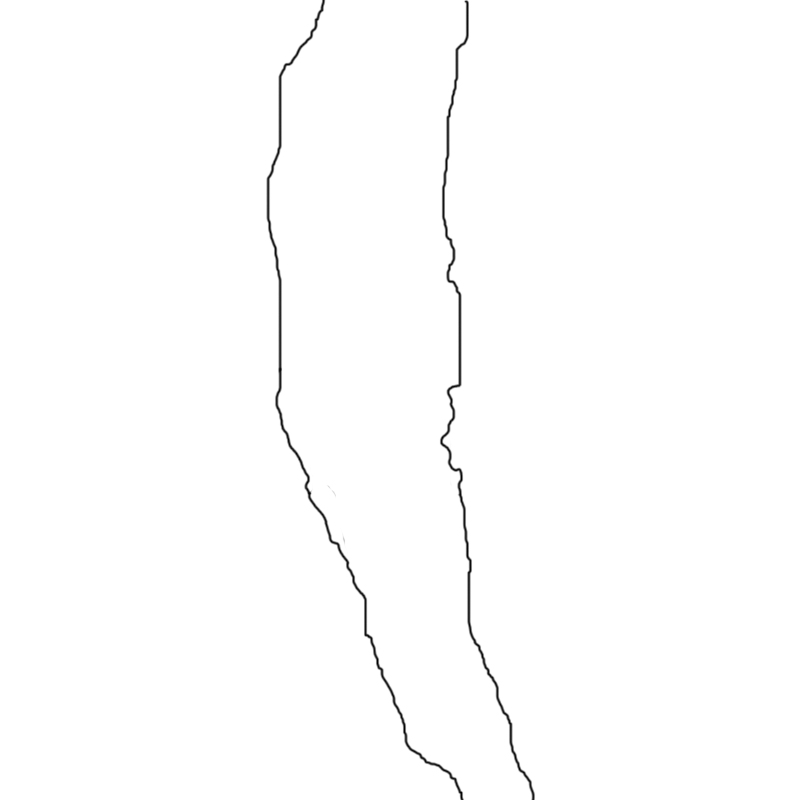

Supplement: Data S1 [file peerj-06-5789-s002.zip › C5/Image0032 GRID.jpg]

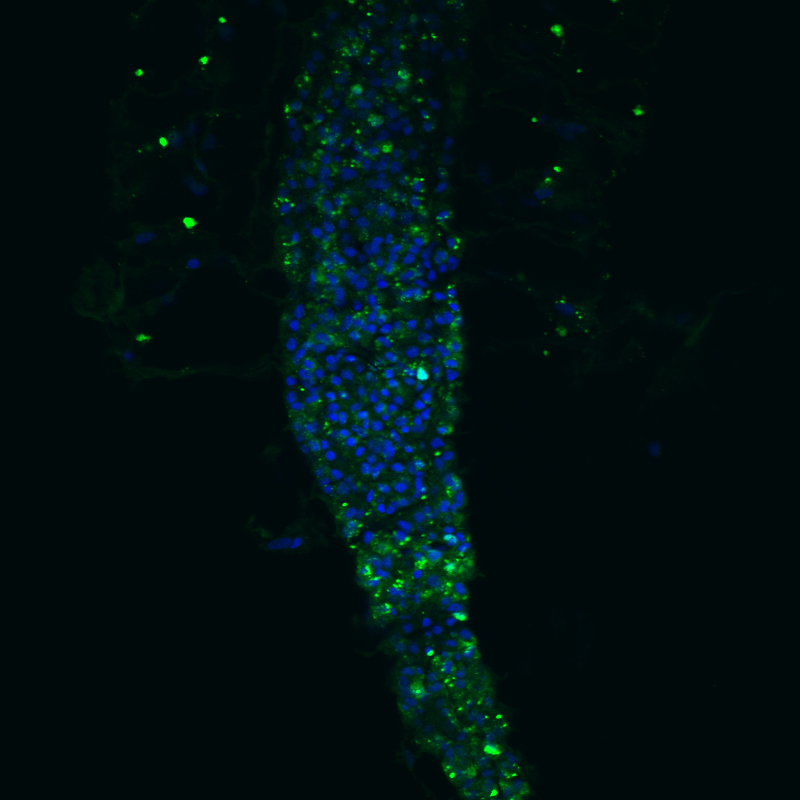

Supplement: Data S1 [file peerj-06-5789-s002.zip › C5/Image0032.jpg]

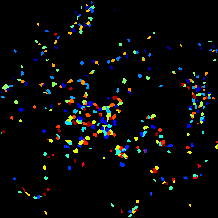

Supplement: Data S1 [file peerj-06-5789-s002.zip › C6/Im01 Nuclei.png]

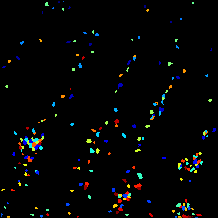

Supplement: Data S1 [file peerj-06-5789-s002.zip › C6/Im02 Nuclei.png]

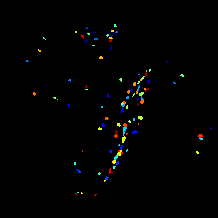

Supplement: Data S1 [file peerj-06-5789-s002.zip › C6/Im04 Nuclei.png]

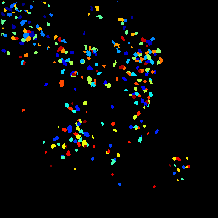

Supplement: Data S1 [file peerj-06-5789-s002.zip › C6/Im05 Nuclei.png]

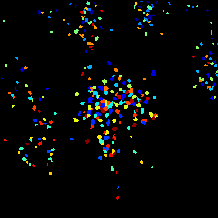

Supplement: Data S1 [file peerj-06-5789-s002.zip › C6/Im06 Nuclei.png]

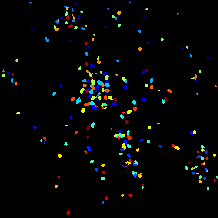

Supplement: Data S1 [file peerj-06-5789-s002.zip › C6/Im07 Nuclei.png]

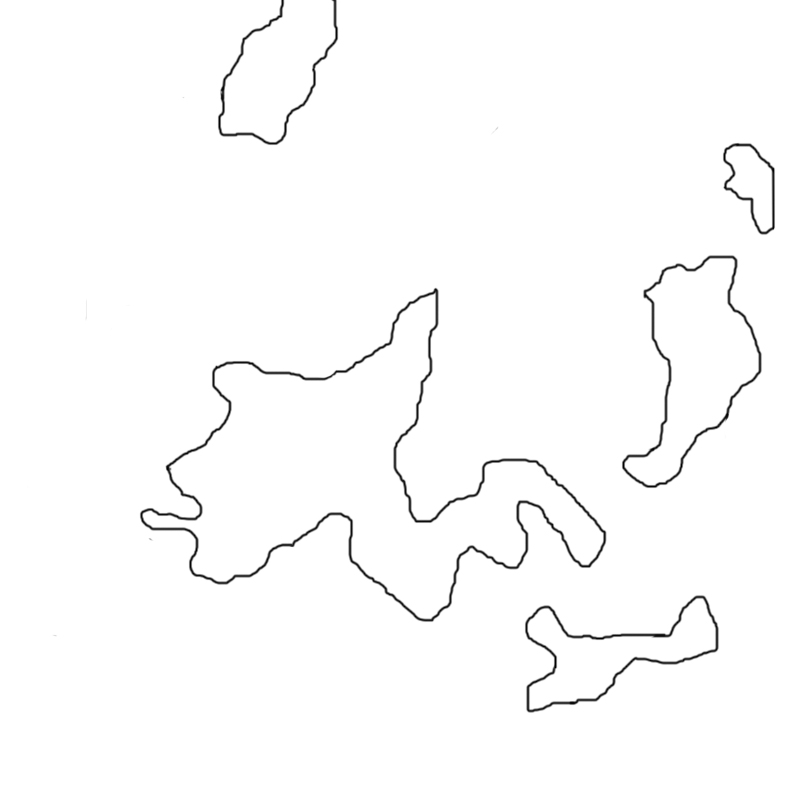

Supplement: Data S1 [file peerj-06-5789-s002.zip › C6/Image0001 GRID.jpg]

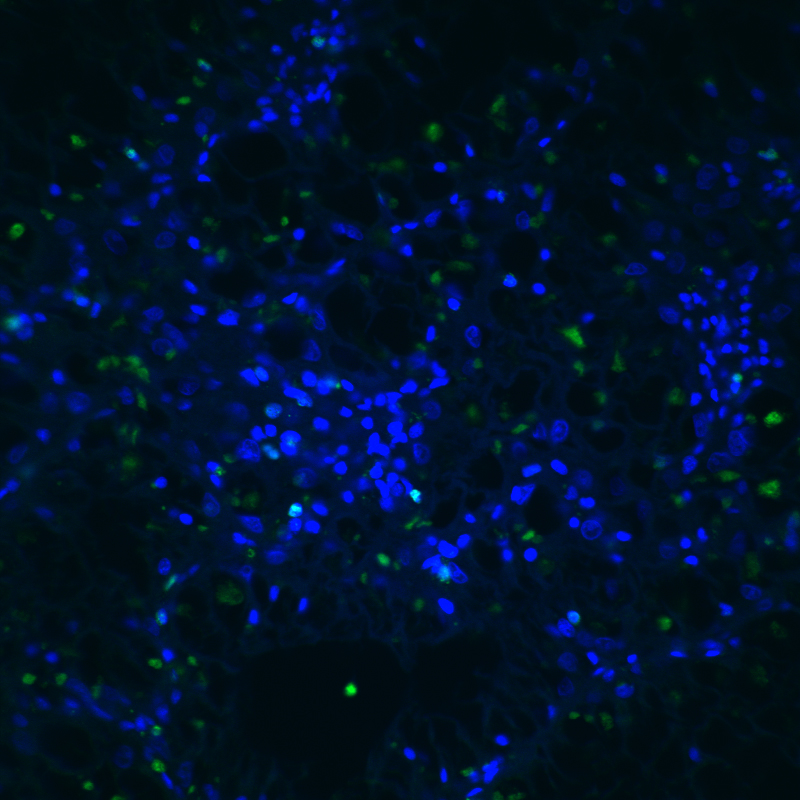

Supplement: Data S1 [file peerj-06-5789-s002.zip › C6/Image0001.jpg]

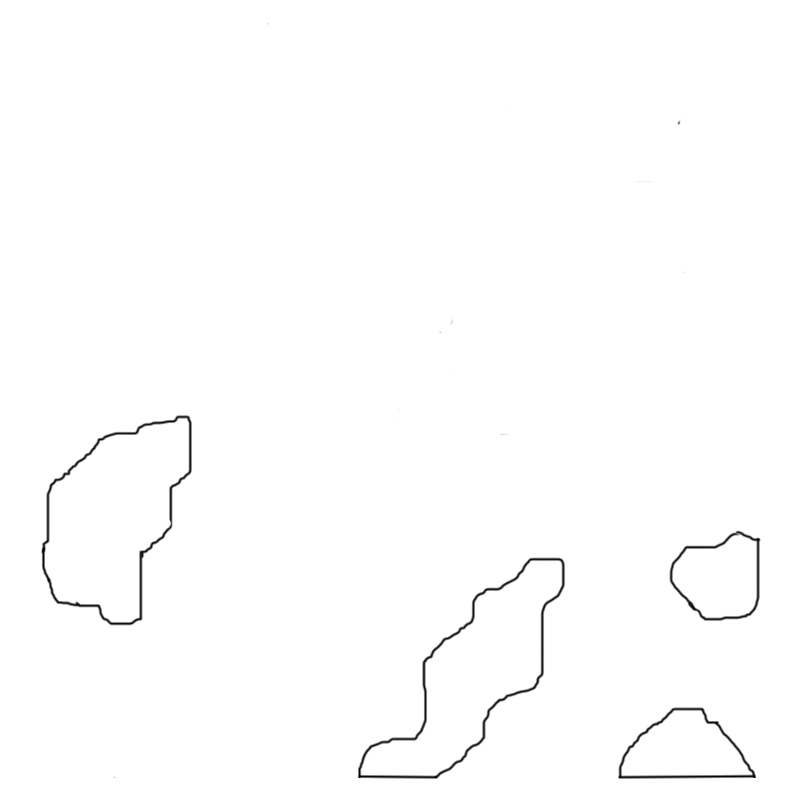

Supplement: Data S1 [file peerj-06-5789-s002.zip › C6/Image0002 GRID.jpg]

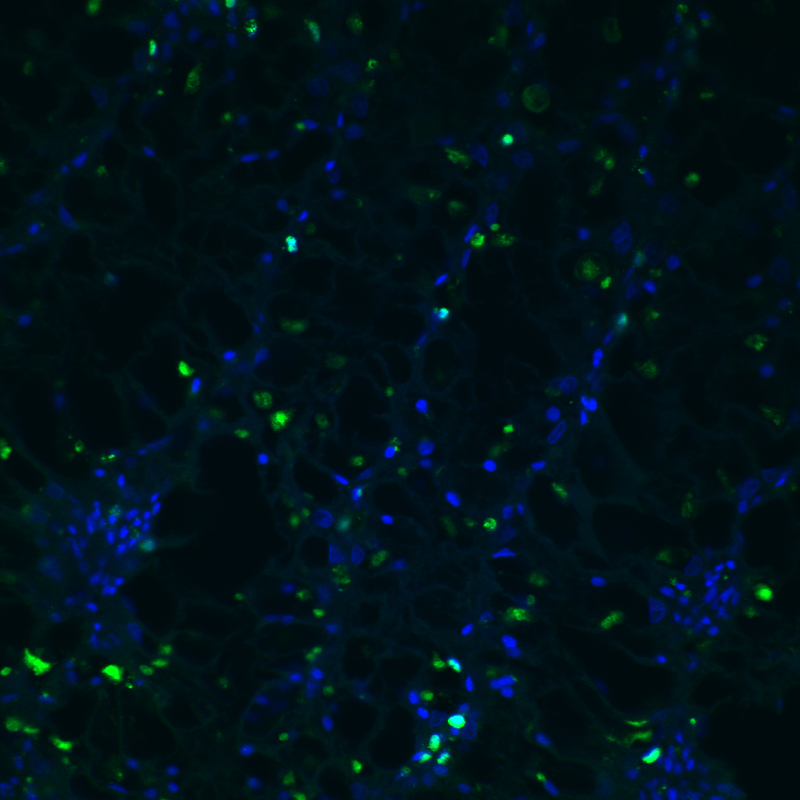

Supplement: Data S1 [file peerj-06-5789-s002.zip › C6/Image0002.jpg]
